# Freshwater mussels house a diverse mussel-associated leech assemblage

**DOI:** 10.1038/s41598-019-52688-3

**Published:** 2019-11-11

**Authors:** Ivan N. Bolotov, Anna L. Klass, Alexander V. Kondakov, Ilya V. Vikhrev, Yulia V. Bespalaya, Mikhail Yu Gofarov, Boris Yu Filippov, Arthur E. Bogan, Manuel Lopes-Lima, Zau Lunn, Nyein Chan, Olga V. Aksenova, Gennady A. Dvoryankin, Yulia E. Chapurina, Sang Ki Kim, Yulia S. Kolosova, Ekaterina S. Konopleva, Jin Hee Lee, Alexander A. Makhrov, Dmitry M. Palatov, Elena M. Sayenko, Vitaly M. Spitsyn, Svetlana E. Sokolova, Alena A. Tomilova, Than Win, Natalia A. Zubrii, Maxim V. Vinarski

**Affiliations:** 1 Northern Arctic Federal University, Arkhangelsk, Russia; 2 Federal Center for Integrated Arctic Research, Russian Academy of Sciences, Arkhangelsk, Russia; 3 Laboratory of Macroecology & Biogeography of Invertebrates, Saint Petersburg State University, Saint Petersburg, Russia; 4 Research Laboratory, North Carolina Museum of Natural Sciences, Raleigh, North Carolina United States of America; 5 CIBIO/InBIO – Research Center in Biodiversity and Genetic Resources, University of Porto, Campus Agrário de Vairão, Vairão, Portugal; 6 CIIMAR/CIMAR – Interdisciplinary Centre of Marine and Environmental Research, University of Porto, Terminal de Cruzeiros do Porto de Leixões, Matosinhos, Portugal; 7 SSC/IUCN – Mollusc Specialist Group, Species Survival Commission, International Union for Conservation of Nature, Cambridge, United Kingdom; 8 Fauna & Flora International – Myanmar Program, Yangon, Myanmar; 9 Nakdonggang National Institute of Biological Resources, Gyeongsangbuk-do, Korea; 10 Daegu Science High School, Daegu, Korea; 11 A. N. Severtzov Institute of Ecology and Evolution, Russian Academy of Sciences, Moscow, Russia; 12 Lomonosov Moscow State University, Moscow, Russia; 13 Federal Scientific Center of the East Asia Terrestrial Biodiversity, Far Eastern Branch of the Russian Academy of Sciences, Vladivostok, Russia; 14 Department of Zoology, Hpa-An University, Hpa-An, Kayin State Myanmar

**Keywords:** Taxonomy, Biogeography, Zoology

## Abstract

Freshwater mussels (Unionida) are one of the most imperiled animal groups worldwide, revealing the fastest rates of extinction. Habitat degradation, river pollution and climate change are the primary causes of global decline. However, biological threats for freshwater mussels are still poorly known. Here, we describe a diverse ecological group of leeches (Hirudinea: Glossiphoniidae) inhabiting the mantle cavity of freshwater mussels. So far, examples of mussel-associated leech species are recorded from East Asia, Southeast Asia, India and Nepal, Africa, and North America. This group comprises a dozen glossiphoniid species with a hidden life style inside the mantle cavity of their hosts largely overlooked by researchers. We show that the association with freshwater mussels evolved independently in three leech clades, i.e. *Batracobdelloides*, *Hemiclepsis*, and *Placobdella*, at least since the Miocene. Seven mussel-associated leech species and two additional free-living taxa are described here as new to science.

## Introduction

Parasites and symbionts of freshwater mussels (Unionida) are poorly known^1,2^ representing an overlooked threat to this imperiled taxonomic group^3^. The freshwater mite family Unionicolidae is the most iconic and species-rich example of such symbiotic organisms having a global distribution and using a variety of freshwater mussel species as hosts^4,5^. The mayfly genera *Symbiocloeon* and *Mutelocloeon* (Baetidae) are another remarkable example of invertebrates being strongly associated with freshwater mussels, as their larvae are inhabitants of the mussel mantle cavity collecting food particles from the gill surface^6–8^. Recently, it was found that a plethora of other invertebrate taxa could be considered as endosymbionts, commensals or parasites of freshwater mussels including chironomids (Chironomidae), copepods (Copepoda), digenean and aspidogastrean trematodes (Trematoda), oligochaetes (Oligochaeta), and leeches (Hirudinea)^3^.

While the frequent presence of leeches in the mantle cavity of freshwater mussels has been recorded since the second half of the 19th century, it was initially regarded as an accidental phenomenon^9^. Two leech species inhabiting the mantle cavity of freshwater mussels were reported from North America^1,2^. *Placobdella montifera* was found within the mantle cavity of at least 15 freshwater mussel species (Unionidae)^10–12^. This leech species appears to be a host generalist^10,13^, while it was suggested that its feeding on freshwater mussels is doubtful ^12–14^. and that the only confirmed host records were several fish species and a turtle^10,13,14^. The rare occurrence of *Placobdella parasitica*, another leech species, inside the mantle cavity of freshwater mussels has also been reported^1^. The so-called “clandestine shelter” hypothesis explains the facultative association of these leech species with freshwater mussels as commensal relationships, in which leeches use mussels as a shelter^1,2,10–12,14^. The occasional records of other glossiphoniid leeches (i.e., two *Helobdella* and one *Glossiphonia* species) from freshwater mussels in the USA^11,12^ need further confirmation and research to be considered a mussel-leech association.

Conversely, *Batracobdella kasmiana*, an East Asian species, was found to be a possible obligate inhabitant of the mantle cavity of large freshwater mussels having a broad range covering Japan, the southern areas of the Russian Far East, continental China, and Taiwan^15–21^. It has been shown that freshwater mussels (Unionidae and Margaritiferidae) in the southern part of the Russian Far East are heavily infested by this leech species^21^. The high level of leech infestation was also discovered in *Lamprotula caveata* (Unionidae) from Poyang Lake, Yangtze Basin, China, with approximately 30% of mussel specimens being infested^22^. *Batracobdelloides reticulatus* is characterized as an inhabitant of the mantle cavity of freshwater mussels (Unionidae) in India^23–25^. *Batracobdelloides tricarinatus* was recorded from the mantle cavity of freshwater mussels (Unionidae and Iridinidae) in Africa^26,27^, but this association was regarded as incidental, because this leech species did not share any indication of feeding on mussels, even in prolonged starvation^26^. In summary, *Batracobdella kasmiana* and *Batracobdelloides reticulatus* seem to be the two possible obligate inhabitants of the mantle cavity of freshwater mussels known to date, while *B*. *tricarinatus* from Africa and the two *Placobdella* species from North America were considered occasional visitors of such unusual habitats. In all known cases, outlined above, feeding of leeches on their mussel hosts was never confirmed experimentally.

Here we report the unexpected discovery of a species-rich assemblage of leeches associated with freshwater mussels recovered during our field surveys throughout East Asia, Southeast Asia and East Africa from 2002-2018. This study aims to (1) provide a taxonomic revision of mussel-associated leeches by means of an integrative approach combining molecular, morphological, biogeographic, and ecological evidences; (2) estimate the origin of mussel-associated leeches and their biogeographic affinities using a two-locus fossil-calibrated phylogeny; (3) describe the life cycle of mussel-associated leeches and their host range; and (4) assess the prevalence and intensity of leech infestation of freshwater mussels from various freshwater drainages. As mussel-associated leeches, we consider the leech species that were consistently recorded from the mantle cavity of freshwater mussels (Unionida). As obligate mussel inhabitants (i.e. true mussel leeches), we consider mussel-associated leech species that probably use freshwater mussels as a secondary host and shelter in the earlier developmental stages of their life cycle, while the adult stage of such leech taxa uses freshwater fishes as the primary host to reach maturity. As facultative mussel inhabitants, we consider mussel-associated leech species that can complete their life cycle in open environments besides the mantle cavity of freshwater mussels, using freshwater fishes and other taxa as hosts, but were repeatedly found as mussel mantle cavity inhabitants.

## Results

### Integrative taxonomy of the mussel leech assemblage

**Figure 1:**
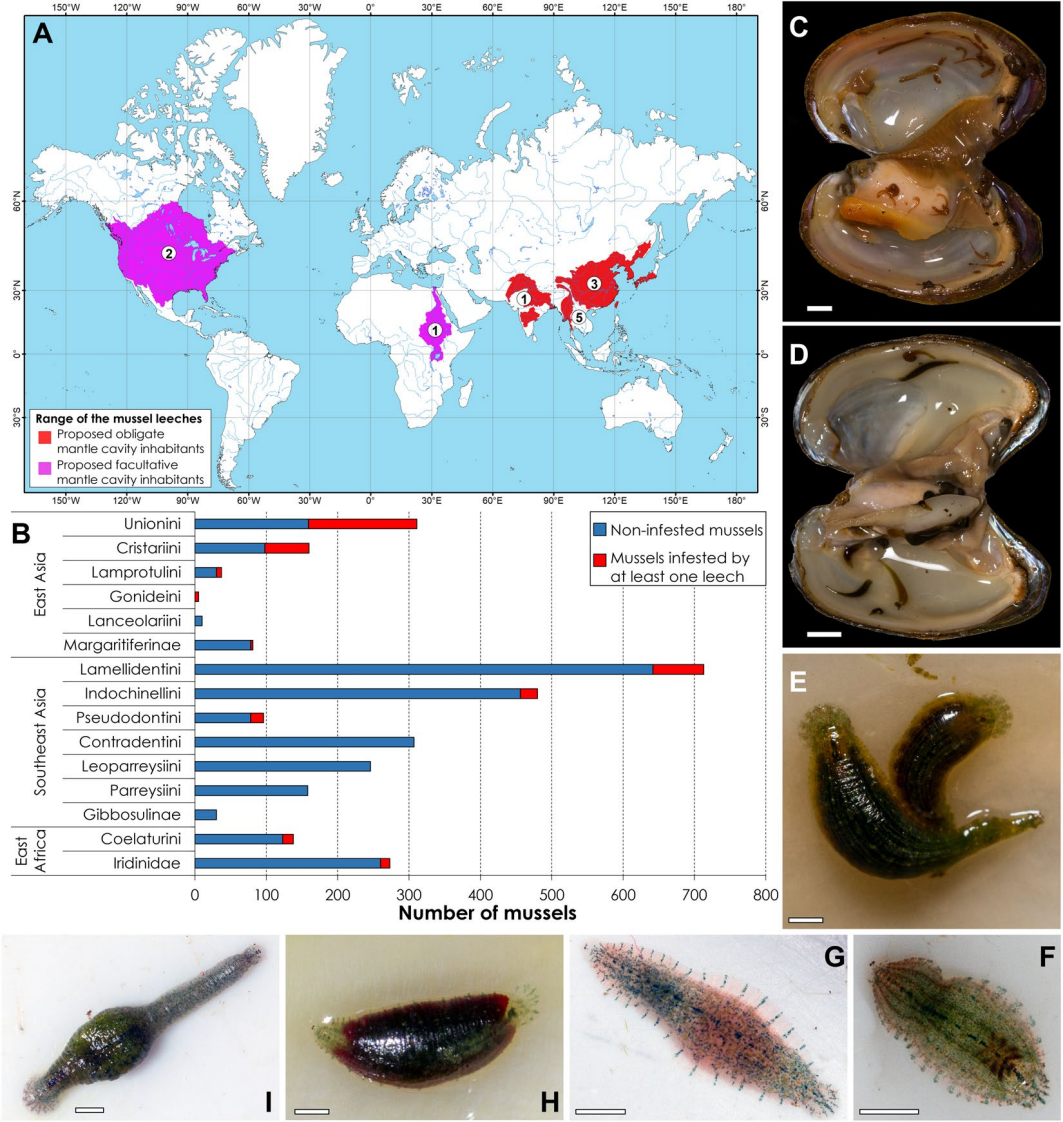
Map of global distribution of leeches associated with freshwater mussels, prevalence of leech infestation of freshwater mussels in the Old World, and living examples of mussel-associated leech species from the mantle cavity of freshwater mussels. (**A**) Map of global distribution of mussel-associated leeches (species richness is given in open circles). North America: *Placobdella montifera* and *P*. *parasitica* [GBIF, https://www.gbif.org]; Africa, Nile Basin: *Batracobdelloides tricarinatus* [this study and Elkhodary *et al*.^27^]; South Asia (India and Nepal): *Batracobdelloides reticulata* [Chandra^25^ and Nesemann *et al*.^79^]; Southeast Asia (Myanmar): *Hemiclepsis myanmariana* **sp**. **nov**., *Batracobdelloides conchophylus* **sp**. **nov**., *B*. *hlaingbweensis* **sp**. **nov**., *B*. *indochinensis* **sp**. **nov**., and *B*. *yaukthwa* **sp**. **nov**. [this study]; East Asia (Russian Far East, Korea, Japan, and eastern China): *Hemiclepsis kasmiana* Oka, 1910 **comb**. **rev**., *H*. *khankiana* **sp**. **nov**., and *Batracobdelloides koreanus* **sp**. **nov**. [this study and Bolotov *et al*.^21^]. The map was created using ESRI ArcGIS 10 software (https://www.esri.com/arcgis); the topographic base of the map was created with Natural Earth Free Vector and Raster Map Data (https://www.naturalearthdata.com) and HydroSHEDS (https://www.hydrosheds.org) (Map: Mikhail Yu. Gofarov). (**B**) Prevalence of leech infestation recovered in samples of freshwater mussels (Unionida: Unionidae, Margaritiferidae, and Iridinidae) from East Asia, Southeast Asia, and East Africa (*N* = 3,045 mussels, primary data: Supplementary Dataset 1). (**C**) Live *Hemiclepsis kasmiana* **comb**. **rev**. [RMBH Hir_0015_1] in the mantle cavity of *Sinanodonta lauta*, Gladkaya River, Russian Far East, 25.x.2016 [scale bar = 10 mm]. (**D**) Live *H*. *myanmariana* **sp**. **nov**. [RMBH Hir_0048_1] in the mantle cavity of *Lamellidens savadiensis*, Nadi Lake, Salween Basin, Myanmar, 23.ii.2018 [scale bar = 10 mm]. (**E**) Live *H*. *myanmariana* **sp**. **nov**. with the crop filled by fish blood meal from the same sample (scale bar = 1 mm). (**F**) Live *B*. *hlaingbweensis* **sp**. **nov**. [RMBH Hir_0207] in the mantle cavity of *Pseudodon salwenianus*, small stream, Hlaingbwe Basin, Myanmar, 17.xi.2018 [scale bar = 1 mm]. (**G**) Live *B*. *indochinensis* **sp**. **nov**. [RMBH Hir_0053_1] in the mantle cavity of *Lamellidens generosus*, Bago - Sittaung Channel, Myanmar, 16.ii.2018 [scale bar = 1 mm]. (**H**) Live *B*. *conchophylus* **sp**. **nov**. [RMBH Hir_0065_1] with the crop filled by fish blood meal in the mantle cavity of *Lamellidens generosus*, ox-bow lake near Taung Gyi village, Lower Sittaung Basin, Myanmar, 20.ii.2018 [scale bar = 1 mm]. (**I**) Live *B*. *tricarinatus* [RMBH Hir_0138] carrying its larvae in the mantle cavity of *Coelatura aegyptiaca*, Lake George, Albert Nile Basin, Uganda, 05.viii.2018 [scale bar = 1 mm]. (Photos: Ilya V. Vikhrev).

**Figure 2:**
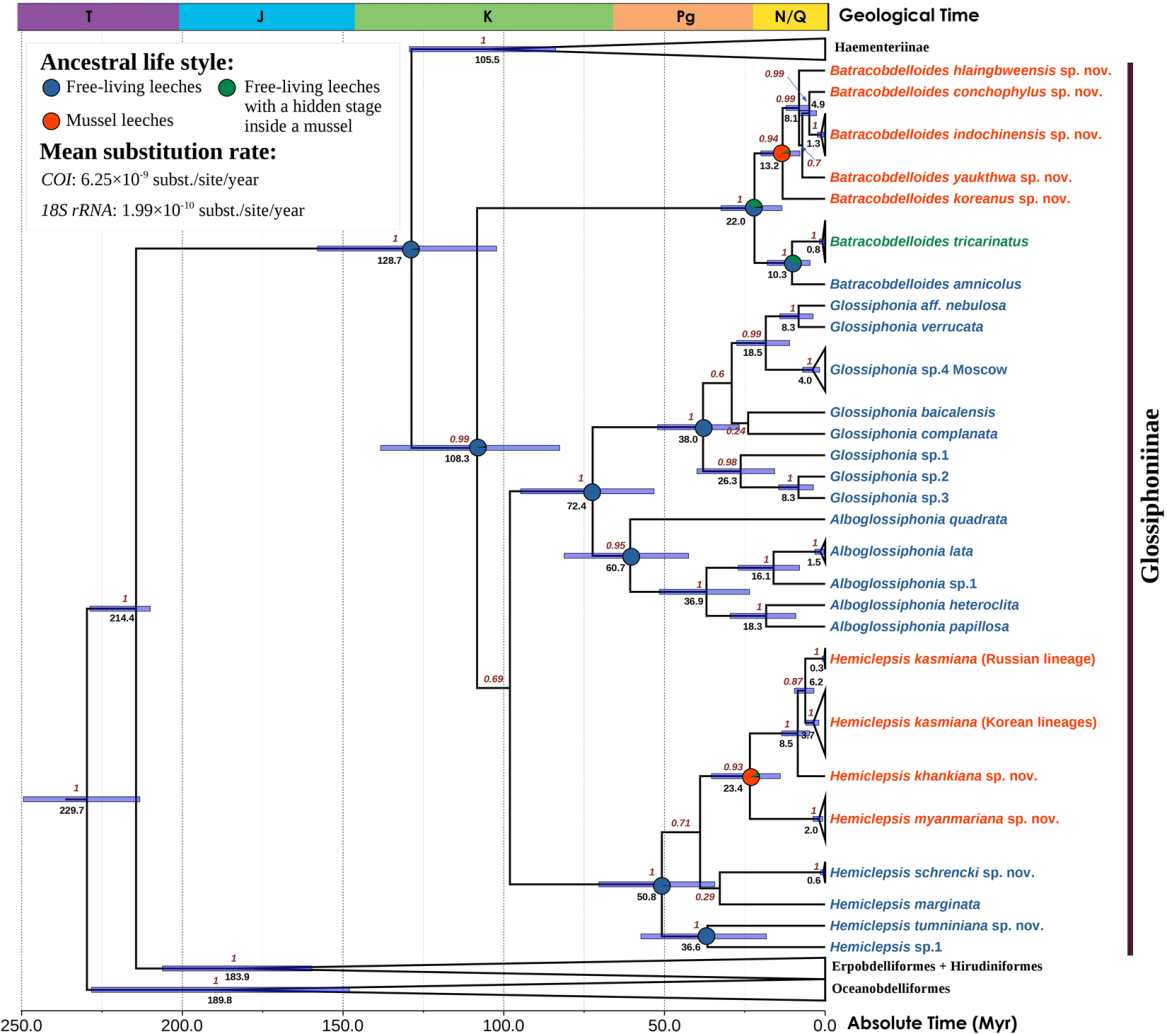
Origin of parasitic leeches from the mantle cavity of freshwater mussels based on two-locus fossil-calibrated phylogeny (four partitions: three codons of *COI*+ *18S rRNA*) calculated under a lognormal relaxed clock model and a Yule process speciation implemented in BEAST 1.10.4. Node bars are 95% HPD of the divergence time. Black numbers near nodes are node ages (Myr). Stratigraphic chart according to the International Commission on Stratigraphy, 2019. Red numbers near nodes are BPP values inferred from BEAST. Divergence times and host reconstructions for weakly supported nodes (BEAST BPP <0.75) are omitted. Host reconstructions are shown for the clades of interest. Proposed obligate inhabitants of the mantle cavity of freshwater mussels are colored red. Free-living leech taxa with a hidden stage inside the mantle cavity of freshwater mussels are colored green. Free-living leech species are colored blue. Outgroup taxa are not shown.

**Figure 3:**
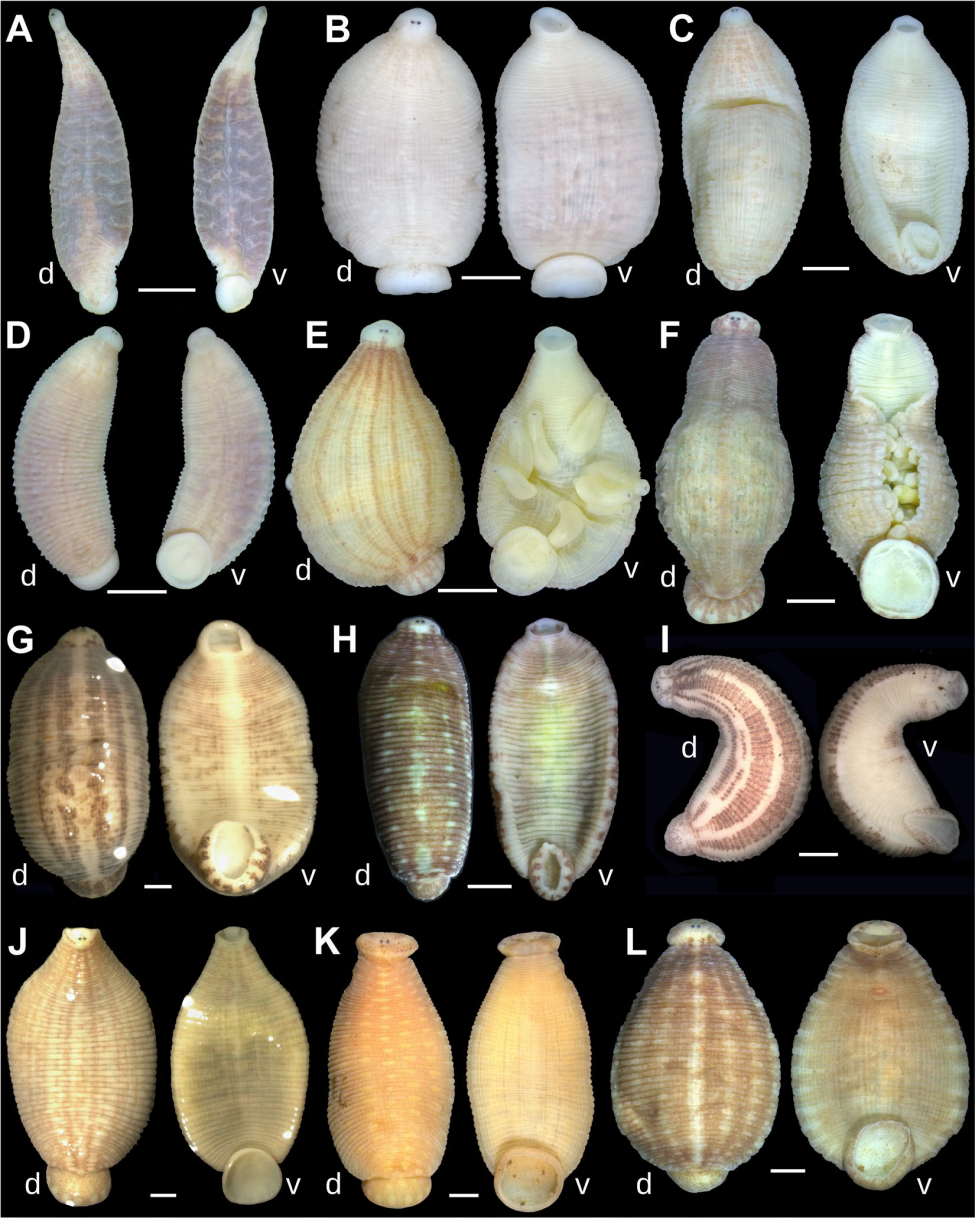
Dorsal (d) and ventral (v) views of the holotypes of new taxa and representative specimens of *Batracobdelloides tricarinatus* and *Hemiclepsis kasmiana* **comb**. **rev**. (**A**) *Batracobdelloides conchophylus* **sp**. **nov**. [holotype RMBH Hir_0065_1-H, Myanmar]. (**B**) *B*. *hlaingbweensis* **sp**. **nov**. [holotype RMBH Hir_0207-H, Myanmar]. (**C**) *B*. *indochinensis* **sp**. **nov**. [holotype RMBH Hir_0066-H, Myanmar]. (**D**) *B*. *yaukthwa* **sp**. **nov**. [holotype RMBH Hir_0060_1-H, Myanmar]. (**E**) *B*. *koreanus* **sp**. **nov**. [holotype RMBH Hir_0116-H, South Korea]. (**F**) *B*. *tricarinatus* [specimen RMBH Hir_0144, Uganda]. (**G**) *Hemiclepsis kasmiana* **comb**. **rev**. [specimen RMBH Hir_0015, Russian Far East]. (**H**) *H*. *kasmiana* **comb**. **rev**. [specimen RMBH Hir_0113_2, South Korea]. (**I**) *H*. *khankiana* **sp**. **nov**. [holotype RMBH Hir_0101-H, Russian Far East]. (**J**) *H*. *myanmariana* **sp**. **nov**. [holotype RMBH Hir_0048_1-H, Myanmar]. (**K**) *H*. *schrencki* **sp**. **nov**. [holotype RMBH Hir_0091_1-H, Russian Far East]. (**L**) *H*. *tumniniana* **sp**. **nov**. [holotype RMBH Hir_0093-H, Russian Far East]. Scale bars = 1 mm. (Photos: Anna L. Klass).

**Figure 4:**
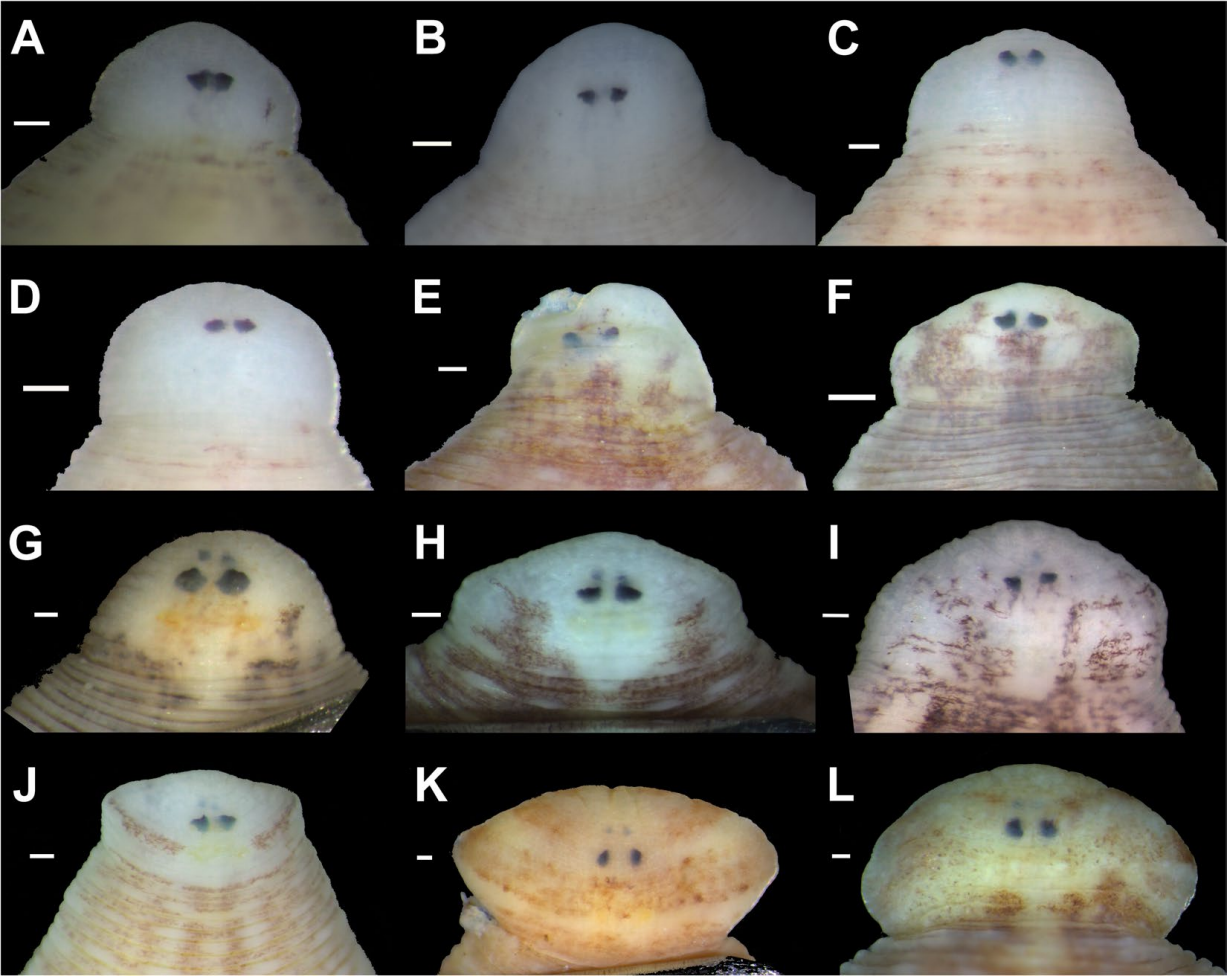
Eye position and shape in the holotypes of new taxa and representative specimens of *Batracobdelloides tricarinatus* and *Hemiclepsis kasmiana* **comb**. **rev**. (**A**) *Batracobdelloides conchophylus* **sp**. **nov**. [paratype RMBH Hir_0055, Myanmar]. (**B**) *B*. *hlaingbweensis* **sp**. **nov**. [holotype RMBH Hir_0207-H, Myanmar]. (**C**) *B*. *indochinensis* **sp**. **nov**. [holotype RMBH Hir_0066-H, Myanmar]. (**D**) *B*. *yaukthwa* **sp**. **nov**. [holotype RMBH Hir_0060_1-H, Myanmar]. (**E**) *B*. *koreanus* **sp**. **nov**. [paratype RMBH Hir_0104, South Korea]. (**F**) *B*. *tricarinatus* [specimen RMBH Hir_0144, Uganda]. (**G**) *Hemiclepsis kasmiana* **comb**. **rev**. [specimen RMBH Hir_0015, Russian Far East]. (**H**) *H*. *kasmiana* **comb**. **rev**. [specimen RMBH Hir_0113_2, South Korea]. (**I**) *H*. *khankiana* **sp**. **nov**. [holotype RMBH Hir_0101-H, Russian Far East]. (**J**) *H*. *myanmariana* **sp**. **nov**. [holotype RMBH Hir_0048_1-H, Myanmar]. (**K**) *H*. *schrencki* **sp**. **nov**. [holotype RMBH Hir_0091_1-H, Russian Far East]. (**L**) *H*. *tumniniana* **sp**. **nov**. [holotype RMBH Hir_0093-H, Russian Far East]. Scale bars = 0.1 mm. (Photos: Anna L. Klass).

**Figure 5:**
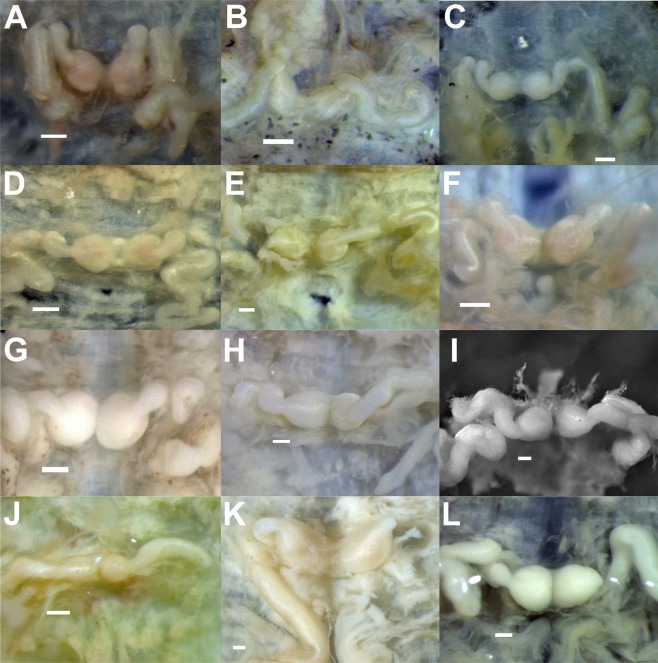
Atrium of the new species, *Batracobdelloides tricarinatus,* and *Hemiclepsis kasmiana* **comb**. **rev**. (dorsal view). (**A**) *Batracobdelloides conchophylus* **sp**. **nov**. [sample RMBH Hir_0065, Myanmar]. (**B**) *B*. *hlaingbweensis* **sp**. **nov**. [sample RMBH Hir_0207, Myanmar]. (**C**) *B*. *indochinensis* **sp**. **nov**. [sample RMBH Hir_0053, Myanmar]. (**D**) *B*. *yaukthwa* **sp**. **nov**. [sample RMBH Hir_0060, Myanmar]. (**E**) *B*. *koreanus* **sp**. **nov**. [paratype RMBH Hir_0104, South Korea]. (**F**) *B*. *tricarinatus* [sample RMBH Hir_0138, Uganda]. (**G**) *Hemiclepsis kasmiana* **comb**. **rev**. [sample RMBH Hir_0015, Russian Far East]. (**H**) *H*. *kasmiana* **comb**. **rev**. [sample RMBH Hir_0116, South Korea]. (**I**) *H*. *khankiana* **sp**. **nov**. [sample RMBH Hir_0101, Russian Far East]. (**J**) *H*. *myanmariana* **sp**. **nov**. [sample RMBH Hir_0048, Myanmar]. (**K**) *H*. *schrencki* **sp**. **nov**. [sample RMBH Hir_0088, Russian Far East]. (**L**) *H*. *tumniniana* **sp**. **nov**. [sample RMBH Hir_0001, Russian Far East]. Scale bars = 0.1 mm. (Photos: Anna L. Klass).

We found that leeches are commonly encountered within the mantle cavity of freshwater mussels in East Asia, Southeast Asia, and East Africa (Fig. 1 and Table 1). In total, the mantle cavities of 3,045 freshwater mussel specimens were examined, with 370 freshwater mussels being infested by 1,334 leeches (except larvae) (Supplementary Dataset 1). The phylogenetic analyses, species delimitation modeling, and morphological assessments reveal that these mussel-associated leeches belong to nine species within two genera, *Hemiclepsis* and *Batracobdelloides* (Glossiphoniidae) (Figs 2–5, Supplementary Figs 1–10, Supplementary Tables 1–4, Supplementary Note 1). The most species-rich assemblage of mussel-associated leeches was recorded in Myanmar. This assemblage includes five species: *Hemiclepsis myanmariana* **sp**. **nov**., *Batracobdelloides conchophylus* **sp**. **nov**., *B*. *hlaingbweensis* **sp**. **nov**., *B*. *indochinensis* **sp**. **nov**., and *B*. *yaukthwa* **sp**. **nov**. (Figs 1–5, Supplementary Figs 7 and 8). In East Asia, three mussel-associated leech species were discovered: *Hemiclepsis kasmiana* **comb**. **rev**. (Russian Far East, Korea, Japan, and also China), *H*. *khankiana* **sp**. **nov**. (Lake Khanka Basin, Russian Far East and China), and *Batracobdelloides koreanus* **sp**. **nov**. (South Korea). The mussel-associated leeches collected in East Africa (Albert Nile Basin, Uganda) belong to a single species largely corresponding to the nominal taxon *Batracobdelloides tricarinatus*. Additionally, two free-living *Hemiclepsis* species new to science, i.e. *H*. *schrencki* **sp**. **nov**. and *H*. *tumniniana* **sp**. **nov**., recorded from East Asia during this study are described here to improve the taxonomy of this genus.

**Table 1:** Mussel-associated leeches of the World (obligate and facultative inhabitants of the mantle cavity of freshwater mussels) with supplement of an overview of free-living species in the genera *Batracobdelloides* Oosthuizen, 1986 and *Hemiclepsis* Vejdovsky, 1884 (complete checklist of these genera is presented as Supplementary Note 1).

Taxa	Life style	Type locality	Distribution	Hosts
**Genus** ***Placobdella*** **Blanchard, 1893**				
*P*. *montifera* Moore, 1906	Facultative inhabitant of the mantle cavity of freshwater mussels	Long Point, Canada^76^	Widespread throughout USA and Canada^13^	Secondary host and shelter (facultative): freshwater mussels *Amblema*, *Cyclonaias*, *Fusconaia*, *Glebula*, *Lampsilis*, *Leptodea*, *Obliquaria*, *Potamilus*, *Pyganodon*, *Utterbackiana* (Unionidae: Ambleminae)^1,10–12^; primary host: freshwater fishes *Acipenser*, *Scaphirhynchus* (Acipenseridae), *Perca* (Percidae), *Lepomis*, *Micropterus* (Centrarchidae), *Lepisosteus* (Lepisosteidae), *Ameiurus* (Ictaluridae), *Moxostoma* (Catostomidae), *Cyprinus* (Cyprinidae), and common musk turtle *Stenotherus odoratus* (Kinosternidae)^10,13^
*P*. *parasitica* (Say, 1824)	Facultative inhabitant of the mantle cavity of freshwater mussels^1^	The lakes of the north-western region [of North America]^77^	North-central and eastern USA and southern Canada^13,78^	Secondary host and shelter (facultative): unspecified freshwater mussels (Unionidae: Ambleminae)^1^; primary host: at least 10 freshwater turtle species^72,78^, occasional records from the tadpoles of *Lithobates pipiens* (Ranidae) and a freshwater fish^72^
**Genus** ***Batracobdelloides*** **Oosthuizen, 1986**				
*B*. *conchophylus* **sp. nov**.	Possible obligate inhabitant of the mantle cavity of freshwater mussels	Myanmar, Lower Sittaung Basin, ox-bow lake near Taung Gyi village, 17.8807°N, 96.8313°E	Myanmar: Sittaung and Haungthayaw river basins	Secondary host and shelter: freshwater mussels *Radiatula* and *Lamellidens* (Unionidae: Parreysiinae); primary host: freshwater fishes *Wallago* (Siluridae)
*B*. *hlaingbweensis* **sp. nov**.	Possible obligate inhabitant of the mantle cavity of freshwater mussels	Myanmar, Hlaingbwe Basin, small stream, 17.0292°N, 97.8099°E	Myanmar: Hlaingbwe River basin	Secondary host and shelter: freshwater mussels *Pseudodon* (Unionidae: Gonideinae); primary host: freshwater fishes *Hemibagrus* (Bagridae)
*B*. *indochinensis* **sp. nov**.	Possible obligate inhabitant of the mantle cavity of freshwater mussels	Myanmar, Salween Basin, fish pond near Demoso, 19.7289°N, 97.1167°E	Myanmar: Bago, Sittaung, Ayeyarwady, and Salween river basins	Secondary host and shelter: freshwater mussels *Lamellidens* and *Trapezidens* (Unionidae: Parreysiinae); primary host: freshwater fishes *Clarias* (Clariidae) and *Oreochromis* (Cichlidae)
*B*. *yaukthwa* **sp. nov**.	Possible obligate inhabitant of the mantle cavity of freshwater mussels	Myanmar, Middle Sittaung Basin, Chain Stream, 17.9769°N, 96.7650°E	Myanmar: Ayeyarwady and Sittaung river basins	Secondary host and shelter: freshwater mussels *Trapezidens* and *Indochinella* (Unionidae: Parreysiinae); primary host: freshwater fishes *Macrognathus* (Mastacembelidae)
*B*. *koreanus* **sp. nov**.	Possible obligate inhabitant of the mantle cavity of freshwater mussels	South Korea, Seomjin River, 35.7010°N, 127.2845°E	South Korea: Seomjin and Mangyeong river basins	Secondary host and shelter: freshwater mussels *Nodularia* (Unionidae: Unioninae); primary host: freshwater fishes *Channa* (Channidae)
*B. *reticulatus* (Kaburaki, 1921)	Possible obligate inhabitant of the mantle cavity of freshwater mussels^23–25^	Jullundur [Jalandhar, Punjab, India]^23^	India and Nepal^23,25,28,79^	Secondary host and shelter: freshwater mussels *Lamellidens* (Unionidae: Parreysiinae)^23,25^; primary host: probably freshwater fishes
*B*. *tricarinatus* (Blanchard, 1897)	Facultative inhabitant of the mantle cavity of freshwater mussels	Tanzania, Mbani (Ugogo), Bubu-Bach^26^	Nile Basin and surrounding endorheic freshwater systems in Africa^26^, records from Israel^26^ are questionable	Secondary host and shelter (facultative): freshwater mussels *Coelatura* (Unionidae: Parreysiinae), *Aspatharia*, *Chambardia*, and *Mutela* (Iridinidae); primary host: freshwater fishes *Synodontis* (Mochokidae)
*B*. *amnicolus* (Moore, 1958) stat. rev.	Free-living species	South Africa, Zululand, Hluhluwe, Engamani River^26^	South Africa^26,80^	Freshwater fishes *Clarias* (Clariidae), *Labeobarbus*, *Carassius* (Cyprinidae), *Oreochromis* (Cichlidae), and unspecified amphibians^26,80^
*B. *moogi* Nesemann & Csänyi, 1995	Free-living species	Hungary, Kisbalaton near the Zala River^28^	Europe: Austria, Hungary, Italy, Lithuania, Montenegro, Poland, and Slovakia^28,70,81^	Pulmonate freshwater snails, chiefly *Planorbarius* (Planorbidae)^70^
**Genus** ***Hemiclepsis*** **Vejdovsky, 1884**				
*H*. *kasmiana* Oka, 1910 comb. rev.	Possible obligate inhabitant of the mantle cavity of freshwater mussels	Hondo (Kasumiga-Ura, Owari, Bizen) [Japan, Honshu: Lake Kasumigaura, Owari Province, and Bizen city]^15^	Widespread through Russian Far East, Korea, Japan, and China^21^	Secondary host and shelter: freshwater mussels *Aculamprotula*, *Buldowskia*, *Cristaria*, *Inversunio*, *Middendorfinaia*, *Nodularia*, *Sinadonta* (Unionidae: Unioninae), *Lamprotula*, *Pronodularia*, *Obovalis* (Unionidae: Gonideinae), *Margaritifera dahurica* (Margaritiferidae)^16,21,82^; primary host: freshwater fishes *Perccottus* (Odontobutidae) and *Silurus* (Siluridae)
*H*. *khankiana* **sp. nov**.	Possible obligate inhabitant of the mantle cavity of freshwater mussels	Russia, Primorye Region, Khanka Lake Basin, Melgunovka River, 44.5804°N, 132.0803°E	Russian Far East: Khanka Lake, Amur Basin	Secondary host and shelter: freshwater mussels *Nodularia* (Unionidae: Unioninae); primary host: freshwater fishes *Rhodeus* (Cyprinidae)
*H*. *myanmariana* **sp. nov**.	Possible obligate inhabitant of the mantle cavity of freshwater mussels	Myanmar, Salween Basin, Nadi Lake, 20.6858°N, 96.9316°E	Myanmar: Ayeyarwady, Sittaung, Bilin, and Salween river basins	Secondary host and shelter: freshwater mussels *Lamellidens* and *Indonaia* (Unionidae: Parreysiinae); primary host: freshwater fishes *Labeo* (Cyprinidae)
*H*. *schrencki* **sp. nov**.	Free-living species	Russia, Primorye Region, Partizanskaya River, 43.0585°N, 133.1540°E	Russian Far East: Partizanskaya and Ussuri river basins	Freshwater fishes *Barbatula* and *Phoxinus* (Nemacheilidae)
*H*. *tumniniana* **sp. nov**.	Free-living species	Russia, Khabarovsk Region, Tumnin River, 50.0001°N, 139.9175°E	Russian Far East: Tumnin Basin	Freshwater fishes *Pungitius* (Gasterosteidae)
*H*. *marginata* (O. F. Müller, 1774)	Free-living species	Unknown, but most likely somewhere in Europe	Widespread through Europe and Siberia from the British Isles^83^ up to the Yenisei Basin and Lake Baikal^84^. Records from East and Southeast Asia^16,19,25,29,85^ refer to other taxa	Chiefly freshwater fishes and amphibian larvae, but also molluscs^29,85^
*H. *japonica* (Oka, 1932)	Free-living species	Japan, Inokasira Pond near Tokyo, Honshu, and one specimen from Sapporo, Hokkaido^86^	Honshu and Hokkaido Islands, Japan^86^	Unknown^29^
*H. *erhaiensis* Yang, 1981	Free-living species	China, Yunnan, Erhai Lake^19,87^	China: Erhai, Dianchi and Chenghai lakes in Yunnan^19,87^	Freshwater fishes^19,87^
*H. *guangdongensis* Tan & Liu, 2001	Free-living species	China, Guangdong Province, Guangzhou (23.02°N, 113.03°E)^88^	China: Lower Pearl River basin^88^	Amboina box turtle *Cuora amboinensis* (Geoemydidae)^88^
*H. *hubeiensis* Yang, 1981	Free-living species	China, Hubei, Huangzhou District, Sanshan Lake^19,87^	China: Sanshan and Ya’er lakes in Yangtze Basin, Hubei^19,87^	Freshwater fishes^19,87^
*H. *asiatica* Moore, 1924 stat. rev.	Free-living species^25,89^	India, Kashmir, Srinagar^89^	India and probably Nepal^25,89^	Freshwater fishes^25,90^
*H. *bhatiai* Baugh, 1960	Free-living species^25,91,92^	India, Bihar, rocky pool ‘Sitkundi’ in Kalipahar, ca. 7 miles SW of Monghyr District^91^	India: Bihar and Jammu and Kashmir^25,91,92^	Unknown^25,29,91^
*H. *charwardamensis* Mandal, 2013 [=*H*. *chharwardamensis* Mandal, 2013 syn. nov.; =*H*. *ischharwardamensis* Mandal, 2013 syn. nov. (our first reviser action on the precedence of simultaneous synonyms)]	Free-living species^93^	India, Jharkhand, Bokaro, Charwardam [=Garga Dam?]^93^	India: known only from the type locality^93^	Unknown, although its association with freshwater molluscs and fishes was speculated by the author of this species^93^ (Supplementary Note 1)
*H. *viridis* Chelladurai, 1934	Free-living species^25,92,94^	India, Trivandrum [Kerala] and Ootacamund [Udagamandalam, Tamil Nadu]^94^	India: Kerala and Tamil Nadu states^25,92^	Frogs^29,94^
*H*. sp.1	Unknown	n/a	South Korea: known from a single specimen	Unknown

*Molecular data for these nominal taxa is still lacking. n/a – not available.

### Divergence time estimation and evolutionary rates

The novel fossil-calibrated phylogeny based on the mitochondrial *cytochrome c oxidase* (*COI*) and the nuclear *small subunit of 18S ribosomal RNA* (*18S rRNA*) gene sequences being combined (Fig. 2 and Supplementary Fig. 3) suggests that the crown group of Hirudinea most likely originated in the mid-Triassic (mean age = 230 Myr, 95% HPD = 213–257 Myr). The origin of the Glossiphoniidae is placed in the Early Cretaceous (mean age = 129 Myr, 95% HPD = 102–158 Myr). The subfamilies Haementeriinae and Glossiphoniinae likely originated almost simultaneously near the Albian – Cenomanian boundary of the mid-Cretaceous (mean age = 105–108 Myr, 95% HPD = 83–138 Myr). The crown group of *Batracobdelloides* most likely had an Early Miocene origin (mean age = 22 Myr, 95% HPD = 13–32 Myr), with the Asian *Batracobdelloides* mussel-associated leech clade having originated in the mid-Miocene (mean age = 13 Myr, 95% HPD = 8–20 Myr). The radiation of *Batracobdelloides* taxa from Myanmar started in the Late Miocene (mean age = 8 Myr, 95% HPD = 5–17 Myr). The most recent common ancestor (MRCA) of *Hemiclepsis* most likely originated in the Early Eocene (mean age = 51 Myr, 95% HPD = 34–70 Myr), while the origin of Asian *Hemiclepsis* mussel-associated leech clade is placed near the Oligocene – Miocene boundary (mean age = 23 Myr, 95% HPD = 14–35 Myr). Our fossil-calibrated BEAST reconstruction indicates that the Hirudinea can be considered a group with slow substitution rates as follows: (1) mean *COI* rate = 6.25 × 10^−9^ subst./site/year (95% HPD 5.06 × 10^−9^–7.53 × 10^−9^ subst./site/year), and (2) mean *18S rRNA* rate = 1.99 × 10^−10^ subst./site/year (95% HPD 1.60 × 10^−10^–2.39 × 10^−10^ subst./site/year).

### Ancestral area reconstruction

Our ancestral area reconstruction combined from three different modeling approaches (S-DIVA + DEC + S-DEC) suggests that the crown group of the Glossiphoniidae had a continuous range throughout East Asia and North America (probability = 99.9%) (Supplementary Table 5 and Supplementary Fig. 5). The ancestral range of the subfamily Haementeriinae most likely crossed North America and South America with a subsequent vicariance event (probability = 86.7%), while the subfamily Glossiphoniinae originated in East Asia with subsequent dispersal events (probability = 98.5%). The genus *Batracobdelloides* most likely spread from East Asia to Africa with a subsequent vicariance event (probability = 96.6%). The MRCA of Asian *Batracobdelloides* mussel-associated leeches most likely had a broad ancestral range in East and Southeast Asia with a subsequent vicariance event associated with the separation of the Asian freshwater drainages from each other (probability = 99.7%). The genus *Hemiclepsis*, in its turn, originated in East Asia following by an intra-area radiation (probability = 75.8%). Finally, the Asian *Hemiclepsis* mussel-associated leeches have had a broad ancestral range throughout East and Southeast Asia with a subsequent vicariance event (probability = 99.7%). The patterns outlined above were also supported by each modeling approach separately (Supplementary Table 5 and Supplementary Fig. 5).

### Ancestral life style reconstruction

The MRCA of *Hemiclepsis* was most likely a free-living species (probability = 99.6%), while the MRCA of the clade with mussel-associated leech species belonging to this genus was most likely an obligate mussel inhabitant as its recent descendants (probability = 90.8%) (Fig. 2 and Supplementary Fig. 6). The ancestral reconstruction for *Batracobdelloides* suggests that the MRCA of this genus was a free-living species (probability = 60.1%) rather than a free-living species with a facultative hidden stage inside the mantle cavity of freshwater mussels (probability = 37.4%). Reconstruction of the MRCA of a clade containing two African members of *Batracobdelloides* returns similar probability patterns (63.6 and 36.4%, respectively). In contrast, the MRCA of the clade of Asian *Batracobdelloides* mussel-associated leeches within this genus was most likely a mussel inhabitant (probability = 94.2%).

### Life cycles and feeding of the mussel leeches

**Figure 6:**
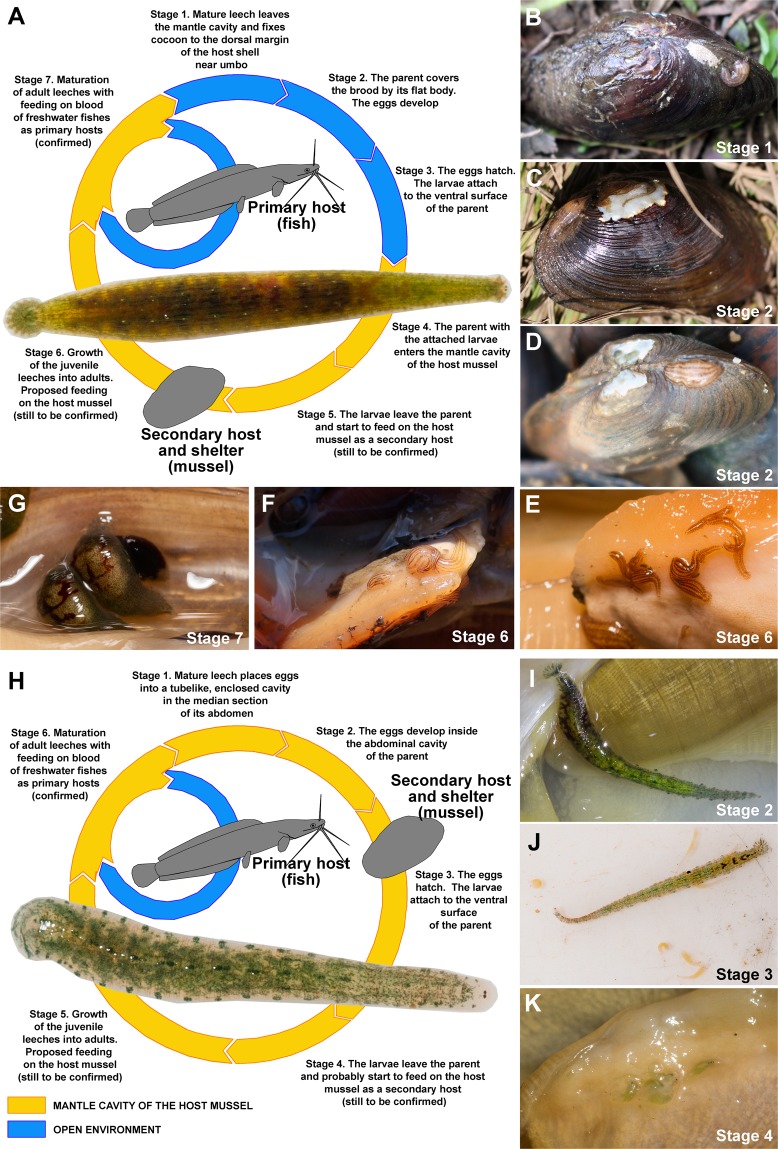
Proposed life cycles of mussel leeches. (**A**) General scheme of the life cycle of *Hemiclepsis* mussel leeches (*H*. *kasmiana* **comb**. **rev**., *H*. *khankiana* **sp**. **nov**., and *H*. *myanmariana* **sp**. **nov**.) (field observations are given in Supplementary Table 6). Leech specimen: *H*. *myanmariana* **sp**. **nov**. [sample RMBH Hir_0048] from the mantle cavity of *Lamellidens savadiensis*, Nadi Lake Salween Basin, Myanmar, 23.ii.2018. (**B**) *H*. *kasmiana* **comb**. **rev**. near its cocoon on the dorsal margin of *Middendorffinaia mongolica* shell, Gladkaya River, Russian Far East, 28.vi.2018. (**C**) *H*. *kasmiana* **comb**. **rev**. covers its brood on the dorsal margin of *Middendorffinaia mongolica* shell, Gladkaya River, Russian Far East, 28.vi.2018. (**D**) *H*. *khankiana* **sp**. **nov**. covers its brood on the dorsal margin of *Nodularia douglasiae* shell, Melgunovka River, Khanka Lake Basin, Russian Far East, 01.vii.2018. (**E**) Juvenile and adult individuals of *H*. *kasmiana* **comb**. **rev**. [sample RMBH Hir_0015_4] in the mantle cavity of *Middendorffinaia mongolica*, Gladkaya River, Russian Far East, 25.x.2016. (**F**) Juvenile and adult individuals of *H*. *khankiana* **sp**. **nov**. [sample RMBH Hir_0101] in the mantle cavity of *Nodularia douglasiae*, Melgunovka River, Khanka Lake Basin, Russian Far East, 01.vii.2018. (**G**) Mature individuals of *H*. *myanmariana* **sp**. **nov**. [sample RMBH Hir_0059] with developing eggs (their crops are filled by fish blood meal) in the mantle cavity of *Lamellidens savadiensis*, main channel of the Ayeyarwady River, Myanmar, 04.iii.2018. (**H**) General scheme of the life cycle of *Batracobdelloides* mussel leeches (*B*. *conchophylus* **sp**. **nov**., *B*. *hlaingbweensis* **sp**. **nov**., *B*. *indochinensis* **sp**. **nov**., *B*. *yaukthwa* **sp**. **nov**., and probably *B*. *koreanus* **sp**. **nov**. and *B*. *reticulatus*) (field observations are given in Supplementary Table 7). Leech specimen: *B*. *indochinensis* **sp**. **nov**. [sample RMBH Hir_0053_1] from the mantle cavity of *Lamellidens generosus*, Bago - Sittaung channel, Myanmar, 16.ii.2018. (**I**) Mature individual of *B*. *conchophylus* **sp**. **nov**. [sample RMBH Hir_0065_1] carrying eggs in the mantle cavity of *Lamellidens generosus*, ox-bow lake near Taung Gyi village, Lower Sittaung Basin, Myanmar, 20.ii.2018. (**J**) Mature individual of *B*. *hlaingbweensis* **sp**. **nov**. with larvae attached to and partly escaped from its abdomen [sample RMBH Hir_0207], from the mantle cavity of *Pseudodon salwenianus*, small stream Hlaingbwe Basin, Myanmar, 29.xi.2018. (**K**) Larvae of *B*. *yaukthwa* **sp**. **nov**. [sample RMBH Hir_0062] on the foot of *Indochinella pugio viridissima*, Chain Stream, Middle Sittaung Basin, Myanmar, 20.ii.2018. (Photos: Ilya V. Vikhrev [**A**,**E**,**G**,**H**–**K**] and Alexander V. Kondakov [**B**–**D**,**F**]; Graphics: Ivan N. Bolotov [A,H]).

Seven stages were observed in the life cycle of *Hemiclepsis* mussel-associated leeches in the field, most of which occur within the mantle cavity of a host mussel (Fig. 6 and Supplementary Table 6). The mature leech leaves the mantle cavity, fixes the egg cluster to the dorsal margin of the host shell near the umbo, and covers the brood by its flat body. After hatching, the larvae attach to the abdomen of the parent, which enters the mantle cavity of the host mussel, with a subsequent internal development of the juvenile leeches into adults. In contrast, only six stages were observed in the life cycle of *Batracobdelloides* mussel-associated leeches (Fig. 6 and Supplementary Table 7), because they do not need to leave the host mussel for brooding. These leeches place the egg cluster into a tube-like, enclosed cavity in the median section of their abdomen.

The multiple records of abundant larvae and young leeches inside the mantle cavity indicate that the mussel leech species probably use freshwater mussels as their secondary host (Supplementary Tables 6 and 7), while direct evidence of this suggestion (e.g. molecular data for the digestive system of the immature leeches) is still to be collected. In contrast, the digestive system (crop) of dissected mature specimens of *Batracobdelloides* and *Hemiclepsis* mussel-associated taxa is filled with a dark red or brown blood-like substance that does not resemble the translucent mussel body fluids (Supplementary Fig. 10). The *COI* sequences of the crop content of mature leeches reveal that they feed on blood of freshwater fishes that can be considered the primary hosts (Table 1 and Supplementary Table 8). Both groups of the mussel leeches disperse at the adult stage when they leave their mussel hosts feed on fish blood.

### Host range and abundance of mussel-associated leeches

Two leech species associated with freshwater mussels, i.e. *Hemiclepsis kasmiana* **comb**. **rev**. and *Batracobdelloides tricarinatus*, could be considered secondary host generalists, each of which inhabits mussel species from different families (Table 1). In contrast, other mussel leech species appear to be associated with one or a few genera of freshwater mussels belonging to a single subfamily or even tribe (Table 1). The proportion of mussels infested by at least one leech (Leech Infestation Prevalence index, *LIP*; Eq. 1) was significantly influenced by the taxonomic affinities of the host mussels at the tribe level in Southeast Asia (Kruskal-Wallis test: *H* (6; *N* = 144) = 29.83; *P* < 0.0001) and East Asia (Kruskal-Wallis test: *H* (5; *N* = 41) = 12.80; *P* = 0.0253) (Supplementary Dataset 1). The same pattern was found using the mean intensity of leech infestation (*ILI*, leeches per mussel [l.p.m.]; Eq. 2) in Southeast Asia (Kruskal-Wallis test: *H* (6; *N* = 144) = 30.02; *P* < 0.0001) and East Asia (Kruskal-Wallis test: *H* (5; *N* = 41) = 12.58; *P* = 0.0276) (Supplementary Dataset 1). In Southeast Asia (Myanmar), mussel-associated leeches infest Lamellidentini, Indochinellini, and Pseudodontini mussels, but they were never recorded in members of the Leoparreysiini, Parreysiini, Contradentini, and Margaritiferidae (Gibbosulinae) (Fig. 1B). In East Asia, mussel-associated leeches prefer the Unionini and Cristariini mussels, a few examples were also collected from the Lamprotulini, Gonideini and Margaritiferidae (Margaritiferinae), while the Lanceolariini do not seem to be infested.

The highest mean *LIP* was found in East Asia (*LIP* ± s.e.m. = 60.0 ± 6.2%; *N* = 28), while this parameter was lower in Southeast Asia (27.8 ± 4.4%; *N* = 27) and East Africa (8.3 ± 3.3%; *N* = 4) (Kruskal-Wallis test: *H* (2; *N* = 59) = 19.04; *P* = 0.0001) (Supplementary Table 9). The mean intensity of leech infestation was also higher in East Asia (*ILI* ± s.e.m. = 3.3 ± 0.7 l.p.m.; *N* = 28) compared with those in Southeast Asia (0.68 ± 0.20 l.p.m.; *N* = 27) and East Africa (0.10 ± 0.04 l.p.m.; *N* = 4) (Kruskal-Wallis test: *H* (2; *N* = 59) = 18.83; *P* = 0.0001) (Supplementary Table 9). High or moderate levels of the mean *LIP* were observed in several river drainages such as the Amur, Gladkaya, and Razdolnaya (Russia), Seomjin and Geum (South Korea), Hyakuken (Japan), Sittaung, Salween, Ayeyarwady, and Hlaingbwe (Myanmar), and the Albert Nile (Uganda) (Supplementary Fig. 11).

### Taxonomy

Here, we introduce  nine leech species new to science based on diagnostic morphological and molecular characters. A complete morphological description of every novel species is given in Supplementary Table 4. A checklist of *Batracobdelloides* and *Hemiclepsis* species is presented in Supplementary Note 1. A key to the mussel-associated leeches of the Old World based on external morphological characters of ethanol-preserved specimens is provided in Supplementary Note 2.

**Suborder Glossiphoniiformes Tessler & de Carle, 2018**

**Family Glossiphoniidae Vaillant, 1890**

**Subfamily Glossiphoniinae Vaillant, 1890**

**Genus** ***Batracobdelloides*** **Oosthuizen, 1986**.

Type species: *Helobdella tricarinata* Blanchard, 1897 (by original designation).

#### Distribution

East, Southeast and South Asia, with a small radiation in Africa (two species) and Europe (one species) (Table 1).

#### Comments

It was thought that *Batracobdelloides* was a small genus with only three species^18,26,28^. However, this genus contains at least nine species, five of which are described here as new to science (Table 1 and Supplementary Note 1).

***Batracobdelloides conchophylus*** **Bolotov**, **Klass**, **Bespalaya**, **Konopleva**, **Kondakov & Vikhrev sp**. **nov**.

Figures 3A, 4A, 5A, Table 2, Supplementary Table 4, Supplementary Figs 7D, 10A.

**Table 2:** Voucher numbers, reference DNA sequences and measurements for the type series of new leech species.

Species	Status of specimen	Sample ID*	*COI* acc. no.	*18S rRNA* acc. no.	Measurements (mm)**			
					BL	BW	AW	PW
*Batracobdelloides conchophylus* **sp. nov**.	Holotype	Hir_0065_1-H	n/a	n/a	5.44	1.45	0.28	0.68
	Paratype	Hir_0065_1	MN295408	MN312185	10.30	2.78	0.58	1.32
	Paratype	Hir_0055	n/a	n/a	5.01	2.86	0.76	1.16
*B*. *hlaingbweensis* **sp. nov**.	Holotype	Hir_0207-H	n/a	n/a	3.82	2.13	0.62	1.01
	Paratype	Hir_0207	n/a	n/a	4.24	2.19	0.74	0.98
	Paratype	Hir_0209	MN295455	n/a	5.70	2.90	0.80	1.25
	Paratype	Hir_0214	MN295457	MN595225	4.04	2.33	0.59	1.10
	Paratype	Hir_0215	MN295458	n/a	5.34	3.42	0.65	1.61
*B*. *indochinensis* **sp. nov**.	Holotype	Hir_0066-H	n/a	n/a	5.89	2.71	0.71	0.91
	Paratype	Hir_0066	MN295409	MN312186	4.00	2.10	0.58	0.88
	Paratype	Hir_0053	n/a	n/a	4.56	2.29	0.76	1.03
	Paratype	Hir_0053_1	MN295399	n/a	6.76	3.44	1.07	1.63
	Paratype	Hir_0056_1	n/a	n/a	4.95	1.34	0.46	0.98
	Paratype	Hir_0056_1	MN295402	n/a	4.11	2.45	0.62	0.97
*B*. *yaukthwa* **sp. nov**.	Holotype	Hir_0060_1-H	n/a	n/a	4.70	1.53	0.50	0.94
	Paratype	Hir_0060_1	n/a	n/a	6.28	2.30	0.56	0.87
	Paratype	Hir_0060_1	n/a	n/a	4.42	1.88	0.48	0.81
	Paratype	Hir_0060_1	MN295406	MN312184	10.40	4.11	0.86	1.85
	Paratype	Hir_0062	n/a	n/a	5.64	1.47	0.56	1.08
	Paratype	Hir_0062	MN295407	n/a	4.46	1.81	0.54	0.99
*B*. *koreanus* **sp. nov**.	Holotype	Hir_0116_2-H	n/a	n/a	4.58	2.79	0.73	1.12
	Paratype	Hir_0104	MN295424	MN312194	8.15	4.37	0.78	1.40
*Hemiclepsis khankiana* **sp. nov**.	Holotype	Hir_0101-H	MN295420	MN312192	7.52	2.83	1.09	1.61
	Paratype	Hir_0101	n/a	n/a	11.80	3.11	1.11	1.63
	Paratype	Hir_0101	n/a	n/a	9.11	2.18	1.13	1.45
	Paratype	Hir_0101	n/a	n/a	9.94	2.34	1.03	1.76
	Paratype	Hir_0018	n/a	n/a	5.15	2.00	0.75	1.57
	Paratype	Hir_0123_1	n/a	n/a	9.61	2.54	1.08	1.57
*H*. *myanmariana* **sp. nov**.	Holotype	Hir_0048_1-H	MN295394	MN312180	11.30	5.72	1.41	2.46
	Paratype	Hir_0048_1	n/a	n/a	7.94	3.31	0.82	1.80
	Paratype	Hir_0048-1	n/a	n/a	7.09	3.51	0.98	2.18
	Paratype	Hir_0048_1	n/a	n/a	8.60	3.99	0.95	2.08
	Paratype	Hir_0210	n/a	n/a	4.98	4.68	0.89	1.20
	Paratype	Hir_0211	MN295456	n/a	5.21	3.55	0.83	1.48
*H*. *schrencki* **sp. nov**.	Holotype	Hir_0091_1-H	MN295416	MN312190	9.70	4.30	2.20	2.48
	Paratype	Hir_0088_1	MN295415	MN312189	13.30	9.58	2.84	3.40
*H*. *tumniniana* **sp. nov**.	Holotype	Hir_0093-H	MN295417	MN312191	7.76	5.20	1.78	2.09
	Paratype	Hir_0001	MN295371	MN312166	7.68	3.94	1.31	1.55
	Paratype	Hir_0001	n/a	n/a	4.98	3.31	1.32	1.58
	Paratype	Hir_0001	n/a	n/a	5.52	1.90	1.29	1.49
	Paratype	Hir_0014	n/a	n/a	5.02	2.79	1.15	1.71
	Paratype	Hir_0235	n/a	n/a	8.74	3.71	1.85	2.33
	Paratype	Hir_0235	n/a	n/a	10.20	4.18	1.85	2.31

*Type series are deposited in the RMBH – Russian Museum of Biodiversity Hotspots, Federal Center for Integrated Arctic Research, Russian Academy of Sciences, Arkhangelsk, Russia. **Measurements of leech specimens (mm): BL – body length, BW – body width, AW – width of anterior sucker, and PW – width of posterior sucker.

Holotype RMBH **Hir_0065_1-H**, MYANMAR: Lower Sittaung Basin, ox-bow lake near Taung Gyi village, 17.8807°N, 96.8313°E, from the mantle cavity of *Lamellidens generosus* [RMBH biv0393], 20.ii.2018, Bolotov, Vikhrev, and Nyein Chan leg.

#### Paratypes

MYANMAR: type locality, same host, date and collectors, one specimen [RMBH **Hir_0065_1**]; Sittaung River near Kanna village, 19.4857°N, 96.2750°E, from the mantle cavity of *Radiatula mouhoti* [RMBH biv0409], 28.ii.2018, one specimen [RMBH **Hir_0055**], Bolotov, Vikhrev, and Nyein Chan leg.

#### Etymology

The name of this species reflects its preference in using freshwater mussels as hosts.

#### Morphological diagnosis

Small leech, which could be distinguished from other congeners by a combination of the following characters: dorsum white, with seven rows of unclear light brown spots in the anterior third of the animal, and with three longitudinal rows of conical tubercles with rounded apex, eyes joined into one large angulate-ovate spot (Supplementary Table 4).

#### Molecular diagnosis

The new species differs from other congeners by nine fixed nucleotide substitutions in the *COI* gene fragment (Supplementary Table 3). Genetically, it is most closely related to *Batracobdelloides indochinensis* **sp**. **nov**. (mean *COI* p-distance = 4.1%).

#### Life style

A mussel leech species that seems to be a host mussel specialist (Table 1).

#### Distribution

Sittaung and Haungthayaw basins, Myanmar.

***Batracobdelloides hlaingbweensis*** **Bolotov**, **Klass**, **Bespalaya**, **Konopleva**, **Kondakov & Vikhrev sp**. **nov**.

Figures 3B, 4B, 5B, Table 2, Supplementary Table 4, Supplementary Figs 7A, 10B.

Holotype RMBH **Hir_0207-H**, MYANMAR: Hlaingbwe Basin, small stream, 17.0292°N, 97.8099°E, from the mantle cavity of *Pseudodon salwenianus* [RMBH biv0639], 17.xi.2018, Than Win leg.

#### Paratypes

MYANMAR: type locality, same host, date and collector, one specimen [RMBH **Hir_0207**], from the mantle cavity of *Pseudodon* sp.1 [RMBH biv0638], one specimen [RMBH **Hir_0209**]; Myanmar, Hlaingbwe Basin, small stream, 17.0483°N, 97.8194°E, from the mantle cavity of *Pseudodon salwenianus* [RMBH biv0674], 14.xi.2018, 2 specimens [RMBH **Hir_0214**, **Hir_0215**], Than Win leg.

#### Etymology

The name of this species is derived from the name of its type locality, the Hlaingbwe River basin.

#### Morphological diagnosis

Small leech, which could be distinguished from other congeners by a combination of the following characters: dorsum greenish or white, without clear markings, with one central row of triangular, flattened tubercles with rounded apex, and similar lateral tubercles being broadly scattered (Supplementary Table 4).

#### Molecular diagnosis

The new species differs from other congeners by five fixed nucleotide substitutions in the *COI* gene fragment (Supplementary Table 3). Genetically, it is most closely related to *Batracobdelloides yaukthwa* **sp**. **nov**. (mean *COI* p-distance = 3.8%).

#### Life style

A mussel leech species that seems to be a host mussel specialist (Table 1).

#### Distribution

Hlaingbwe Basin, Myanmar.

***Batracobdelloides indochinensis*** **Bolotov**, **Klass**, **Bespalaya**, **Konopleva**, **Kondakov & Vikhrev sp**. **nov**.

Figures 3C, 4C, 5C, Table 2, Supplementary Table 4, Supplementary Figs 7B, 10C.

Holotype RMBH **Hir_0066-H**, MYANMAR: Salween Basin, fish pond near Demoso, 19.7289°N, 97.1167°E, from the mantle cavity of *Lamellidens ferrugineus* [RMBH biv0404], 25.ii.2018, Bolotov, Vikhrev, and Nyein Chan leg.

#### Paratypes

MYANMAR: type locality, same host, date and collectors, one specimen [RMBH **Hir_0066**]; Bago-Sittaung Channel, 17.5818°N, 96.7733°E, from the mantle cavity of *Lamellidens generosus* [RMBH biv0376], 16.ii.2018, 2 specimens [RMBH **Hir_0053_1**, **Hir_0053**], Middle Sittaung Basin, Mone Ding Dam outlet, 20.8099°N, 95.7242°E, from the mantle cavity of *Lamellidens savadiensis* [RMBH biv0415], 01.iii.2018, 2 specimens [RMBH **Hir_0056_1**], Bolotov, Vikhrev, and Nyein Chan leg.

#### Etymology

The name of this species is derived from the name of the Indochina Peninsula.

#### Morphological diagnosis

Small leech, which could be distinguished from other congeners by a combination of the following characters: dorsum white or yellowish (sometimes with diffuse brownish dots scattered throughout the anterior third of the animal), with three longitudinal rows of conical tubercles with rounded apex; posterior sucker without brown marking pattern or with a few unclear light brown spots (Supplementary Table 4).

#### Molecular diagnosis

The new species differs from other congeners by seven fixed nucleotide substitutions in the *COI* gene fragment (Supplementary Table 3). Genetically, it is most closely related to *Batracobdelloides conchophylus* **sp**. **nov**. (mean *COI* p-distance = 4.1%).

#### Life style

A mussel leech species that seems to be a host mussel specialist (Table 1).

#### Distribution

Bago, Sittaung, Ayeyarwady, and Salween basins, Myanmar.

***Batracobdelloides yaukthwa*** **Bolotov**, **Klass**, **Bespalaya**, **Konopleva**, **Kondakov & Vikhrev sp**. **nov**.

Figures 3D, 4D, 5D, Table 2, Supplementary Table 4, Supplementary Figs 7C, 10D.

Holotype RMBH **Hir_0060_1-H**, MYANMAR: Middle Sittaung Basin, Chain Stream, 17.9769°N, 96.7650°E, from the mantle cavity of *Trapezidens angustior* [RMBH biv0394], 20.ii.2018, Bolotov, Vikhrev, and Nyein Chan leg.

#### Paratypes

MYANMAR: type locality, same host, date and collectors, 3 specimens [RMBH **Hir_0060_1**], type locality, same date and collectors, from the mantle cavity of *Indochinella pugio viridissima* [RMBH biv0395], 2 specimens [RMBH **Hir_0062**].

#### Etymology

The name of this species means “freshwater bivalve” (*yaukthwa*) in Burmese reflecting its preference to use freshwater mussels as hosts.

#### Morphological diagnosis

Small leech, which could be distinguished from other congeners by a combination of the following characters: dorsum reddish or brownish, with one central row of spike-like tubercles and separate spike-like tubercles being scattered laterally (Supplementary Table 4).

#### Molecular diagnosis

The new species differs from other congeners by nine fixed nucleotide substitutions in the *COI* gene fragment (Supplementary Table 3). Genetically, it is most closely related to *Batracobdelloides hlaingbweensis* **sp**. **nov**. (mean *COI* p-distance = 3.8%).

#### Life style

A mussel leech species that seems to be a host mussel specialist (Table 1).

#### Distribution

Ayeyarwady and Sittaung basins, Myanmar.

***Batracobdelloides koreanus*** **Bolotov**, **Klass**, **Bespalaya**, **Konopleva**, **Kondakov & Vikhrev sp**. **nov**.

Figures 3E, 4E, 5E, Table 2, Supplementary Table 4.

Holotype RMBH **Hir_0116_1-H**, SOUTH KOREA: Seomjin River, 35.7010°N, 127.2845°E, from the mantle cavity of *Nodularia sinuata* [RMBH biv0517], 10.vii.2018, Bogan, Bolotov, Kim, Kondakov, Lopes-Lima, Lee, and Vikhrev leg.

#### Paratype

SOUTH KOREA: Mangyeong River, irrigation channel, 35.9165°N, 127.7135°E, from the mantle cavity of *Nodularia* sp., 11.vii.2018, one specimen [RMBH **Hir_0104**], Bogan, Bolotov, Kim, Kondakov, Lopes-Lima, Lee, and Vikhrev leg.

#### Etymology

The name of this species is derived from the East Asian region, in which it is distributed.

#### Morphological diagnosis

Small leech, which could be distinguished from other congeners by a combination of the following characters: dorsum light yellow, with seven longitudinal brown stripes and three rows of weakly developed, almost invisible, rounded tubercles; posterior sucker with radial brownish bands (Supplementary Table 4).

#### Molecular diagnosis

The new species differs from other congeners by 10 fixed nucleotide substitutions in the *COI* gene fragment (Supplementary Table 3) and one fixed nucleotide substitution in the *18S* gene fragment [154T]. Genetically, it is most closely related to *Batracobdelloides hlaingbweensis* **sp**. **nov**. (mean *COI* p-distance = 5.1%).

#### Life style

A mussel leech species that seems to be a host mussel specialist (Table 1).

#### Distribution

Seomjin and Mangyeong basins, South Korea.

**Genus** ***Hemiclepsis*** **Vejdovsky, 1884**

Type species: *Hirudo marginata* O. F. Müller, 1774 (by subsequent designation).

#### Distribution

East, Southeast and South Asia, with one species in Siberia and Europe (Table 1).

#### Comments

This genus contains at least 15 species, 4 of which are described here as new to science (Table 1 and Supplementary Note 1). *Batracobdella kasmiana* was described as a *Hemiclepsis* species^15,17^, but later it was moved to *Batracobdella* without any explanation^29,30^. Recently, it was assumed that this species is a member of its original genus based chiefly on its external characters^21^. Here, we propose *Hemiclepsis kasmiana* **comb**. **rev**. as a final solution based on our phylogenies (Fig. 2, Supplementary Figs 1–4).

***Hemiclepsis khankiana*** **Bolotov**, **Klass**, **Bespalaya**, **Konopleva**, **Kondakov & Vikhrev sp**. **nov**.

Figures 3I, 4I, 5I, Table 2, Supplementary Table 4, Supplementary Fig. 8A.

Holotype RMBH **Hir_0101-H**, RUSSIA: Primorye Region, Khanka Lake Basin, Melgunovka River, 44.5804°N, 132.0803°E, from the mantle cavity of *Nodularia douglasiae*, 01.vii.2018, Bolotov, Vikhrev, and Kondakov leg.

#### Paratypes

RUSSIA: type locality, same host, date and collectors, 3 specimens [RMBH **Hir_0101**]; Melgunovka River, 44.5939°N, 132.1818°E, from the mantle cavity of *Nodularia douglasiae*, 01.vii.2018, one specimen [RMBH **Hir_0123_2**], Bolotov, Vikhrev, and Kondakov leg.; Spasovka River near Gayvoron village, 44.7565°N, 132.7643°E, 13.viii.2016, from the mantle cavity of *Nodularia douglasiae*, one specimen [RMBH **Hir_0018**], Sayenko leg.

#### Etymology

The name of this species is derived from the Khanka Lake basin, where the type series was collected.

#### Morphological diagnosis

Medium-sized leech, which could be distinguished from other congeners by a combination of the following characters: dorsum yellowish or whitish, with six longitudinal broad, smooth brown stripes; posterior sucker without bands, but with dense, diffuse brown dots (Supplementary Table 4).

#### Molecular diagnosis

The new species differs from other congeners by five fixed nucleotide substitutions in the *COI* gene fragment (Supplementary Table 3). Genetically, it is most closely related to *Hemiclepsis kasmiana* **comb**. **rev**. (mean *COI* p-distance = 3.8%).

#### Life style

A mussel leech species that seems to be a host mussel specialist (Table 1).

#### Distribution

Khanka Lake Basin, Russia and probably China.

***Hemiclepsis myanmariana*** **Bolotov**, **Klass**, **Bespalaya**, **Konopleva**, **Kondakov & Vikhrev sp**. **nov**.

Figures 3J, 4J, 5J, Table 2, Supplementary Table 4, Supplementary Figs 8B, 10G.

Holotype RMBH **Hir_0048_1-H**, MYANMAR: Salween Basin, Nadi Lake, 20.6858°N, 96.9316°E, from the mantle cavity of *Lamellidens savadiensis* [RMBH biv0399], 23.ii.2018, Bolotov, Vikhrev, and Nyein Chan leg.

#### Paratypes

MYANMAR: type locality, same host, date and collectors, 3 specimens [RMBH **Hir_0048_1**]; Ayeyarwady Basin, Nga Wun River near Pyay town, 18.8624°N, 95.2822°E, from the mantle cavity of *Lamellidens savadiensis* [RMBH biv0672], 11.xii.2018, 2 specimens [RMBH **Hir_0210**, **Hir_0211**], Bolotov, Vikhrev, Lopes-Lima, Bogan, and Nyein Chan leg.

#### Etymology

The name of this species is derived from the country of Myanmar, where it is distributed.

#### Morphological diagnosis

Small leech, which could be distinguished from other congeners by a combination of the following characters: dorsum yellowish, brownish or whitish, sometimes with unclear longitudinal narrow light brown stripes and rows of light brown dashes; posterior sucker brownish, with large white spots marginally (Supplementary Table 4). Externally, it resembles rather *Batracobdelloides* than *Hemiclepsis* by a small, weakly separated head and weakly developed brown markings on the dorsum.

#### Molecular diagnosis

The new species differs from other congeners by 11 fixed nucleotide substitutions in the *COI* gene fragment and four fixed nucleotide substitutions in the *18S* gene fragment (Supplementary Table 3). Genetically, it is most closely related to *Hemiclepsis khankiana* **sp**. **nov**. (mean *COI* p-distance = 7.1%).

#### Life style

A mussel leech species that seems to be a host mussel specialist (Table 1).

#### Distribution

Ayeyarwady, Sittaung, Bilin, and Salween basins, Myanmar.

***Hemiclepsis schrencki*** **Bolotov**, **Klass**, **Bespalaya**, **Konopleva**, **Kondakov & Vikhrev sp**. **nov**.

Figures 3K, 4K, 5K, Table 2, Supplementary Table 4, Supplementary Fig. 10H.

Holotype RMBH **Hir_0091_1-H**, RUSSIA: Primorye Region, Partizanskaya River, 43.0585°N, 133.1540°E, 27.v.2017, Bolotov leg.

#### Paratypes

RUSSIA: Primorye Region, Ussuri Basin, Muravievka River, 43.7703°N, 133.2611°E, on a stone, 19.v.2017, one specimen [RMBH **Hir_0088_1**], Bolotov leg.

#### Etymology

This species is named in memory of Academician Leopold von Schrenck (1826–1894), a famous Russian zoologist and explorer of Northern Asia.

#### Morphological diagnosis

Medium-sized leech, which could be distinguished from other congeners by a combination of the following characters: dorsum smooth, orange, with seven rows of ovate yellow spots (spots in the lateral rows are located on the edge of the last annulus of each somite); posterior sucker with large yellow spots (Supplementary Table 4). This novel species resembles *Hemiclepsis marginata* but could be distinguished from it by larger, ovate yellow spots (vs. smaller, rounded spots) and the presence of the central row of spots (vs. the lack of this feature).

#### Molecular diagnosis

The new species differs from other congeners by 15 fixed nucleotide substitutions in the *COI* gene fragment (Supplementary Table 3). Genetically, it is most closely related to *Hemiclepsis khankiana* **sp**. **nov**. (mean *COI* p-distance = 9.1%).

#### Life style

A free-living leech species (Table 1).

#### Distribution

Partizanskaya and Ussuri basins, Primorye Region, Russia.

***Hemiclepsis tumniniana*** **Bolotov**, **Klass**, **Bespalaya**, **Konopleva**, **Kondakov & Vikhrev sp**. **nov**.

Figures 3L, 4L, 5L, Table 2, Supplementary Table 4, Supplementary Figs 8C, 10I.

Holotype RMBH **Hir_0093-H**, RUSSIA: Khabarovsk Region, Tumnin River, 50.0001°N, 139.9175°E, silty-gravel bottom with macrophytes and algae, on a stone, 17.vii.2014, Bolotov and Vikhrev leg.

#### Paratypes

RUSSIA: type locality, same date and collectors, 3 specimens [RMBH **Hir_0014**, **Hir_0235**]; Tumnin River, 49.9451°N, 139.9181°E, 14.vii.2014, 3 specimens [RMBH **Hir_0001**], Bolotov and Vikhrev leg.

#### Etymology

The name of this species is derived from the Tumnin River, from which the type series was collected.

#### Morphological diagnosis

Small leech, which could be distinguished from other congeners by a combination of the following characters: dorsum brown or orange, with seven rows of white or yellow spots (spots of the central row are joined to a broad white or yellow stripe, while spots in other rows may disappear in large specimens); seven rows of very low tubercles; posterior sucker whitish, with dense, diffuse brown dots (Supplementary Table 4).

#### Molecular diagnosis

The new species differs from other congeners by 27 fixed nucleotide substitutions in the *COI* gene fragment (Supplementary Table 3). Genetically, it is most closely related to *Hemiclepsis kasmiana* **comb**. **rev**. (mean *COI* p-distance = 9.5%).

#### Life style

A free-living leech species (Table 1).

#### Distribution

Tumnin River, Khabarovsk Region, Russia.

## Discussion

### Species-rich mussel leech assemblage and cryptic diversity of leeches

Here, we report the discovery of a species-rich assemblage of leeches being associated with freshwater mussels (Unionida). In the Old World, this assemblage is known from East and Southeast Asia, India, Nepal, and Africa (Fig. 1 and Table 1). It includes members of two genera, *Batracobdelloides* (six species) and *Hemiclepsis* (three species) that were largely overlooked by researchers. We describe seven species new to science from East and Southeast Asia as a supplement to three mussel-associated leech taxa already known from the Old World, i.e. *Batracobdelloides tricarinatus* (Africa)^26,27^, *B*. *reticulatus* (India and Nepal)^23–25^, and *Hemiclepsis kasmiana* **comb**. **rev**. (East Asia)^18,20,21^. Although they might have been overlooked, mussel-associated leeches have not been reported from Europe, Middle East, North and Central Asia, Australia, the Indonesian Archipelago, New Guinea, and the Philippines. In the New World, two leech species, i.e. *Placobdella montifera* and *P*. *parasitica*, were repeatedly recorded in freshwater mussels from North America^1,2,10–12,14^, while such findings from South America are still lacking. In total, 12 glossiphoniid leech species are associated with freshwater mussels globally. The species richness of free-living leeches in Asia also seems to be largely underestimated, because an integrative re-analysis of widespread Palearctic species such as *Hemiclepsis marginata* reveals the presence of cryptic taxa with restricted ranges, e.g. *H*. *schrencki* **sp**. **nov**. (a vicariate species replacing *H*. *marginata* in the Amur River and smaller freshwater basins of the Japan Sea drainage) and *H*. *tumniniana* **sp**. **nov**. (a possible endemic lineage to the Tumnin River and, probably, several nearest river systems). Our findings are in agreement with previous researches indicating that nominal species of leeches with broad distribution throughout Eurasia actually represent species complexes containing one or several endemic species-level lineages in various regions of the Asian Subcontinent^31^.

### Origin of the mussel leech assemblage

The leech association with freshwater mussels evolved independently in three genera: *Batracobdelloides*, *Hemiclepsis*, and *Placobdella*. The *Hemiclepsis* mussel-associated leeches seem to be the most ancient clade originated near the Oligocene – Miocene boundary (Fig. 2). Four *Batracobdelloides* species from Myanmar belong to a clade that is fully supported by all the phylogenetic analyses. This clade originated in the Late Miocene. The phylogenetic position of *Batracobdelloides koreanus* **sp**. **nov**. is still uncertain. This species was recovered as sister to the African *B*. *tricarinatus* + *B*. *amnicolus* clade by the maximum likelihood and MrBayes phylogenies (Supplementary Figs 2–3). In contrast, the BEAST phylogeny indicates that it is sister to a clade containing four *Batracobdelloides* mussel leech species from Myanmar (Fig. 2). Here, we chose the latter hypothesis as corresponding to the biogeographic patterns, and in this case, the Asian *Batracobdelloides* mussel leeches form a monophyletic clade of the mid-Miocene origin. As for the *Placobdella* taxa being associated with freshwater mussels, they represent two independent lineages within the genus (Supplementary Figs 1 and 4). In general, two monophyletic clades of leeches evolved in close association with freshwater mussels as early as the Miocene, and several additional lineages have originated independently as facultative inhabitants (parasites or commensals) of the mussel mantle cavity.

### Slow evolutionary rates and historical biogeography of the mussel leeches

Our fossil-calibrated phylogenetic model suggests that leeches are characterized by slow rates of molecular evolution, with the mean *COI* substitution rate of 0.63%/site/Myr (95% HPD 0.51–0.75%/site/Myr). Slow substitution rates are a common feature for several “living fossil” taxa, e.g. freshwater mussels^32,33^, coelacanths^34^, anthozoans^35,36^, sturgeons^37^, and puddle fishes^37^. To the best of our knowledge, we report the first reliable substitution rates for the Hirudinea based on a fossil-calibrated phylogeny, and these values can be applied as external rates to calculate time-calibrated phylogenetic models in the future. Our biogeographic reconstructions strongly support the New World origin for the Haementeriinae and the East Asian origin for the Glossiphoniinae. Two mussel-associated leech clades belonging to the genera *Batracobdelloides* and *Hemiclepsis* appear to have originated in East and Southeast Asia with subsequent vicariance and intra-area radiation events. These regions are among the most species-rich hotspots of freshwater bivalve diversity at the global scale^38–42^, and the ancient and diverse freshwater mussel faunas most likely supported the origin and radiation of mussel-associated leeches in Asia. The BEAST phylogeny reveals that the African *Batracobdelloides* taxa were separated from Asian members of this genus in the Early Miocene that corresponds to similar Miocene vicariance events in other freshwater animals such as the radicine pond snails^43^. These events coincide with the period of direct connection between African and Eurasian plates via Middle East since the closure of the Tethys in the Early Miocene (approximately 20 Myr)^44^.

### Association of leeches with freshwater mussels

Previous researchers chiefly advocated in favor of the “clandestine shelter” hypothesis that assumes occasional commensal relationship of leeches with freshwater mussels^1,2,10–12,14^. This assumption was based on field observations in North America, revealing a low infestation rate of freshwater mussels by leeches, i.e. *Placobdella montifera* and *P*. *parasitica*^1,10–12^. In southwestern Louisiana, 21 freshwater mussels were infested with *Placobdella montifera* among 2,300 mussel specimens examined (*LIP* = 0.91%), and only 28 adult leeches were found in this sample (*ILI* = 0.01 l.p.m.)^10^. Our novel data from Asia and Africa reveals that at least two clades of mussel-associated leech species could be considered obligate inhabitants of the mantle cavity of freshwater mussels serving as shelter. Furthermore, we propose that larvae and juvenile mussel-associated leeches could feed on mucus and body fluids of freshwater mussels representing secondary hosts for these leech species^18,20,21^. Molecular sequences of the digestive system content of the adult mussel-associated leeches indicate that they leave their mussel hosts periodically to obtain blood of freshwater fishes serving as the primary hosts. Probably, adult leeches need to use one or several higher-calorie fish blood meals instead of nutritionally sparse mussel haemolymph to complete the life cycle, i.e. to ensure the successful development of eggs. This hypothesis agrees with the idea that evolution of blood-sucking leeches has been moving toward parasitism of animals with a nutrient-rich blood^45^. Such a two-host feeding behavior (both obligate and facultative), when fish blood meals are needed at the final stage of the life cycle just before leech reproduction, appears to be a successful adaptation to freshwater environment, in which availability of vertebrate blood is limited, and many leech species are forced to use nutrient-poor body fluids of invertebrates as the primary feeding source^45^.

It has been suggested that the primary selective pressure driving the evolution of parental care in leeches may have been predation on leech eggs and juvenile stages^46^. From this point of view, a hidden life style of mussel-associated leeches inside the mantle cavity of freshwater mussels could be considered a progressive evolutionary trait in brooding behavior helping to protect juvenile stages from predators. There are a few records of other leech species inside the mantle cavity of other freshwater bivalve groups, e.g. Sphaeriidae^14,47^ and Dreissenidae^2,48^. However, none of these bivalve inhabitants were reported in association with the Unionida. Although a snail-associated leech assemblage is also poorly known, it seems to be a species-rich entity, with at least eleven leech taxa using freshwater gastropods as hosts only in North America^14^. *Stibarobdella moorei*, a unique example of a cephalopod-associated marine leech, uses *Octopus bimaculatus* (Octopodidae) as the primary host^49^. Association of *Alboglossiphonia* leeches with freshwater bryozoans recorded in Siberia is another unusual example, illustrating a rather “clandestine shelter” commensalism than a host-parasite relationship^50^. In soft-bottom environments, the marine leech *Notostomum cyclostomum* (Piscicolidae) uses crab exoskeletons as the hard substrate for its cocoon, but this leech species does not feed on crustaceans but on fish blood^51^. These examples highlight that leeches use various invertebrate taxa as hosts, shelters or brooding substrates and that such hidden associations of Hirudinea with other animal groups may be much more common than it was assumed previously.

## Methods

### Data sampling

Mussel-associated leeches (*N* = 1,334 specimens) were collected from the mantle cavity of 3,045 freshwater mussels (Unionida: Unionidae, Iridinidae, and Margaritiferidae) using forceps during our broad-scale survey of freshwater mussels in East Asia (Russian Far East, South Korea, and Japan), Southeast Asia (Myanmar) and East Africa (Uganda). For most samples, the number of adult and juvenile leeches (without larvae) in every mussel specimen was recorded (Supplementary Tables 6–7 and 9 and Supplementary Dataset 1) to estimate the prevalence and intensity of leech infestation in each freshwater mussel sample^21,49^. In order to reveal the life cycle of mussel-associated leeches, from every examined mussel specimen we recorded: (1) the presence and position of leeches brooding on the host shell; (2) the presence of mature leeches carrying eggs (*Batracobdelloides*) and those with larvae attached to their abdomen; (3) the presence of leech larvae attached to the host mussel soft body; (4) the presence of juvenile leeches; and (5) the presence of adult leeches (Supplementary Tables 6 and 7). In the same sampling sites, free-living leeches were collected from stones, wood fragments, tree branches, and macrophytes using forceps. The leeches were fixed in 96% ethanol immediately after collecting. The fixation of leeches was not preceded by anesthesia in 10% ethanol solution to avoid DNA degradation in the hot tropical and subtropical environments where these samples were primarily collected. A few additional mussel leech samples (*N* = 26 specimens) were obtained from the mantle cavity of freshwater mussel specimens in the collection of the Federal Scientific Center of the East Asia Terrestrial Biodiversity, Far Eastern Branch of the Russian Academy of Sciences, Vladivostok, Russia. Images of live leech specimens and their broods were recorded with a digital camera (Canon EOS 7D, Canon Inc., Japan).

### Molecular analyses

New molecular sequences (*COI* and partly *18S rRNA*) were generated from tissue samples of 118 leech specimens. Additionally, we obtained *COI* sequences of the crop content that was extracted from 14 mature leech specimens belonging to 9 mussel-associated leech species and 2 free-living taxa (Supplementary Table 8) to estimate taxonomic affinities of the primary host species (freshwater fishes). Total genomic DNA was extracted from 96% ethanol-preserved samples using the NucleoSpin^®^ Tissue Kit (Macherey-Nagel GmbH & Co. KG, Germany), following the manufacturer protocol. Primer sequences and PCR conditions are shown in Supplementary Table 10. Forward and reverse sequence reactions were performed directly on purified PCR products using the ABI PRISM^®^ BigDye™ Terminator v3.1 reagents kit and run on an ABI PRISM^®^ 3730 DNA analyzer (Thermo Fisher Scientific Inc., Waltham, MA, USA). Resulting sequences were checked by eye using BioEdit v7.2.5^52^. Phylogenetic relations of the haplotypes were retrieved with the BOLD *COI* Full Database (BOLD)^53^ and with GenBank using a Basic Local Alignment Search Tool, BLAST^54^.

### Phylogenetic analyses

To reconstruct the phylogeny of the Hirudinea, we sampled a *COI*+ *18S rRNA* sequence dataset containing 109 unique haplotypes of members of the Glossiphoniiformes, Erpobdelliformes, Hirudiniformes, and Oceanobdelliformes (Supplementary Table 2). Additional sequences were obtained from GenBank. Five haplotypes of the Oligochaeta taxa were used as outgroup. The sequence alignment was performed for each gene separately using the MUSCLE algorithm implemented in MEGA7^55^. The *18S rRNA* alignment was checked through the Gblocks 0.91b online server^56^ to exclude hypervariable sites (14% of the original 2,017 bp). To estimate each partition (*COI* and *18S rRNA*) for evidence of substitution saturation, we computed the Xia *et al*.’s test^57^ with DAMBE v5.3.108^58^. This test revealed a little saturation effect in the two partitions even under the assumption of an asymmetrical tree. A partition-homogeneity test with heuristic search through PAUP* v4.0a165^59^ shared the significant conflict of phylogenetic signals among the partitions in the dataset (*P* = 0.01). However, we considered that this conflict does not affect the phylogeny because it seems to reflect a copious homoplasy rather than independent histories of the genes^38,60^. The single gene alignments were joined to a two-locus alignment using FaBox v1.5 (http://users-birc.au.dk/palle/php/fabox)^61^. Maximum likelihood phylogenetic analyses were carried out with an online version of IQ-TREE v1.6.11^62^ using an ultrafast bootstrap algorithm^63^ and an automatic identification of the most appropriate substitution models^64^. We used IQ-TREE because this software package was found to return phylogenetic reconstructions with best-observed likelihoods compared with other available likelihood-based algorithms^65^. Bayesian phylogenetic reconstructions were performed using MrBayes v3.2.6^66^. Two independent runs, each with one cold and three heated (temperature = 0.1) MCMC chains, were conducted for 25 million generations (sampling every 1,000th generation). Convergence of the MCMC chains to the stationary distribution was assessed visually based on the plotted posterior estimates with Tracer v1.7^67^, and 15% of the sampled trees were discarded as an appropriate burn-in. Bayesian calculations were performed at the San Diego Supercomputer Center through the CIPRES Science Gateway^68^. The best-fit evolutionary models applied to each partition (3 codons of *COI*+ *18S rRNA*) in the MrBayes and IQ-TREE runs are presented in Supplementary Table 11.

### Species delimitation and diagnostics of the new taxa

To diagnose the new species, we used two-step procedure based on the phylogenetic and morphological analyses^39^. First, we applied an automatic species delimitation approach to delimit the Molecular Operational Taxonomic Units (MOTUs) that may correspond to biological species. The Glossiphoniidae *COI* sequence dataset was compiled using our own data (111 sequences) and available materials obtained from GenBank (421 sequences). These sequences were collapsed to 316 unique haplotypes. The *COI* haplotype of an unidentified Piscicolidae taxon was used as outgroup. The species delimitation was performed using the Poisson Tree Process (PTP) modeling through the PTP web-service (http://mptp.h-its.org)^69^. This approach seems to be more appropriate for slowly evolving animal groups such as freshwater mussels^39^ and leeches. As an input tree, we used a maximum likelihood consensus phylogeny inferred from an online version of IQ-TREE v1.6.11^62^ with an ultrafast bootstrap algorithm^63^. Substitution models applied to each codon position are listed in Supplementary Table 11. The resulting PTP species delimitation model was largely congruent with the modern taxonomy of the Glossiphoniidae, supporting the majority of currently accepted species (Supplementary Fig. 4). Based on this evidence, we concluded that it is an appropriate model to delimit the species-level units in our dataset. An uncorrected *COI* mean p-distance to the nearest neighbor of each lineage was calculated in MEGA7^55^. Second, each MOTU within the clades of interest was studied using morphological and biogeographic criteria and was compared with the original descriptions of nominal taxa to link each clade to a biological species. Nine species lacking available names are described under this study as new to science. The molecular diagnosis of every new taxon was designed using fixed nucleotide substitutions, which were estimated for each gene separately using a Toggle Conserved Sites tool of MEGA7^55^ at 50% level. For the diagnoses, an alignment of congeneric haplotype sequences was performed using the Muscle algorithm implemented in MEGA7^55^. All deleterious mutations were retained for the analyses.

### Morphological study

The external morphological characters (number and position of eyes, annulation, color, papillation, position of genital pores, and body size)^70^ were examined on specimens of the new species and related taxa. Body measurements for the new leech species were performed using a stereomicroscope Leica M165C (Leica Microsystems GmbH, Germany) equipped with an ocular-micrometer as follows: body length (BL), body width (BW), width of anterior sucker (AW), and width of posterior sucker (PW). To study the reproductive and digestive systems, leeches were dissected using a standard approach^70^. The images of specimens and their morphological and anatomical details were taken with stereomicroscopes Leica M165C (Leica Microsystems GmbH, Germany) and Zeiss Axio Zoom.V16 (Carl Zeiss AG, Germany).

### Divergence dating and substitution rate estimation

Node ages were estimated with BEAST v1.10.4^71^ using the same two-locus dataset as for the IQ-TREE and MrBayes phylogenetic analyses (see above). Substitution models assigned to each partition are listed in Supplementary Table 11. A lognormal relaxed clock and Yule speciation process with continuous quantile parametrization were applied as the priors of the fossil-calibrated Bayesian model. To dating the phylogeny, we used one new crown fossil calibration as follows: †Hirudinea indet. Hard minimum age: 210 Ma (Late Triassic). Diagnosis and phylogenetic placement: This fossil from a fluvio-lacustrine deposit was identified as a leech cocoon^72^. We assume that this freshwater fossil could represent a crown lineage of the clade Glossiphoniiformes + (Hirudiniformes + Erpobdelliformes), because the suborder Oceanobdelliformes contains the primarily marine and brackish water families^73^, earlier members of which were rather marine worms (our unpublished data based on an ancestral area reconstruction analysis). Absolute age estimate: Late Triassic, a ∼80-m-thick succession of coal-bearing fluvio-lacustrine deposits, Section Peak Formation (Victoria Group, Beacon Supergroup), Timber Peak in the Eisenhower Range, north Victoria Land, East Antarctica, ~210 Ma, based on stratigraphy and palynological analyses^72^; 95% soft upper bound 237 Ma (mid-Triassic) based on the comprehensive fossil-calibrated phylogeny of extant annelids^74^. Prior settings: exponential distribution, mean (lambda) = 7.3, MRCA: *Batracobdelloides conchophylus* **sp**. **nov**. **-** *Hirudo orientalis* Utevsky & Trontelj, 2005. Three runs, each with 100,000,000 generations, were performed at the San Diego Supercomputer Center (SDSC, University of California, San Diego, USA) through the CIPRES Science Gateway^68^. The resulting log files were checked for convergence of the MCMC chains with Tracer v1.7^67^. All the ESS values were recorded >300. The sets of fossil-calibrated trees inferred from the three runs were joined with LogCombiner v1.10.4^71^ applying a 10% burn-in and an additional re-sampling at every 10,000 generation. The resulting set included 27,000 binary fossil-calibrated trees, based on which a consensus fossil-calibrated phylogenetic tree was obtained with TreeAnnotator v1.10.4^71^. The *COI* and *18S rRNA* substitution rates (mean values and 95% HPD) were obtained from the combined log file using Tracer v1.7^67^.

### Ancestral area and life style reconstructions

For the ancestral trait and area reconstructions with RASP v3.2^75^, we used the set of 27,000 fossil-calibrated binary trees that were combined from three runs of BEAST v1.10.4 (see above). As a condensed tree, we used the user-specified, fossil-calibrated consensus tree, which was calculated based on this set of trees using TreeAnnotator v1.10.4 (see above). Non-Glossiphoniidae sequences were removed from the tree set with the appropriate option of the software. Ancestral area patterns were reconstructed using three probabilistic algorithms: Statistical Dispersal-Vicariance Analysis (S-DIVA), Dispersal-Extinction Cladogenesis (Lagrange configurator, DEC), and Statistical Dispersal-Extinction Cladogenesis (S-DEC)^75^. Seven distribution areas of the in-group taxa were assigned as follows: (A) Africa; (B) East Asia (in a broad sense with Eastern Siberia); (C) Southeast Asia; (D) North America; (E) Europe; and (F) South America (Supplementary Table 2). Several unlikely range constraints (i.e. AD, AF, BF, CD, CE, CF, and EF) were removed from the prior settings of the analyses. The S-DIVA analyses were calculated with the following parameters: max areas = 2; allow reconstruction with max reconstructions = 100; max reconstructions for final tree = 1,000; and allowing extinctions. The DEC and S-DEC analyses were performed with default settings and max areas = 2. In addition to the reconstructions obtained from each analysis separately, we used summary results of all three kinds of analyses, which were combined with RASP v3.2^75^. To reconstruct ancestral life style patterns, we used a Bayesian MCMC analysis^75^. Three life style types were coded as follows: (A) leeches with a hidden life style within the mantle cavity of freshwater mussels; (B) free-living leeches; and (AB) free-living leeches with a hidden stage inside a mussel. The analysis was computed with 500,000 generations (sampling every 100th generation) and 10 MCMC chains (temp = 0.1). Null distribution was not allowed. To exclude the pre-convergence part of the simulation, a 10% burn-in was applied.

### Assessment of leech infestation prevalence and intensity in freshwater mussels

Based on our field data (Supplementary Table 9 and Supplementary Dataset 1), the Leech Infestation Prevalence (*LIP*, %) index was computed using the following equation:

1 $$LIP={N}_{{\rm{IM}}}/{N}_{{\rm{M}}}\times 100;$$

where *N*_IM_ represents a total number of freshwater mussels infested by at least one leech in a given sample, and *N*_M_ represents a total number of freshwater mussels in this sample^21,49^.

The intensity of leech infestation (*ILI*, leeches per mussel [l.p.m.]) was calculated using available field data (Supplementary Table 9 and Supplementary Dataset 1) as follows:

2 $$ILI=\sum {N}_{{\rm{L}}}/{N}_{{\rm{M}}};$$

where ∑*N*_L_ represents a total number of leeches collected from the mantle cavity of all freshwater mussels in a given sample, and *N*_M_ represents a total number of freshwater mussels in this sample^21,49^.

To estimate possible differences in the host preference of mussel-associated leeches, we applied the non-parametric Kruskal-Wallis test implemented in Statistica v13.3 (Stat Soft Inc., USA). Calculations were carried out using available data on leech infestation prevalence and intensity in freshwater mussel samples from Southeast Asia and East Asia (Supplementary Dataset 1). The tribe- and subfamily-level affinities of the host mussels were used as a factor with seven (Southeast Asia) and six (East Asia) levels.

### Nomenclatural acts

The electronic edition of this article conforms to the requirements of the amended International Code of Zoological Nomenclature (ICZN), and hence the new names contained herein are available under that Code from the electronic edition of this article. This published work and the nomenclatural acts it contains have been registered in ZooBank (http://zoobank.org), the online registration system for the ICZN. The LSID for this publication is: urn:lsid:zoobank.org:pub:FA8EB5C7-305A-4140-B3AE-CF5ADAD1C22A. The electronic edition of this paper was published in a journal with an ISSN, and has been archived and is available from PubMed Central.

## Supplementary information

Supplementary Info

Dataset 1

Dataset 2

## Data Availability

The type series of the new species are available in the Russian Museum of Biodiversity Hotspots [RMBH], Federal Center for Integrated Arctic Research, Russian Academy of Sciences, Arkhangelsk, Russia. Other leech samples are available in the RMBH and the Non-Molluscan Invertebrate Collection [NCSM-NMI], North Carolina Museum of Natural Sciences, Raleigh, North Carolina, United States of America. The mussel leech specimens collected from freshwater mussels deposited in the Federal Scientific Center of the East Asia Terrestrial Biodiversity, Far Eastern Branch of the Russian Academy of Sciences, Vladivostok, Russia were transferred to the RMBH. The sequences generated in this study are deposited in GenBank. GenBank accession number and collecting locality for each specimen are presented in Supplementary Tables 1 and 2 and Supplementary Dataset 2.
