# Supplementary material for: Freshwater mussels house a diverse mussel-associated leech assemblage
Source: Sci Rep. 2019 Nov 11;9:16449. doi: 10.1038/s41598-019-52688-3 (PMC6848535; doi:10.1038/s41598-019-52688-3)
Supplement: Supplementary file 1 — Supplementary Info [file 41598_2019_52688_MOESM1_ESM.pdf]

SUPPLEMENTARY INFORMATION

Freshwater mussels house a diverse mussel-associated leech assemblage

Ivan N. Bolotov*, Anna L. Klass, Alexander V. Kondakov, Ilya V. Vikhrev, Yulia V. Bespalaya, Mikhail Yu. Gofarov, Boris Yu. Filippov, Arthur E. Bogan, Manuel Lopes-Lima, Zau Lunn, Nyein Chan, Olga V. Aksenova, Gennady A. Dvoryankin, Yulia E. Chapurina, Sang Ki Kim, Yulia S. Kolosova, Ekaterina S. Konopleva, Jin Hee Lee, Alexander A. Makhrov, Dmitry M. Palatov, Elena M. Sayenko, Vitaly M. Spitsyn, Svetlana E. Sokolova, Alena A. Tomilova, Than Win, Natalia A. Zubrii & Maxim V. Vinarski

*Corresponding author: inepras@yandex.ru

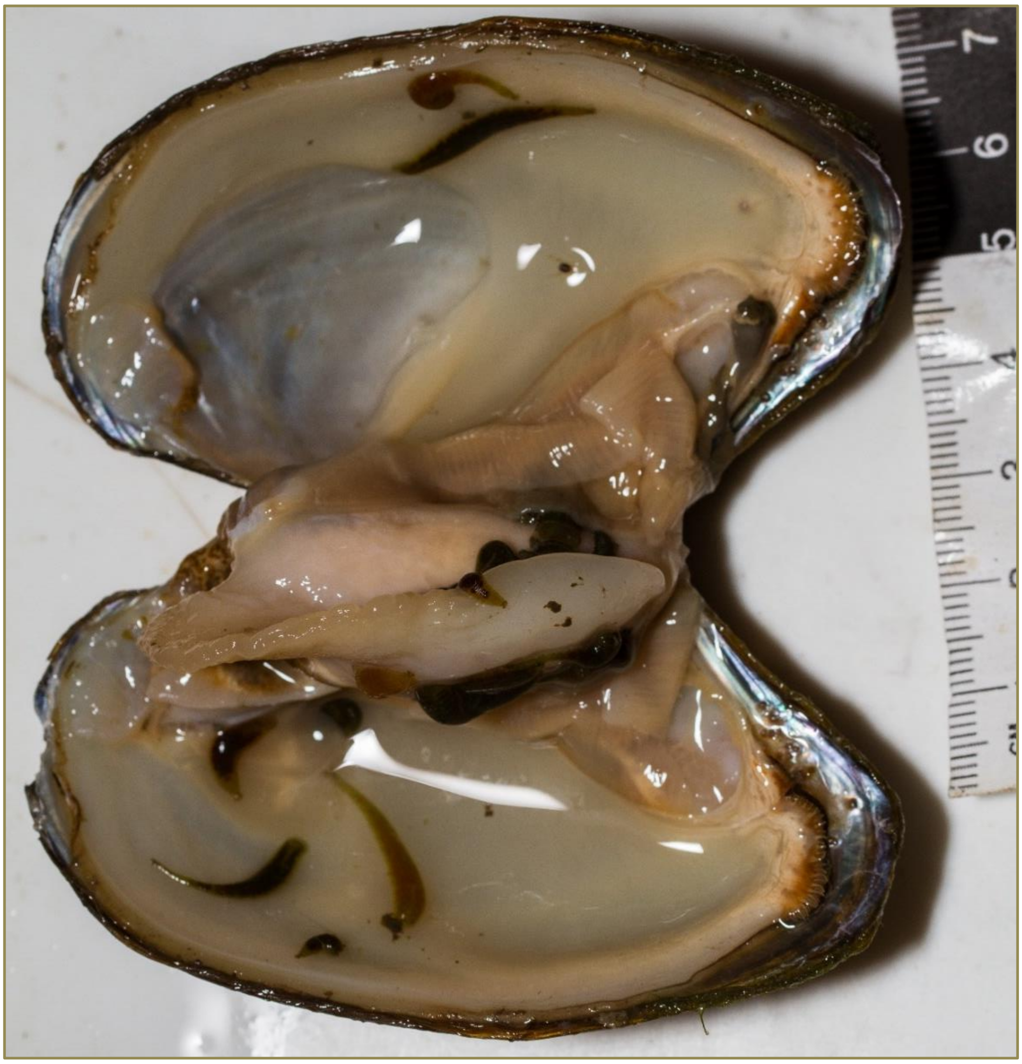

Mature mussel leeches *Hemiclepsis myanmariana* **sp. nov.** [sample RMBH Hir_0048_1] in the mantle cavity of *Lamellidens savadiensis* (Unionidae), Nadi Lake, Salween Basin, Myanmar, 23.ii.2018 (Photo: Ilya V. Vikhrev).

Contents

Supplementary Figures

Supplementary Figure 1. IQ-TREE maximum likelihood phylogeny of the Hirudinea (four partitions: three codons of *COI* + *18S rRNA*).

Supplementary Figure 2. Bayesian phylogeny of the Hirudinea (four partitions: three codons of *COI* + *18S rRNA*).

Supplementary Figure 3. Two-locus fossil-calibrated phylogeny of the Hirudinea calculated under a lognormal relaxed clock model and a Yule process speciation implemented in BEAST 1.10.4 (four partitions: three codons of *COI* + *18S rRNA*).

Supplementary Figure 4. Results of the Poisson Tree Process (PTP) species delimitation analysis based on the *COI* haplotype phylogeny of the Glossiphoniidae (*N* = 316 in-group haplotypes) inferred from IQ-TREE.

Supplementary Figure 5. Historical biogeography of the Glossiphoniidae inferred from three different statistical modeling approaches, including (A) the combined results of SDIVA, DEC and S-DEC; (B) S-DIVA; (C) DEC; and (D) S-DEC based on the fossil-calibrated phylogeny.

Supplementary Figure 6. Ancestral life style reconstruction of the Glossiphoniidae inferred from Bayesian MCMC analysis based on the fossil-calibrated phylogeny.

Supplementary Figure 7. Morphological variability of new *Batracobdelloides* species (dorsal view).

Supplementary Figure 8. Morphological variability of new *Hemiclepsis* species (dorsal view).

Supplementary Figure 9. Morphological variability of *Hemiclepsis kasmiana* **comb. rev.** (dorsal view).

Supplementary Figure 10. Digestive system (dorsal view) of the new species, *Batracobdelloides tricarinatus*, and *Hemiclepsis kasmiana* **comb. rev.**

Supplementary Figure 11. Samples of freshwater mussels (Unionida: Unionidae, Margaritiferidae, and Iridinidae) collected from various freshwater basins in East Asia, Southeast Asia, and East Africa and their infestation by mussel-associated leeches (primary data are given in Supplementary Dataset 1).

Supplementary Tables

Supplementary Table 1. List of *COI* and *18S rRNA* gene sequences of the leech genera *Batracobdelloides* and *Hemiclepsis* (Glossiphoniidae) used in this study.

Supplementary Table 2. List of *COI* and *18S rRNA* gene sequences used in phylogenetic reconstruction of the Glossiphoniidae.

Supplementary Table 3. Summary of molecular diagnoses of the new leech species in the genera *Batracobdelloides* and *Hemiclepsis* (Glossiphoniidae).

Supplementary Table 4. Morphological description of the new leech species in the genera *Batracobdelloides* and *Hemiclepsis* (Glossiphoniidae) based on ethanol-preserved specimens.

Supplementary Table 5. The most probable ancestral areas of the primary clades within the Glossiphoniidae inferred from three different statistical modeling approaches.

Supplementary Table 6. Field observations supporting the general scheme of the life cycle of *Hemiclepsis* mussel-associated leech taxa (Fig. 6).

Supplementary Table 7. Field observations supporting the general scheme of the life cycle of *Batracobdelloides* mussel-associated leech taxa (Fig. 6).

Supplementary Table 8. Molecular identification of the primary hosts based on molecular analyses of the crop content of mature mussel-associated leech species and two free-living leech taxa new to science

Supplementary Table 9. Infestation of freshwater mussels by leeches in East Asia, Southeast Asia, and East Africa.

Supplementary Table 10. Primer sequences and PCR conditions.

Supplementary Table 11. Models of sequence evolution for each partition used in phylogenetic reconstructions.

Supplementary Notes

Supplementary Note 1. Checklist of the genera *Batracobdelloides* Oosthuizen, 1986 and *Hemiclepsis* Vejdovsky, 1884.

Supplementary Note 2. Key to mussel-associated leech species (Glossiphoniidae: *Batracobdelloides* and *Hemiclepsis*) of the Old World.

Supplementary References

Supplementary Datasets

Supplementary Dataset 1. Samples of freshwater mussels (Unionidae) collected in East Asia, Southeast Asia, and East Africa and their infestation by mussel-associated leeches [separate MS Excel file].

Supplementary Dataset 2. List of *COI* sequences of the Glossiphoniidae used in the Poisson Tree Process (PTP) species delimitation modeling (Supplementary Fig. 4) [separate MS Excel file].

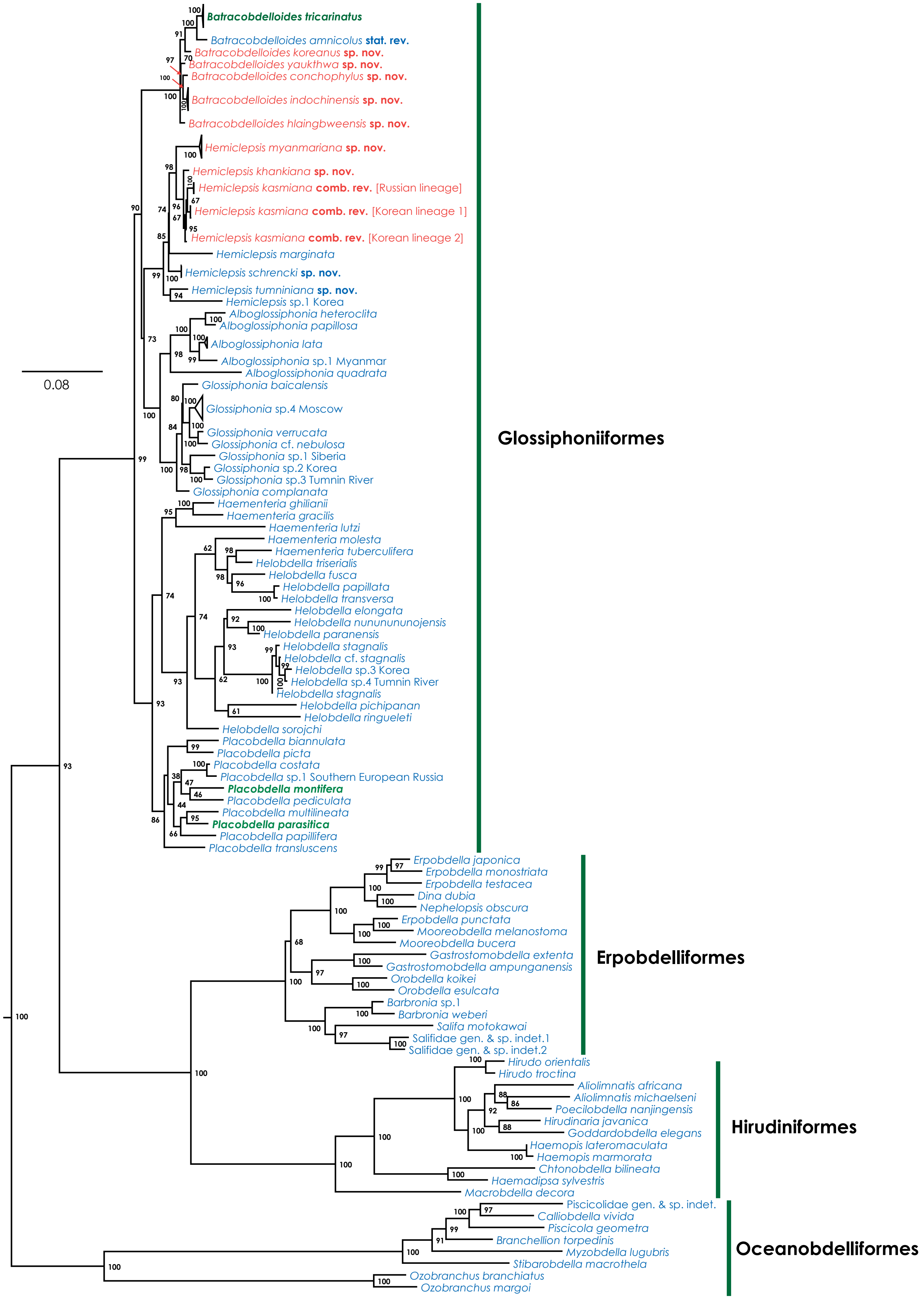

Supplementary Figure 1. IQ-TREE maximum likelihood phylogeny of the Hirudinea (four partitions: three codons of COI + 18S rRNA). Black numbers near nodes are bootstrap support values of IQ-TREE. Scale bar indicates the branch lengths. The species names of proposed obligate inhabitants of the mantle cavity of freshwater mussels are colored red. The names of free-living leech taxa with a facultative hidden stage inside the mantle cavity of freshwater mussels are colored green. The names of free-living leech species are colored blue. Outgroup taxa are not shown.

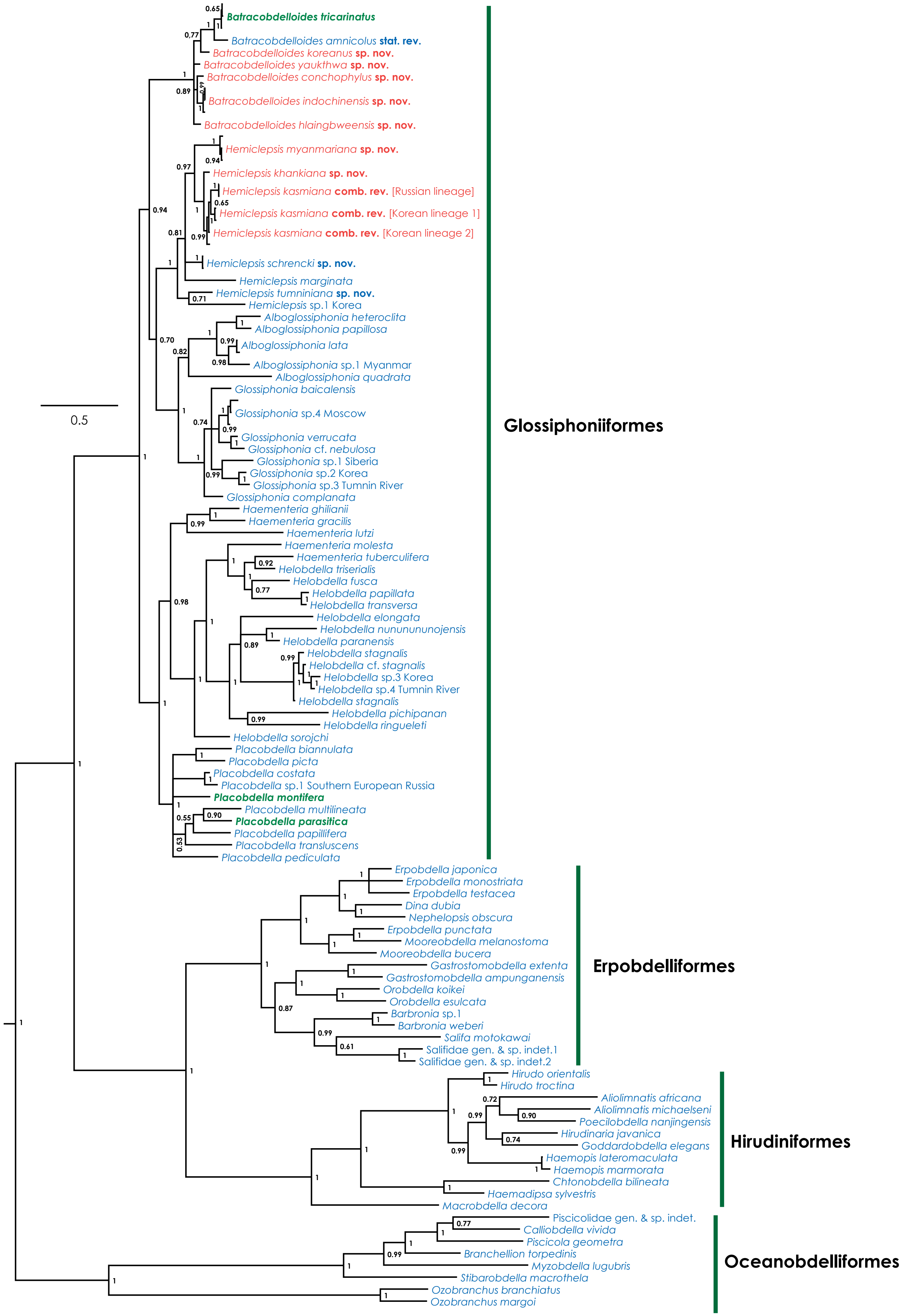

Supplementary Figure 2. Bayesian phylogeny of the Hirudinea (four partitions: three codons of COI + 18S rRNA). Black numbers near nodes are BPP values of MrBayes. Scale bar indicates the branch lengths. The species names of proposed obligate inhabitants of the mantle cavity of freshwater mussels are colored red. The names of free-living leech taxa with a facultative hidden stage inside the mantle cavity of freshwater mussels are colored green. The names of free-living leech species are colored blue. Outgroup taxa are not shown.

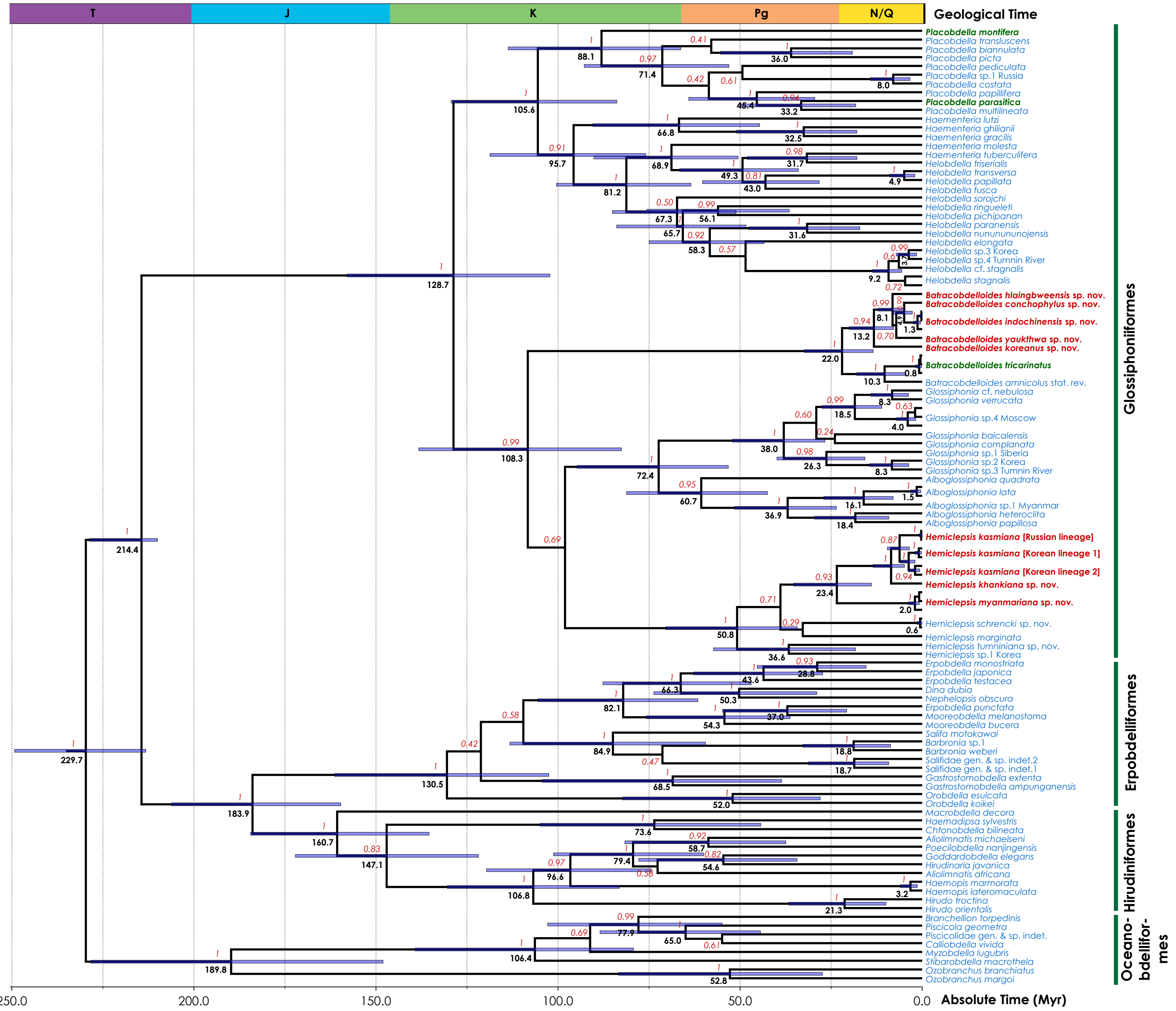

Supplementary Figure 3. Two-locus fossil-calibrated phylogeny of the Hirudinea calculated under a lognormal relaxed clock model and a Yule process speciation implemented in BEAST 1.10.4 (four partitions: three codons of COI + 18S rRNA). Node bars are 95% HPD of the divergence time. Red numbers near nodes are BPP values inferred from BEAST. Black numbers near nodes are node ages (Myr). Divergence times for weakly supported nodes (BEAST BPP < 0.75) are omitted. The species names of proposed obligate inhabitants of the mantle cavity of freshwater mussels are colored red. The names of free-living leech taxa with a facultative hidden stage inside the mantle cavity of freshwater mussels are colored green. The names of free-living leech species are colored blue. Outgroup taxa are not shown.

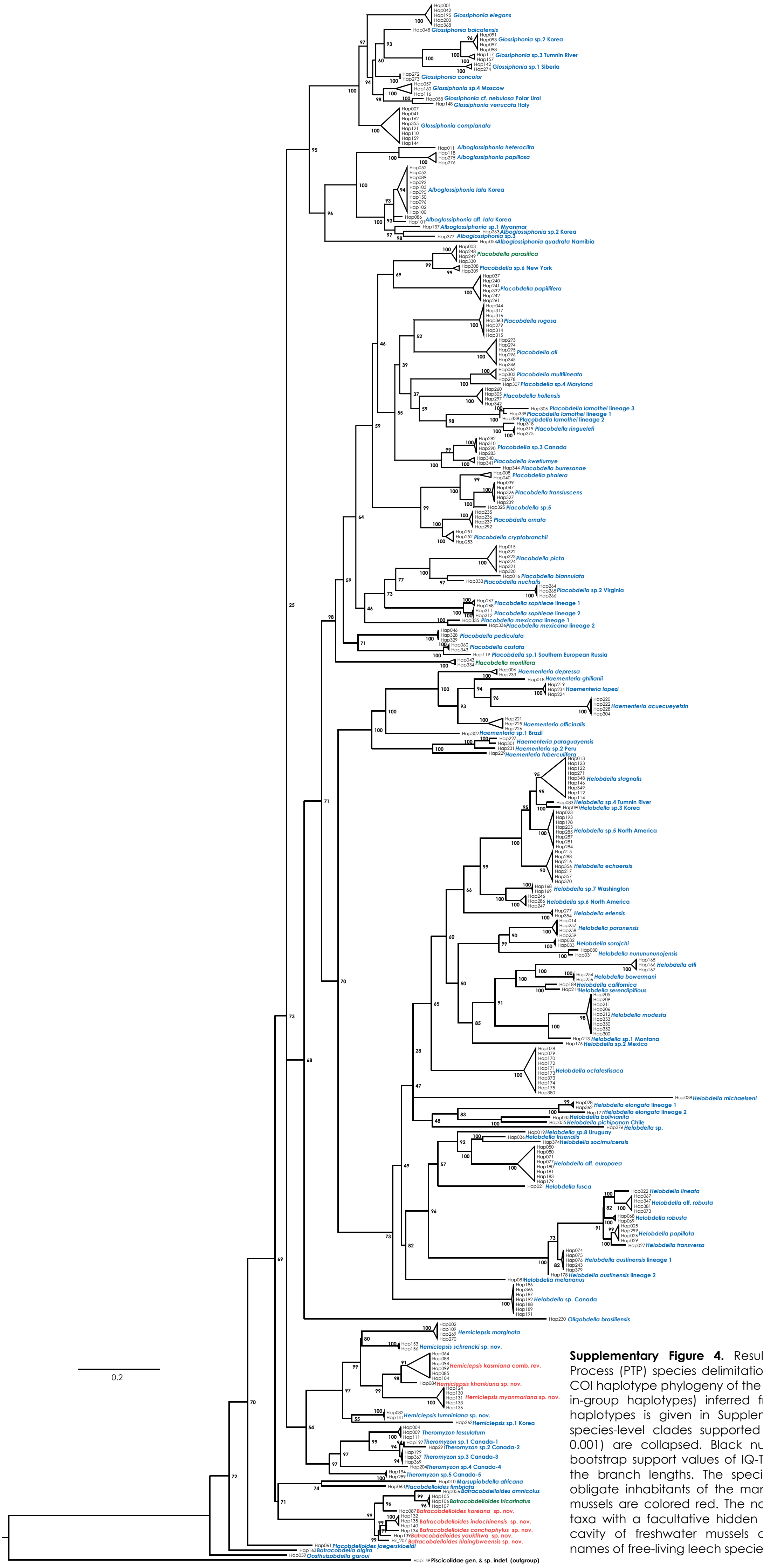

Supplementary Figure 4. Results of the Poisson Tree Process (PTP) species delimitation analysis based on the COI haplotype phylogeny of the Glossiphoniidae (N = 316 in-group haplotypes) inferred from IQ-TREE. The list of haplotypes is given in Supplementary Dataset 2. The species-level clades supported by the modeling ($P < 0.001$) are collapsed. Black numbers near nodes are bootstrap support values of IQ-TREE. Scale bar indicates the branch lengths. The species names of proposed obligate inhabitants of the mantle cavity of freshwater mussels are colored red. The names of free-living leech taxa with a facultative hidden stage inside the mantle cavity of freshwater mussels are colored green. The names of free-living leech species are colored blue.

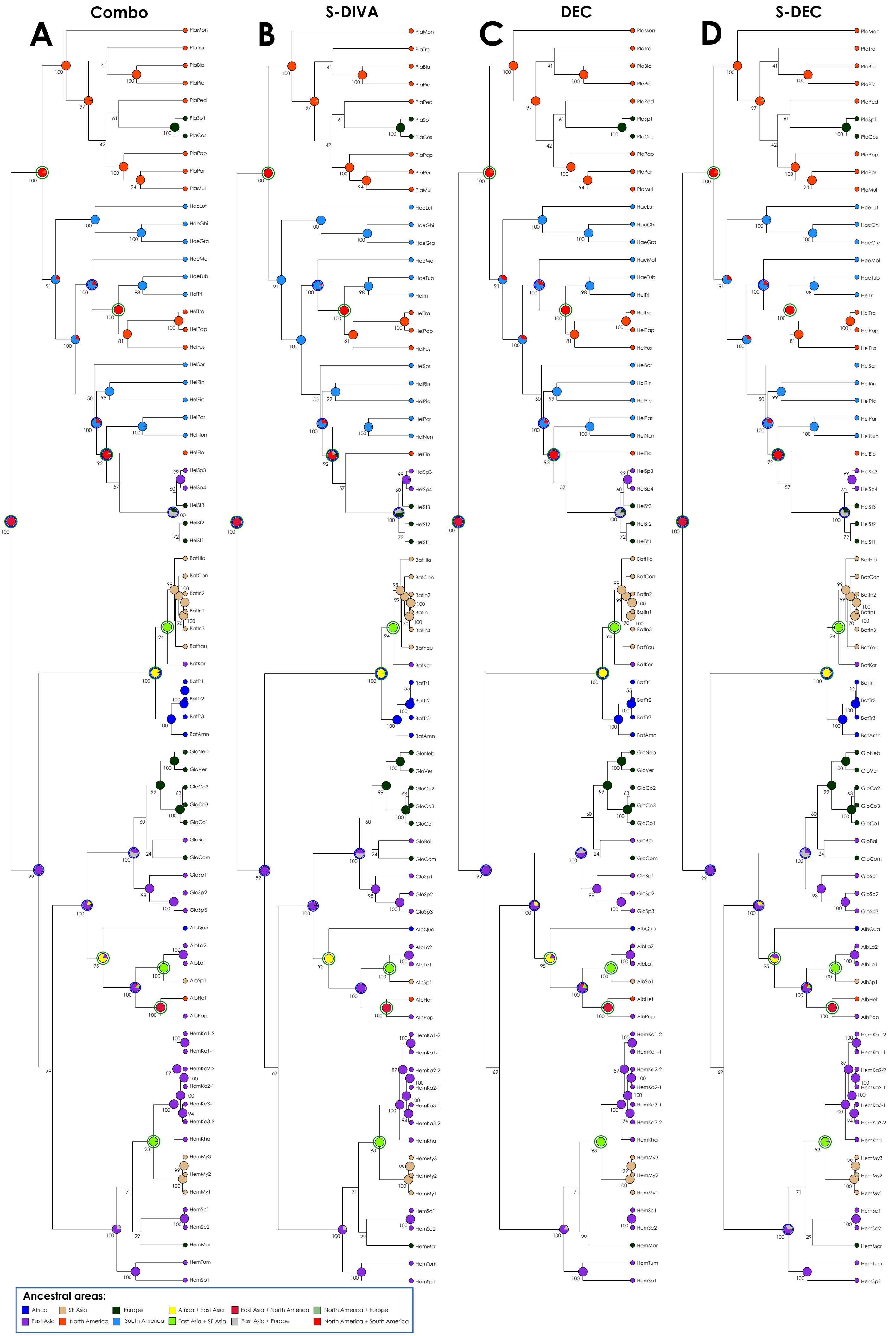

Supplementary Figure 5. Historical biogeography of the Glossiphoniidae inferred from three different statistical modeling approaches, including (**A**) the combined results of SDIVA, DEC and S-DEC; (**B**) S-DIVA; (**C**) DEC; and (**D**) S-DEC based on the fossil-calibrated phylogeny. Pie chaps near nodes indicate probabilities of certain ancestral areas. Color circles on the tip nodes indicate the range of each species. Black numbers near nodes are BPP values inferred from BEAST. Ancestral area reconstructions for weakly supported nodes (BEAST BPP < 0.75) are omitted.

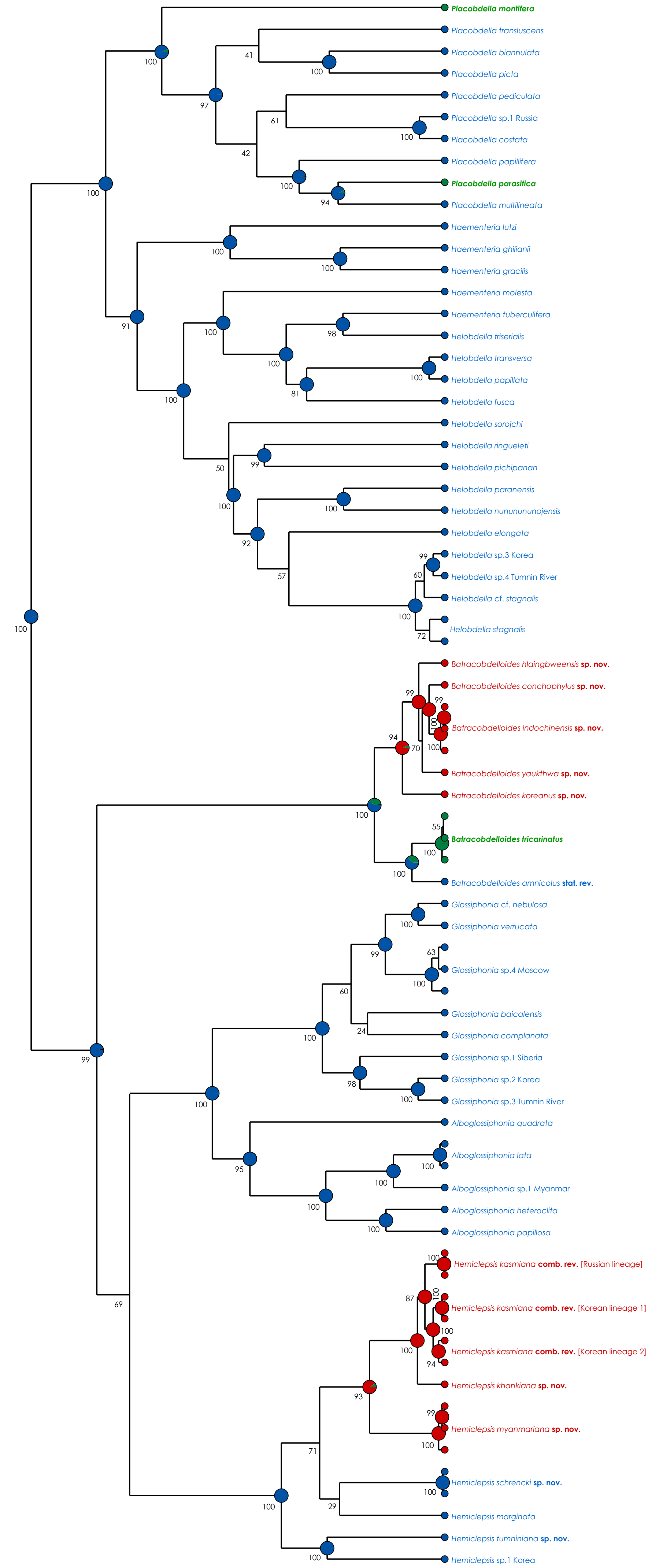

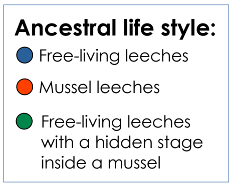

Supplementary Figure 6. Ancestral life style reconstruction of the Glossiphoniidae inferred from Bayesian MCMC analysis based on the fossil-calibrated phylogeny. Pie chaps near nodes indicate probability of a certain ancestral life style. Color circles on the tip nodes indicate the life style of each terminal species. Black numbers near nodes are BPP values inferred from BEAST. Host reconstructions for weakly supported nodes (BEAST BPP < 0.75) are omitted. The species names of proposed obligate inhabitants of the mantle cavity of freshwater mussels are colored red. The names of free-living leech taxa with a facultative hidden stage inside the mantle cavity of freshwater mussels are colored green. The names of free-living leech species are colored blue.

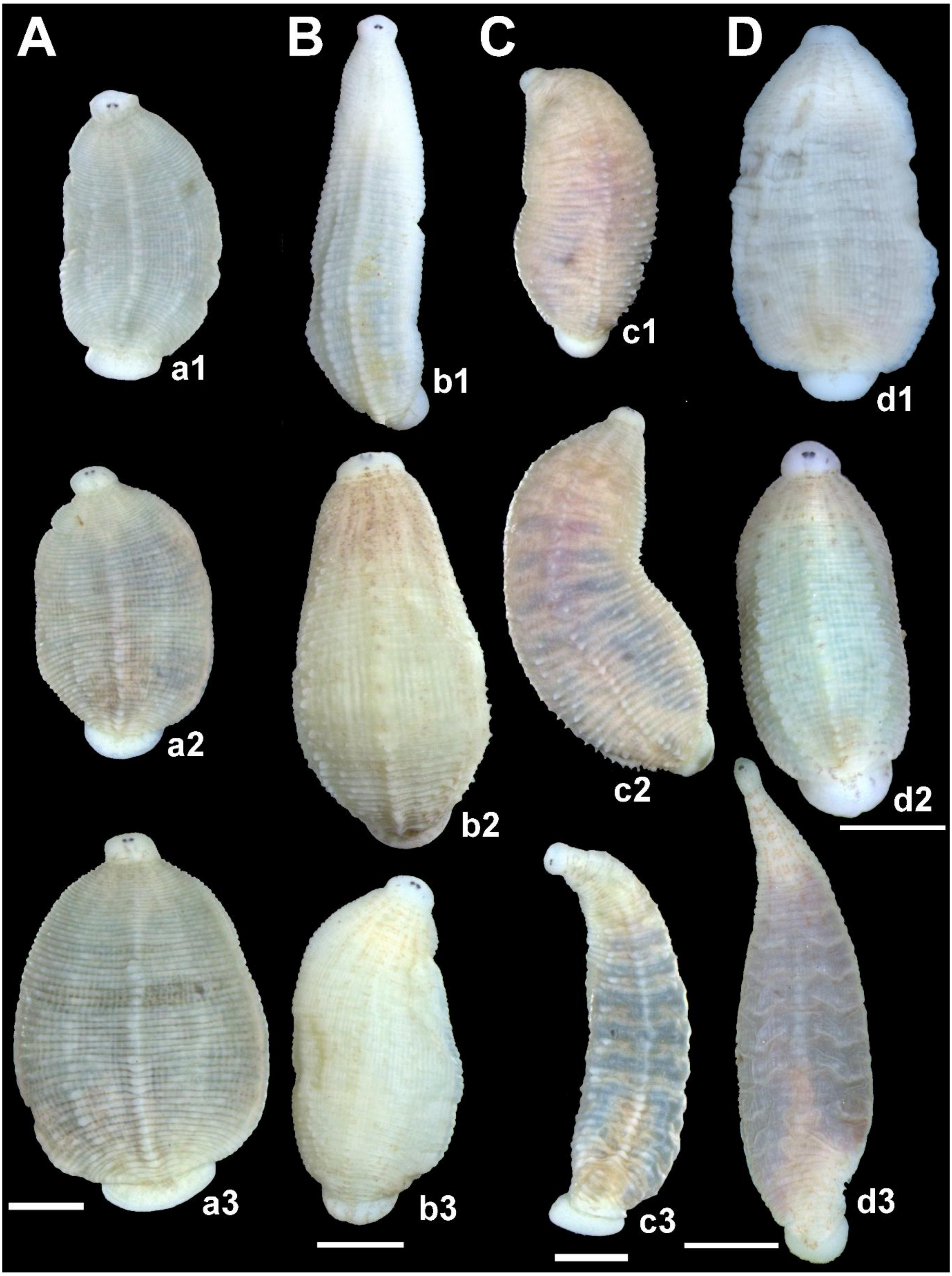

Supplementary Figure 7. Morphological variability of new *Batracobdelloides* species (dorsal view). (**A**) *B. hlaingbweensis* **sp. nov.** [paratypes RMBH Hir_0207 (a1), Hir_0214 (a2), and Hir_0215 (a3)]. (**B**) *B. indochinensis* **sp. nov.** [paratypes RMBH Hir_0053_1 (b1), Hir_0056_1 (b2), and specimen RMBH Hir_0066 (b3)]. (**C**) *B. yaukthwa* **sp. nov.** [paratypes RMBH Hir_0060_1 (c1), Hir_0060_1 (c2), and specimen RMBH Hir_0062 (c3)]. (**D**) *B. conchophylus* **sp. nov.** [paratype RMBH Hir_0055 (d1), specimen RMBH Hir_0055 (d2), and holotype RMBH Hir_0065_1-H (d3)]. Scale bars = 1 mm. (Photos: Anna L. Klass).

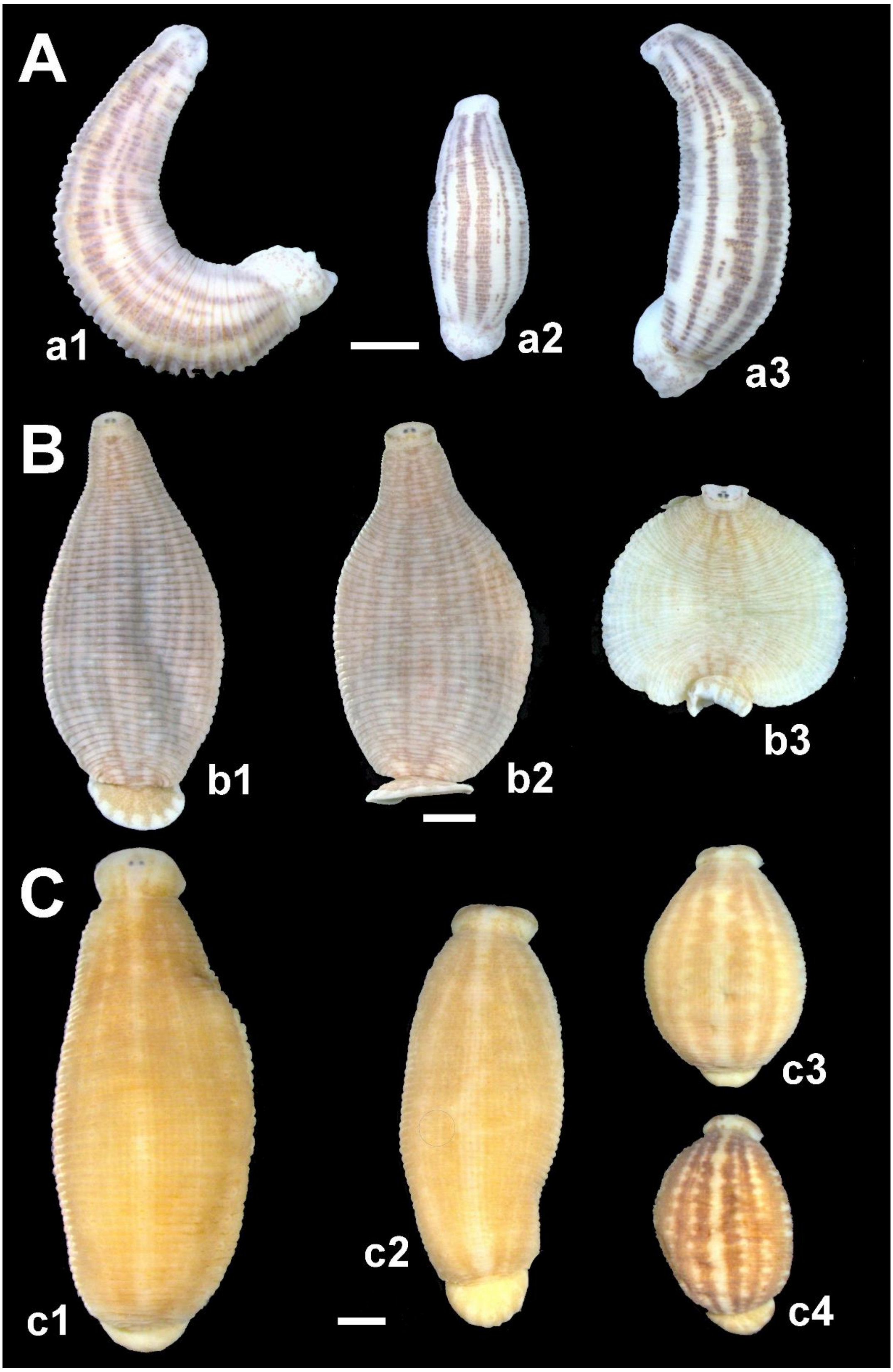

Supplementary Figure 8. Morphological variability of new *Hemiclepsis* species (dorsal view). (**A**) *H. khankiana* **sp. nov.** [sample RMBH Hir_0101 (a1-a3)]. (**B**) *H. myanmariana* **sp. nov.** [paratypes RMBH Hir_0048_1 (b1, b2) and RMBH Hir_0210 (b3)]. (**C**) *H. tumniniana* **sp. nov.** [paratypes RMBH Hir_0235 (c1, c2), RMBH Hir_0001 (c3), and RMBH Hir_0014 (c4)]. Scale bars = 1 mm. (Photos: Anna L. Klass).

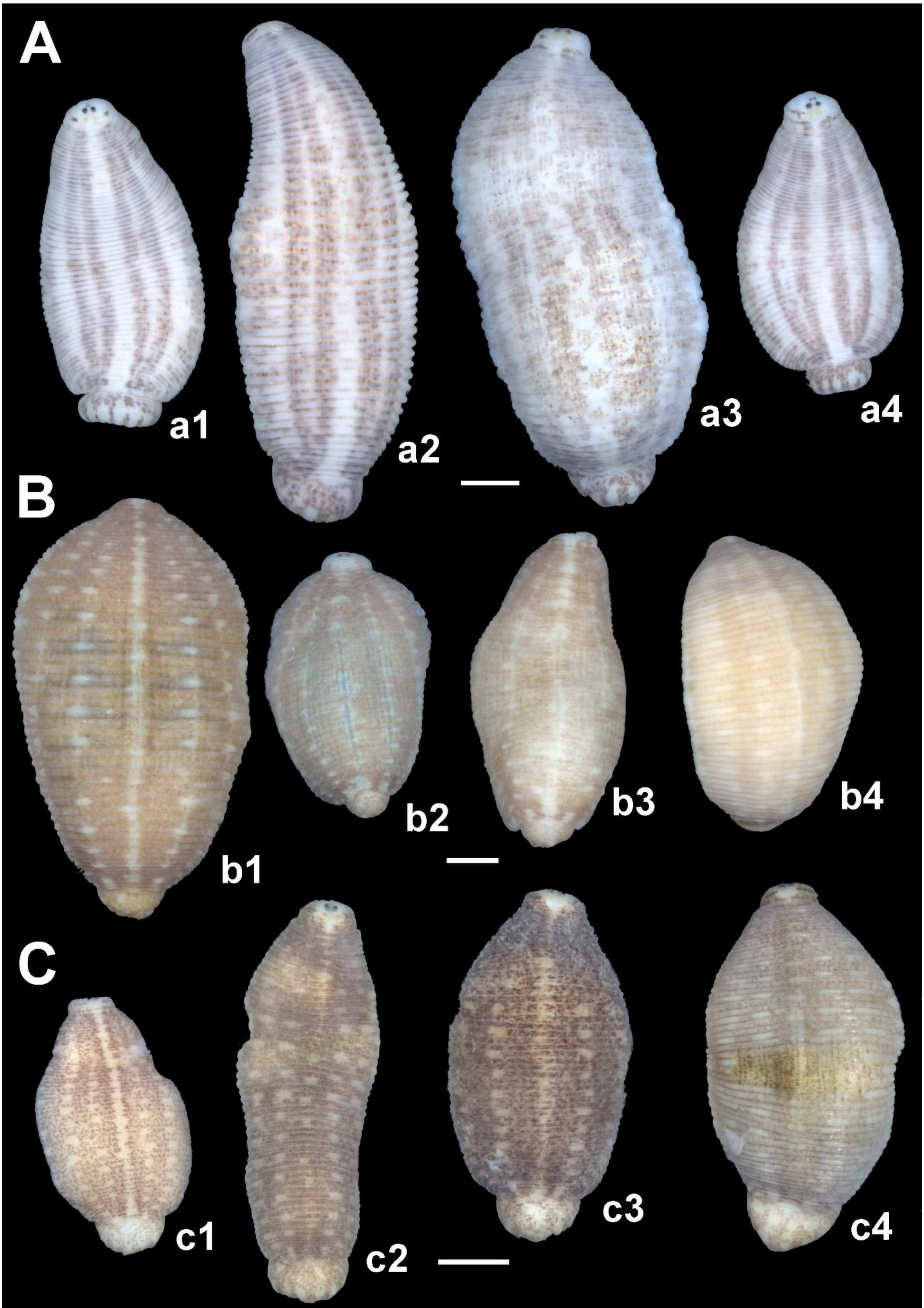

Supplementary Figure 9. Morphological variability of *Hemiclepsis kasmiana* **comb. rev.** (dorsal view). (**A**) Russian lineage [samples RMBH Hir_0128 (a1-a2) and Hir_0126 (a3-a4)]. (**B**) Korean lineage 1 [sample RMBH Hir_0113 (b1-a4)]. (**C**) Korean lineage 2 [samples RMBH Hir_0106 (c1), Hir_0118 (c2), and Hir_0119 (c3-c4)]. Scale bars = 1 mm. (Photos: Anna L. Klass).

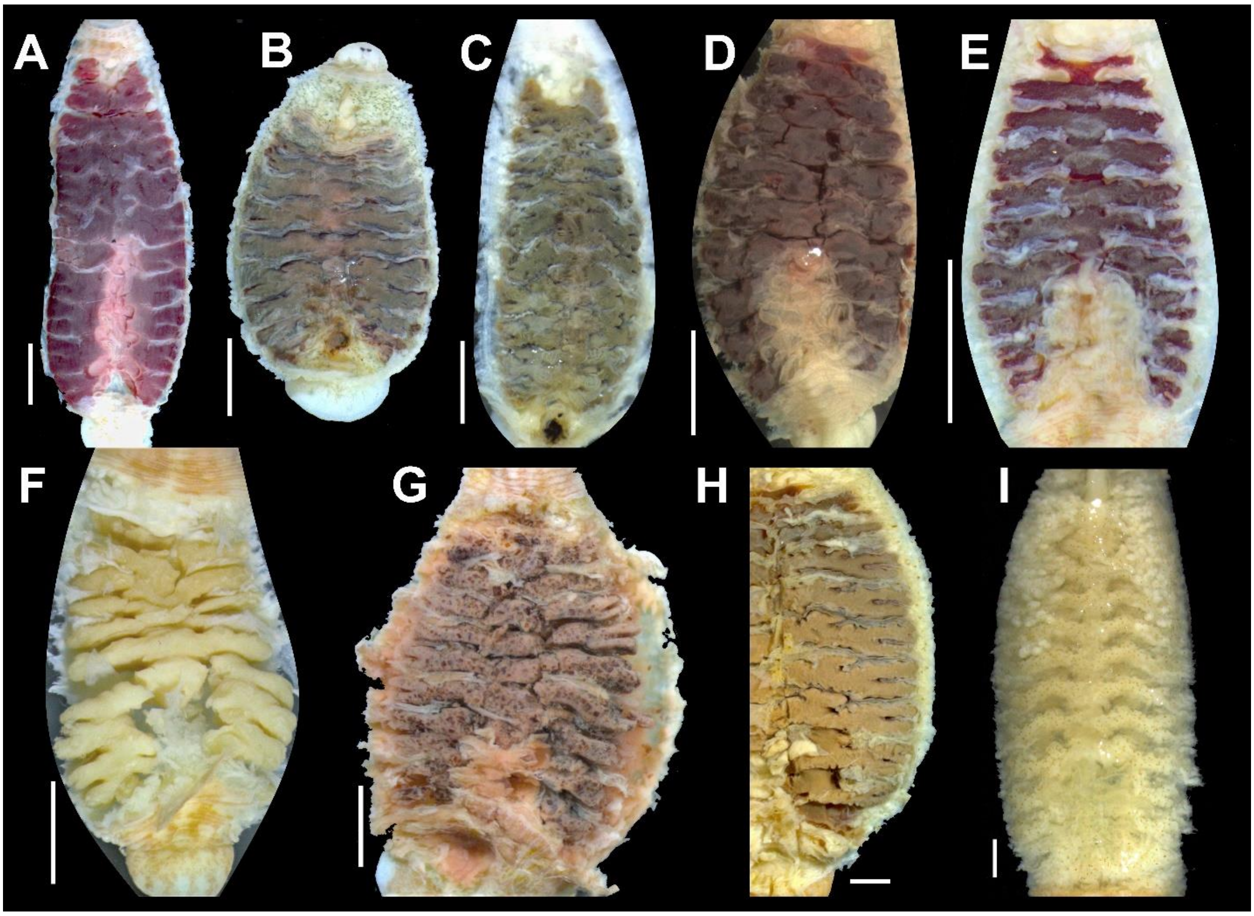

Supplementary Figure 10. Digestive system (dorsal view) of the new species, *Batracobdelloides tricarinatus*, and *Hemiclepsis kasmiana* **comb. rev.** (**A**) *Batracobdelloides conchophylus* **sp. nov.** [paratype RMBH Hir_65]. (**B**) *B. hlaingbweensis* **sp. nov.** [paratype RMBH Hir_207]. (**C**) *B. indochinensis* **sp. nov.** [paratype RMBH Hir_53]. (**D**) *B. yaukthwa* **sp. nov.** [paratype RMBH Hir_60]. (**E**) *B. tricarinatus* [specimen RMBH Hir_138]. (**F**) *Hemiclepsis kasmiana* **comb. rev.** [specimen RMBH Hir_116, South Korea]. (**G**) *H. myanmariana* **sp. nov.** [paratype RMBH Hir_48]. (**H**) *H. schrencki* **sp. nov.** [paratype RMBH Hir_88]. (**I**) *H. tumniniana* **sp. nov.** [paratype RMBH Hir_01]. Scale bars = 1 mm. (Photos: Anna L. Klass).

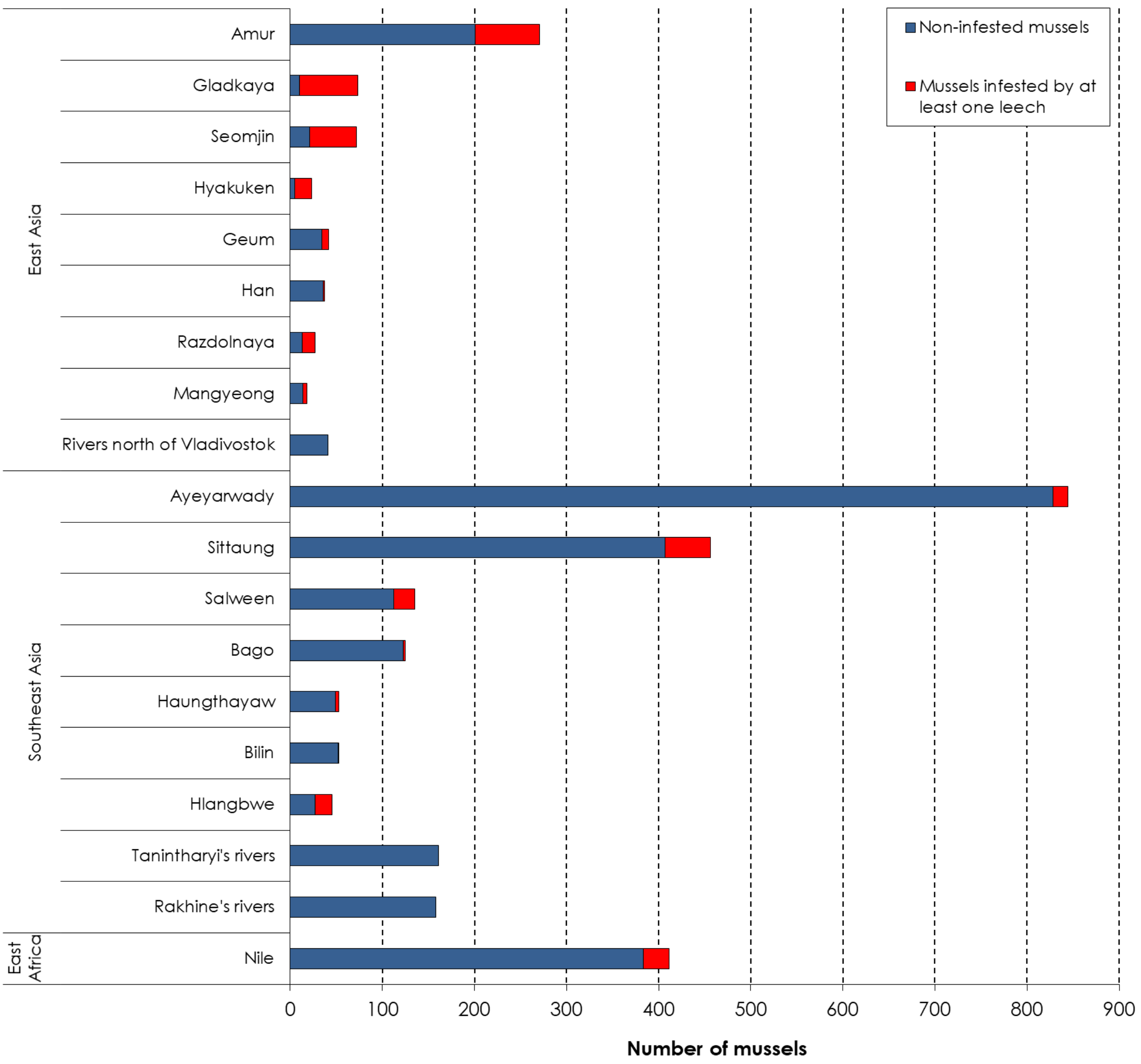

Supplementary Figure 11. Samples of freshwater mussels (Unionida: Unionidae, Margaritiferidae, and Iridinidae) collected from various freshwater basins in East Asia, Southeast Asia, and East Africa and their infestation by mussel-associated leeches (*N* = 3,045 mussels, primary data: Supplementary Dataset 1).

Supplementary Table 1. List of *COI* and *18S rRNA* gene sequences of the leech genera *Batracobdelloides* and *Hemiclepsis* (Glossiphoniidae) used in this study

Species	Locality	Sample ID*	NCBI's GenBank acc. nos.	
			COI	***18S rRNA***
***Batracobdelloides* Oosthuizen, 1986**				
B. amnicolus (Moore, 1958) **stat. rev.**	South Africa: Limpopo Basin, Maia's Dam, Gwalagwala tented camp, Hoedspruit	n/a	AY962457	AY962430
B. conchophylus **sp. nov.**	Myanmar: Lower Sittaung Basin, ox-bow lake near Taung Gyi village, 17.8807°N, 96.8313°E, from the mantle cavity of *Lamellidens generosus*	RMBH Hir_0065_1	**MN295408**	**MN312185**
B. conchophylus **sp. nov.**	Myanmar: Middle Sittaung River near Kanna village, 19.4857°N, 96.2750°E, from the mantle cavity of *Radiatula mouhoti*	RMBH Hir_0055	**MN295401**	n/a
B. hlaingbweensis **sp. nov.**	Myanmar: Hlaingbwe Basin, small stream, 17.0292°N, 97.8099°E, from the mantle cavity of *Pseudodon salwenianus*	RMBH Hir_0207	**MN295453**	n/a
B. hlaingbweensis **sp. nov.**	Myanmar: Hlaingbwe Basin, small stream, 17.0292°N, 97.8099°E, from the mantle cavity of *Pseudodon* sp.1	RMBH Hir_0209	**MN295455**	n/a
B. hlaingbweensis **sp. nov.**	Myanmar: Hlaingbwe Basin, small stream, 17.0483°N, 97.8194°E, from the mantle cavity of *Pseudodon salwenianus*	RMBH Hir_0215	**MN295458**	n/a
B. hlaingbweensis **sp. nov.**	Myanmar: Hlaingbwe Basin, small stream, 17.0483°N, 97.8194°E, from the mantle cavity of *Pseudodon salwenianus*	RMBH Hir_0214	**MN295457**	**MN595225**
B. indochinensis **sp. nov.**	Myanmar: Bago - Sittaung channel, 17.5818°N, 96.7733°E, from the mantle cavity of *Lamellidens generosus*	RMBH Hir_0053_1	**MN295399**	n/a
B. indochinensis **sp. nov.**	Myanmar: Middle Sittaung Basin, Mone Ding Dam outlet, 20.8099°N, 95.7242°E, from the mantle cavity of *Lamellidens savadiensis*	RMBH Hir_0056_1	**MN295402**	n/a
B. indochinensis **sp. nov.**	Myanmar: Salween Basin, fish pond near Demoso, 19.7289°N, 97.1167°E, from the mantle cavity of *Lamellidens ferrugineus*	RMBH Hir_0066	**MN295409**	**MN312186**
B. koreanus **sp. nov.**	South Korea: Mangyeong River, irrigation channel, 35.9165°N, 127.7135°E, from the mantle cavity of *Nodularia sinuata*	RMBH Hir_0104	**MN295424**	**MN312194**
B. tricarinatus (Blanchard, 1897)	Uganda: Uganda, Albert Nile near Pakwach town, 2.4579°N, 31.4964°E, from the mantle cavity of *Mutela* sp.	RMBH Hir_0140	**MN295446**	**MN312199**
B. tricarinatus (Blanchard, 1897)	Uganda: Uganda, Albert Nile near Pakwach town, 2.4579°N, 31.4964°E, from the mantle cavity of *Chambardia* sp.	RMBH Hir_0142	**MN295448**	n/a
B. tricarinatus (Blanchard, 1897)	Uganda: Uganda, Albert Nile	RMBH Hir_0143	**MN295449**	n/a

Species	Locality	Sample ID*	NCBI's GenBank acc. nos.	
			COI	*18S rRNA*
	near Pakwach town, 2.4579°N, 31.4964°E, from the mantle cavity of *Aspatharia* sp.			
B. tricarinatus (Blanchard, 1897)	Uganda: Uganda, Albert Nile near Pakwach town, 2.4579°N, 31.4964°E, from the mantle cavity of *Aspatharia* sp.	RMBH Hir_0144	**MN295450**	n/a
B. tricarinatus (Blanchard, 1897)	Uganda: Uganda, Albert Nile near Pakwach town, 2.4579°N, 31.4964°E, from the mantle cavity of *Aspatharia* sp.	RMBH Hir_0141	**MN295447**	n/a
B. tricarinatus (Blanchard, 1897)	Uganda: Uganda, Albert Nile near Pakwach town, 2.4579°N, 31.4964°E, from the mantle cavity of *Aspatharia* sp.	RMBH Hir_0139	**MN295445**	n/a
B. tricarinatus (Blanchard, 1897)	Uganda: Albert Nile Basin, Lake George, 0.0476°S, 30.1642°E, from the mantle cavity of *Coelatura aegyptiaca*	RMBH Hir_0138	**MN295444**	n/a
B. yaukthwa **sp. nov.**	Myanmar: Middle Sittaung Basin, Chain Stream, 17.9769°N, 96.7650°E, from the mantle cavity of *Trapezidens angustior*	RMBH Hir_0060_1	**MN295406**	**MN312184**
B. yaukthwa **sp. nov.**	Myanmar: Middle Sittaung Basin, Chain Stream, 17.9769°N, 96.7650°E, from the mantle cavity of *Indochinella pugio viridissima*	RMBH Hir_0062	**MN295407**	n/a
B. yaukthwa **sp. nov.**	Myanmar: Ayeyarwady Basin, Pathein River, 17.4567°N, 95.0086°E, from the mantle cavity of *Trapezidens dolichorhynchus*	RMBH Hir_0208	**MN295454**	n/a
***Hemiclepsis* Vejdovsky, 1884**				
H. kasmiana Oka, 1910 **comb. rev.** [Korean lineage 1]	South Korea: Geum River, 36.0708°N, 127.5891°E, beneath stone	RMBH Hir_0110_6	**MN295430**	**MN312196**
H. kasmiana Oka, 1910 **comb. rev.** [Korean lineage 1]	South Korea: Geum River, 36.0708°N, 127.5891°E, beneath stone	RMBH Hir_0110_6_2	**MN295431**	n/a
H. kasmiana Oka, 1910 **comb. rev.** [Korean lineage 1]	South Korea: Seomjin River, 35.4217°N, 127.2228°E, beneath stone	RMBH Hir_0113_2	**MN295432**	n/a
H. kasmiana Oka, 1910 **comb. rev.** [Korean lineage 1]	South Korea: Seomjin River, 35.4217°N, 127.2228°E, beneath stone	RMBH Hir_0113_2_2	**MN295433**	n/a
H. kasmiana Oka, 1910 **comb. rev.** [Korean lineage 2]	South Korea: Geum River, 35.9891°N, 127.5836°E, beneath stone	RMBH Hir_0103_4	**MN295423**	**MN312193**
H. kasmiana Oka, 1910 **comb. rev.** [Korean lineage 2]	South Korea: Geum River, 35.9891°N, 127.5836°E, from the mantle cavity of *Aculamprotula koreana*	RMBH Hir_0106	**MN295426**	n/a
H. kasmiana Oka, 1910 **comb. rev.** [Korean lineage 2]	South Korea: Geum River, 35.9891°N, 127.5836°E, from the mantle cavity of *Lamprotula gottschei*	RMBH Hir_0107	**MN295427**	n/a
H. kasmiana Oka, 1910 **comb. rev.** [Korean lineage 2]	South Korea: Seomjin River, 35.7010°N, 127.2845°E, from the mantle cavity of *Nodularia sinuata*	RMBH Hir_0116	**MN295434**	n/a

Species	Locality	Sample ID*	NCBI's GenBank acc. nos.	
			COI	*18S rRNA*
H. kasmiana Oka, 1910 **comb. rev.** [Korean lineage 2]	South Korea: Seomjin River, 35.7010°N, 127.2845°E, from the mantle cavity of *Sinanodonta lauta*	RMBH Hir_0118	**MN295435**	n/a
H. kasmiana Oka, 1910 **comb. rev.** [Korean lineage 2]	South Korea: Seomjin River, 35.4217°N, 127.2228°E, from the mantle cavity of *Nodularia douglasiae*	RMBH Hir_0119	**MN295436**	n/a
H. kasmiana Oka, 1910 **comb. rev.** [Korean lineage 2]	South Korea: Geum River, 35.9891°N, 127.5836°E, from the mantle cavity of *Nodularia* sp.	RMBH Hir_0124	**MN295442**	n/a
H. kasmiana Oka, 1910 **comb. rev.** [Korean lineage 2]	South Korea: Mangyeong River, irrigation channel, 35.9165°N, 127.7135°E, from the mantle cavity of *Sinanodonta lauta*	RMBH Hir_0120	**MN295437**	**MN312197**
H. kasmiana Oka, 1910 **comb. rev.** [Korean lineage 2]	South Korea: Mangyeong River, irrigation channel, 35.9165°N, 127.7135°E, from the mantle cavity of *Nodularia sinuata*	RMBH Hir_0121	**MN295438**	n/a
H. kasmiana Oka, 1910 **comb. rev.** [Russian lineage]	Russia: Khanka Lake basin, Melgunovka River, 44.5939°N, 132.1818°E, from the mantle cavity of *Nodularia douglasiae*	RMBH Hir_0123_1	**MN295440**	n/a
H. kasmiana Oka, 1910 **comb. rev.** [Russian lineage]	Russia: Khanka Lake basin, Komissarovka River mouth, 44.8255°N, 132.0456°E, from the mantle cavity of *Cristaria plicata*	RMBH Hir_0129_2	**MN295443**	**MN312198**
H. kasmiana Oka, 1910 **comb. rev.** [Russian lineage]	Russia: Khanka Lake basin, Melgunovka River, 44.5939°N, 132.1818°E, from the mantle cavity of *Buldowskia shadini*	RMBH Hir_0122	**MN295439**	n/a
H. kasmiana Oka, 1910 **comb. rev.** [Russian lineage]	Russia: Khanka Lake basin, Melgunovka River, 44.5939°N, 132.1818°E, from the mantle cavity of *Sinanodonta schrenckii*	RMBH Hir_0102	**MN295422**	n/a
H. kasmiana Oka, 1910 **comb. rev.** [Russian lineage]	Russia: Razdolnaya Basin, Komarovka River, 43.6392°N, 132.1614°E, from the mantle cavity of *Margaritifera dahurica*	RMBH Hir_0015_5	**MN295384**	n/a
H. kasmiana Oka, 1910 **comb. rev.** [Russian lineage]	Russia: Gladkaya River, 42.7065°N, 130.9084°E, from the mantle cavity of *Middendorffinaia mongolica*	RMBH Hir_0015_4_2	**MN295383**	n/a
H. kasmiana Oka, 1910 **comb. rev.** [Russian lineage]	Russia: Gladkaya River, 42.7065°N, 130.9084°E, from the mantle cavity of *Middendorffinaia mongolica*	RMBH Hir_0015_4_1	**MN295382**	n/a
H. kasmiana Oka, 1910 **comb. rev.** [Russian lineage]	Russia: Razdolnaya Basin, Soldatskoye Lake, 3.7747°N, 131.9406°E, from the mantle cavity of *Buldowskia suifunica*	RMBH Hir_0015_3_3	**MN295381**	n/a
H. kasmiana Oka, 1910 **comb. rev.** [Russian lineage]	Russia: Razdolnaya Basin, Soldatskoye Lake, 3.7747°N, 131.9406°E, from the mantle cavity of *Buldowskia suifunica*	RMBH Hir_0015_3_2	**MN295380**	**MN312174**
H. kasmiana Oka, 1910 **comb. rev.** [Russian lineage]	Russia: Razdolnaya Basin, Soldatskoye Lake, 3.7747°N, 131.9406°E, from the mantle cavity of *Buldowskia suifunica*	RMBH Hir_0015_3_1	**MN295379**	**MN312173**
H. kasmiana Oka, 1910 **comb.**	Russia: Gladkaya River,	RMBH Hir_0015_2	**MN295378**	n/a

Species	Locality	Sample ID*	NCBI's GenBank acc. nos.	
			COI	*18S rRNA*
**rev.** [Russian lineage]	42.7065°N, 130.9084°E, from the mantle cavity of *Buldowskia suifunica*			
H. kasmiana Oka, 1910 **comb. rev.** [Russian lineage]	Russia: Gladkaya River, 42.7065°N, 130.9084°E, from the mantle cavity of *Sinanodonta lauta*	RMBH Hir_0015_1_2	**MN295377**	**MN312172**
H. kasmiana Oka, 1910 **comb. rev.** [Russian lineage]	Russia: Gladkaya River, 42.7065°N, 130.9084°E, from the mantle cavity of *Sinanodonta lauta*	RMBH Hir_0015_1_1	**MN295376**	**MN312171**
H. kasmiana Oka, 1910 **comb. rev.** [Russian lineage]	Russia: Khanka Lake basin, Komissarovka River mouth, 44.8255°N, 132.0456°E, from the mantle cavity of *Nodularia douglasiae*	RMBH Hir_0105	**MN295425**	**MN312195**
H. khankiana **sp. nov.**	Russia: Khanka Lake basin, Melgunovka River, 44.5804°N, 132.0803°E, from the mantle cavity of *Nodularia douglasiae*	RMBH Hir_0101	**MN295420**	**MN312192**
H. khankiana **sp. nov.**	Russia: Khanka Lake basin, Melgunovka River, 44.5804°N, 132.0803°E, from the mantle cavity of *Nodularia douglasiae*	RMBH Hir_0101_2	**MN295421**	n/a
H. khankiana **sp. nov.**	Russia: Khanka Lake basin, Melgunovka River, 44.5939°N, 132.1818°E, from the mantle cavity of *Nodularia douglasiae*	RMBH Hir_0123_2	**MN295441**	n/a
H. marginata (O. F. Müller, 1774)	Kazakhstan: Irtysh - Ob' River basin, Lower Taynty Reservoir, 49.4418°N, 83.0585°E	RMBH Hir_00151_2	**MN295451**	n/a
H. marginata (O. F. Müller, 1774)	Russia: Moscow Region, Volga River basin, a reservoir near Pokrovskoye village, 55.3259°N, 37.2066°E	RMBH Hir_0020_1	**MN295385**	**MN312175**
H. marginata (O. F. Müller, 1774)	Russia: Moscow Region, Volga River basin, a reservoir on the Lopastnya River, 55.2555°N, 37.1744°E	RMBH Hir_0024_1	**MN295388**	**MN312178**
H. marginata (O. F. Müller, 1774)	Russia: Moscow Region, Volga River basin, a pond near Starosyrovo village, 55.4909°N, 37.5275°E	RMBH Hir_0025_3	**MN295390**	**MN312179**
H. marginata (O. F. Müller, 1774)	Russia: Moscow Region, Volga River basin, a stream near Vaulino village, 56.5333°N, 38.2438°E	RMBH Hir_0160_2	**MN295452**	n/a
H. myanmariana **sp. nov.**	Myanmar: Lower Sittaung Basin: Pangaing Stream, 17.7080°N, 96.7155°E, from the mantle cavity of *Lamellidens generosus*	RMBH Hir_0054	**MN295400**	**MN312183**
H. myanmariana **sp. nov.**	Myanmar: Middle Sittaung Basin: Mone Ding Dam outlet, 20.8099°N, 95.7242°E, from the mantle cavity of *Lamellidens savadiensis*	RMBH Hir_0056_2	**MN295403**	n/a
H. myanmariana **sp. nov.**	Myanmar: Salween Basin, Nadi Lake, 20.6858°N, 96.9316°E, from the mantle cavity of *Lamellidens savadiensis*	RMBH Hir_0048_1	**MN295394**	**MN312180**

Species	Locality	Sample ID*	NCBI's GenBank acc. nos.	
			COI	*18S rRNA*
H. myanmariana **sp. nov.**	Myanmar: Bilin Basin: Shwe Laung Lake near Pyintha village, 17.4395°N, 97.2457°E, from the mantle cavity of *Lamellidens generosus*	RMBH Hir_0052	**MN295398**	**MN312182**
H. myanmariana **sp. nov.**	Myanmar: Ayeyarwady Basin, ox-bow lake near Ta Naung Taig village, 21.4064°N, 95.3399°E, from the mantle cavity of *Lamellidens savadiensis*	RMBH Hir_0051	**MN295397**	**MN312181**
H. myanmariana **sp. nov.**	Myanmar: Ayeyarwady Basin, Nga Wun River near Pyay town, 18.8624°N, 95.2822°E, from the mantle cavity of *Lamellidens savadiensis*	RMBH Hir_0211	**MN295456**	n/a
H. schrencki **sp. nov.**	Russia: Primorye Region, Ussuri Basin, Muravievka River, 43.7703°N, 133.2611°E	RMBH Hir_0088_1	**MN295415**	**MN312189**
H. schrencki **sp. nov.**	Russia: Primorye Region, Partizanskaya River, 43.0585°N, 133.1540°E	RMBH Hir_0091_1	**MN295416**	**MN312190**
H. tumniniana **sp. nov.**	Russia: Khabarovsk Region, Tumnin River, 49.9451°N, 139.9181°E	RMBH Hir_0067	**MN295410**	n/a
H. tumniniana **sp. nov.**	Russia: Khabarovsk Region, Tumnin River, 50.0001°N, 139.9175°E	RMBH Hem_1_2	**MN295371**	**MN312166**
H. tumniniana **sp. nov.**	Russia: Khabarovsk Region, Tumnin River, 50.0001°N, 139.9175°E	RMBH Hem_2_1	**MN295373**	**MN312168**
H. tumniniana **sp. nov.**	Russia: Khabarovsk Region, Tumnin River, 50.0001°N, 139.9175°E	RMBH Hem_2_2	**MN295374**	**MN312169**
H. tumniniana **sp. nov.**	Russia: Khabarovsk Region, Tumnin River, 50.0001°N, 139.9175°E	RMBH Hir_0093	**MN295417**	**MN312191**
H. sp.1	South Korea	SOKN015	KF966547	n/a

*RMBH – Russian Museum of Biodiversity Hotspots, Federal Center for Integrated Arctic Research, Russian Academy of Sciences, Arkhangelsk, Russia. New sequences generated in this study are in bold. n/a – not available.

Supplementary Table 2. List of *COI* and *18S rRNA* gene sequences used in phylogenetic reconstruction of the Hirudinea

Genus	Species	Haplotype Code	Region	*COI*	*18S rRNA*
**In-group taxa**					
Glossiphoniidae: *Batracobdelloides* Oosthuizen, 1986	*B. amnicolus* (Moore, 1958) **stat. rev.**	BatAmn	Africa	AY962457	AY962430
	B. conchophylus **sp. nov.**	BatCon	Southeast Asia	**MN295408**	**MN312185**
	B. hlaingbweensis **sp. nov.**	BatHla	Southeast Asia	**MN295453**	**MN595225**
	B. indochinensis **sp. nov.**	BatIn1	Southeast Asia	**MN295399**	n/a
	B. indochinensis **sp. nov.**	BatIn2	Southeast Asia	**MN295402**	n/a
	B. indochinensis **sp. nov.**	BatIn3	Southeast Asia	**MN295409**	**MN312186**
	B. koreanus **sp. nov.**	BatKor	East Asia	**MN295424**	**MN312194**
	B. tricarinatus (Blanchard, 1897)	BatTr1	Africa	**MN295444**	n/a
	B. tricarinatus (Blanchard, 1897)	BatTr2	Africa	**MN295445**	n/a
	B. tricarinatus (Blanchard, 1897)	BatTr3	Africa	**MN295446**	**MN312199**
	B. yaukthwa **sp. nov.**	BatYau	Southeast Asia	**MN295406**	**MN312184**
Glossiphoniidae: *Hemiclepsis* Vejdovsky, 1884	*H. kasmiana* Oka, 1910 **comb. rev.** [Korean lineage 1]	HemKa2_1	East Asia	**MN295431**	n/a
	H. kasmiana Oka, 1910 **comb. rev.** [Korean lineage 1]	HemKa2_2	East Asia	**MN295432**	n/a
	H. kasmiana Oka, 1910 **comb. rev.** [Korean lineage 2]	HemKa3_1	East Asia	**MN295437**	**MN312197**
	H. kasmiana Oka, 1910 **comb. rev.** [Korean lineage 2]	HemKa3_2	East Asia	**MN295426**	n/a
	H. kasmiana Oka, 1910 **comb. rev.** [Russian lineage]	HemKa1_1	East Asia	**MN295376**	**MN312171**
	H. kasmiana Oka, 1910 **comb. rev.** [Russian lineage]	HemKa1_2	East Asia	**MN295425**	**MN312195**
	H. khankiana **sp. nov.**	HemKha	East Asia	**MN295420**	**MN312192**
	H. myanmariana **sp. nov.**	HemMy1	Southeast Asia	**MN295398**	**MN312182**
	H. myanmariana **sp. nov.**	HemMy2	Southeast Asia	**MN295394**	**MN312180**
	H. myanmariana **sp. nov.**	HemMy3	Southeast Asia	**MN295403**	n/a
	H. marginata (O. F. Müller, 1774)	HemMar	Europe	**MN295388**	**MN312178**
	H. schrencki **sp. nov.**	HemSc1	East Asia	**MN295415**	**MN312189**
	H. schrencki **sp. nov.**	HemSc2	East Asia	**MN295416**	**MN312190**
	H. tumniniana **sp. nov.**	HemTum	East Asia	**MN295417**	**MN312191**
	H. sp.1 Korea	HemSp1	East Asia	KF966547	n/a
Glossiphoniidae: *Alboglossiphonia* Lukin, 1976	*A. heteroclita* (Linnaeus, 1761)	AlbHet	North America	AF116016	AF115983
	A. papillosa (Braun, 1805)	AlbPap	East Asia	MH286267	MH286273
	A. quadrata (Moore, 1924)	AlbQua	Africa	AY962455	AY962412
	A. lata (Oka, 1910)	AlbLa1	East Asia	AY962454	AY962411
	A. lata (Oka, 1910)	AlbLa2	East Asia	**MN295414**	**MN312188**
	A. sp.1 Myanmar	AlbSp1	Southeast Asia	**MN295404**	n/a

Genus	Species	Haplotype Code	Region	*COI*	*18S rRNA*
Glossiphoniidae: *Glossiphonia* Johnson, 1816	*G. baicalensis* (Stschegolew, 1922)	GloBai	East Asia	AY047329	AY962425
	G. complanata (Linnaeus, 1758)	GloCom	Europe	**MN295386**	**MN312176**
	G. verrucata (F. Müller, 1844)	GloVer	Europe	AY962459	AY962432
	G. cf. nebulosa Kalbe, 1964	GloNeb	Europe	**MN295412**	n/a
	G. sp.1 Siberia	GloSp1	East Asia	**MN295411**	n/a
	G. sp.2 Korea	GloSp2	East Asia	**MN295429**	n/a
	G. sp.3 Tumnin River	GloSp3	East Asia	**MN295375**	**MN312170**
	G. sp.4 Moscow	GloCo1	Europe	AY962458	AY962431
	G. sp.4 Moscow	GloCo2	Europe	**MN295418**	n/a
	G. sp.4 Moscow	GloCo3	Europe	**MN295391**	n/a
Glossiphoniidae: *Haementeria* De Filippi, 1849	*H. ghilianii* De Filippi, 1849	HaeGhi	South America	AF116017	AF115985
	H. gracilis (Weyenbergh, 1883)	HaeGra	South America	AF329034	AF115984
	H. lutzi Pinto, 1920	HaeLut	South America	AF329033	AY962433
	H. molesta Cordero, 1937	HaeMol	South America	AY047325	AY962422
	H. tuberculifera (Grube, 1871)	HaeTub	South America	AF329036	AY962415
Glossiphoniidae: *Helobdella* Blanchard 1896	*H. elongata* Castle, 1900	HelElo	North America	AF329045	AY962419
	H. fusca (Castle, 1900)	HelFus	North America	AF329038	AY962414
	H. nununununojensis Siddall, 2001	HelNun	South America	AF329048	AY962426
	H. papillata (Moore, 1952)	HelPap	North America	AF329043	AY962417
	H. paranensis (Oka, 1930)	HelPar	South America	AF116019	AF115987
	H. pichipanan Siddall & Borda, 2004	HelPic	South America	AY962456	AY962429
	H. ringueleti Siddall, 2001	HelRin	South America	MF067107	AY962428
	H. sorojchi Siddall, 2001	HelSor	South America	AF329050	AY962427
	H. stagnalis (Linnaeus, 1758)	HelSt1	Europe	**MN295393**	n/a
	H. stagnalis (Linnaeus, 1758)	HelSt2	Europe	**MN295387**	**MN312177**
	H. cf. *stagnalis* (Linnaeus, 1758)	HelSt3	Europe	**MN295389**	n/a
	H. transversa Sawyer, 1972	HelTra	North America	AF329044	AY962418
	H. triserialis (E. Blanchard, 1849)	HelTri	South America	AF329054	AY962435
	H. sp.3 Korea	HelSp3	East Asia	**MN295428**	n/a
	H. sp. 4 Tumnin River	HelSp4	East Asia	**MN295372**	**MN312167**
Glossiphoniidae: *Placobdella* Blanchard, 1893	*P. biannulata* (Moore, 1900)	PlaBia	North America	AF116021	AF115989
	P. costata (F. Müller, 1846)	PlaCos	Europe	AY962461	AY962436
	P. montifera (Moore, 1906)	PlaMon	North America	AY047323	AY962420
	P. multilineata Moore, 1953	PlaMul	North America	AY962464	AY962439
	P. papillifera (Verrill, 1872)	PlaPap	North America	AY047324	AY962421
	P. parasitica (Say, 1824)	PlaPar	North America	AF003261	AF115990
	P. pediculata Hemingway, 1908	PlaPed	North America	AY047327	AY962423
	P. picta (Verrill, 1872)	PlaPic	North America	AF116020	AF115988

Genus	Species	Haplotype Code	Region	*COI*	*18S rRNA*
	P. transluscens (Sawyer & Shelley, 1976)	PlaTra	North America	AY047328	AY962424
	P. sp.1 Southern European Russia	PlaSp1	Europe	**MN295392**	n/a
Piscicolidae: gen. indet.	Piscicolidae gen. & sp. indet.	PisGen	East Asia	**MN295413**	**MN312187**
Piscicolidae: *Piscicola* Blainville, 1818	*P. geometra* (Linnaeus, 1761)	PisGeo	Europe	AY336014	AF099946
Piscicolidae: *Branchellion* Savigny, 1822	*B. torpedinis* Savigny, 1822	BraTor	North America	AF003265	AF115993
Piscicolidae: *Calliobdella* van Beneden & Hesse, 1863	*C. vivida* (Verrill, 1872)	CalViv	North America	AF003260	AF115992
Piscicolidae: *Myzobdella* Leidy, 1851	*M. lugubris* Leidy, 1851	MyzLug	North America	AF003269	AF115994
Piscicolidae: *Stibarobdella* Leigh-Sharpe, 1925	*S. macrothela* (Schmarda, 1861)	StiMac	North America	AF116022	AF115996
Erpobdellidae: *Erpobdella* Lamarck, 1818	*E. japonica* (Pawlowski, 1952)	ErpJap	East Asia	AF116026	AF116000
	E. monostriata (Lindenfeld & Pietruszynski, 1890)	ErpMon	Europe	**MN295419**	n/a
	E. punctata (Leidy, 1870)	ErpPun	North America	AF003275	AF116002
	E. testacea (Savigny, 1822)	ErpTes	Europe	AF116027	AF116003
Erpobdellidae: *Dina* Blanchard, 1892	*D. dubia* Moore & Meyer, 1951	DinDub	North America	AF116023	AF115997
Erpobdellidae: *Mooreobdella* Pawlowski, 1955	*M. melanostoma* Sawyer & Shelley, 1976	MooMel	North America	AF116025	AF115999
	M. bucera Moore, 1949	MooBuc	North America	AF116024	AF115998
Erpobdellidae: *Nephelopsis* Verrill, 1872	*N. obscura* Verrill, 1872	NepObs	North America	AF003273	AF116004
Gastrostomobdellidae: *Gastrostomobdella* Moore, 1929	*G. extenta* Nakano and Jeratthitikul, 2018	GasExt	Southeast Asia	LC274553	LC274519
	G. ampunganensis Nakano, 2018	GasAmp	Southeast Asia	LC274551	LC274517
Salifidae: *Barbronia* Johansson, 1918	*B.* sp.1	BarSp1	Southeast Asia	**MN295405**	n/a
	B. weberi (Blanchard, 1897)	BarWeb	Southeast Asia	AY786456	AY786461
Salifidae: *Salifa* Blanchard, 1897	*S. motokawai* Nakano & Nguyen, 2015	SalMot	Southeast Asia	LC029431	LC029434
Salifidae: Gen indet.	Gen. & sp. indet.1	SalGe1	Southeast Asia	**MN295395**	n/a
	Gen. & sp. indet.2	SalGe2	Southeast Asia	**MN295396**	n/a
Orobdellidae: *Orobdella* Oka, 1895	*O. koikei* Nakano, 2012	OroKoi	East Asia	AB679688	AB698883
	O. esulcata Nakano, 2010	OroEsu	East Asia	AB675020	AB663655
Ozobranchidae: *Ozobranchus* Quatrefages, 1852	*O. branchiatus* (Menzies, 1791)	OzoBra	North America	KF728213	KF728214
	O. margoi (Apáthy, 1890)	OzoMar	North America	KJ451407	KF728217
Hirudinidae: *Hirudo* Linnaeus, 1758	*H. orientalis* Utevsky & Trontelj, 2005	HirOri	Central Asia	GQ368750	GQ368792
	H. troctina Johnson, 1816	HirTro	Africa	GQ368751	GQ368793
Hirudinidae: *Aliolimnatis* Richardson, 1972	*A. africana* (Blanchard, 1897)	AliAfr	Africa	AY425451	AY425469
	A. michaelseni (Augener, 1936)	AliMic	Africa	AF116029	AF116010
Hirudinidae: *Macrobdella* Verrill, 1872	*M. decora* (Say, 1824)	MacDec	North America	AF003271	AF116007
Hirudinidae: *Poecilobdella* Blanchard, 1893	*P. nanjingensis* Yang, 1996	PoeNan	East Asia	LC145739	LC145737

Genus	Species	Haplotype Code	Region	*COI*	*18S rRNA*
Hirudinidae: *Hirudinaria* Whitman, 1886	*H. javanica* (Wahlberg, 1856)	HirJav	Southeast Asia	GQ368745	GQ368787
Hirudinidae: *Goddardobdella* Richardson, 1969	*G. elegans* (Grube, 1867)	GodEle	Australia	GQ368743	GQ368785
Haemadipsidae: *Chtonobdella* Grube, 1866	*C. bilineata* (Richardson, 1975)	ChtBil	Australia	AF003267	AF116006
Haemadipsidae: *Haemadipsa* Tennent, 1859	*H. sylvestris* Blanchard, 1894	HaeSyl	Southeast Asia	AF003266	AF116005
Haemopidae: *Haemopis* Savigny, 1822	*H. lateromaculata* Mathers 1963	HaeLat	North America	AF116028	AF116009
	H. marmorata (Say 1824)	HaeMar	North America	AF003270	AF116008
**Outgroup taxa (Oligochaeta)**					
Lumbricidae: *Lumbricus* Linnaeus, 1758	*L. terrestris* Linnaeus, 1758	LumTer	N/A	HQ691222	HQ691211
Lumbricidae: *Aporrectodea* Orley, 1885	**A. trapezoides* (Dugés, 1828)	ApoTra	N/A	EF077601	HQ621897
Hormogastridae: *Hormogaster* Rosa, 1887	*H. arenicola* Qiu & Bouché, 1998	HorAre	N/A	HQ621972	HQ621898
	H. redii Rosa, 1887	HorRed	N/A	HQ621976	HQ621895
Naididae: *Tubifex* Lamarck, 1816	**T. tubifex* (Müller, 1774)	TubTub	N/A	HM138035	AF397152

New sequences generated in this study are in bold. n/a – not available. *Chimeric sequence.

Supplementary Table 3. Summary of molecular diagnoses of the new leech species in the genera *Batracobdelloides* and *Hemiclepsis* (Glossiphoniidae)

**Species**	**Mean *COI p*-distance (±bootstrap standard error estimate) from the nearest neighbor species, %**	**The nearest neighbor of a new taxon based on the mean *COI p*-distance**	**Fixed nucleotide differences based on the sequence alignment of congeners**	
			COI	***18S rRNA***
Batracobdelloides conchophylus **sp. nov.**	4.1±0.7	*B. indochinensis* **sp. nov.**	134T, 377T, 455A, 470G, 500C, 548C, 575C, 614T, 642C	Absent
B. hlaingbweensis **sp. nov.**	3.8±0.7	*B. yaukthwa* **sp. nov.**	128C, 167G, 191G, 413A, 437C	Absent
B. indochinensis **sp. nov.**	4.1±0.7	*B. conchophylus* **sp. nov.**	8G, 44T, 198C, 338T, 461G, 602C, 650G	Absent
B. yaukthwa **sp. nov.**	3.8±0.7	*B. hlaingbweensis* **sp. nov.**	269T, 299C, 359C, 402C, 404C, 501C, 554C, 599C, 626G	Absent
B. koreanus **sp. nov.**	5.1±0.9	*B. hlaingbweensis* **sp. nov.**	86C, 179C, 188C, 206A, 209C, 371A, 413C, 429C, 460C, 524C	154T
Hemiclepsis khankiana **sp. nov.**	3.8±0.7	*H. kasmiana* **comb. rev.**	320G, 408C, 456A, 458T, 641A	Absent
H. myanmariana **sp. nov.**	7.1±1.0	*H. khankiana* **sp. nov.**	5T, 374T/C, 392A, 429C, 452A, 455A, 494A/G, 549C, 558C, 566T, 623T	769T, 798A, 1364T, 1693T
H. schrencki **sp. nov.**	9.1±1.0	*H. khankiana* **sp. nov.**	23G, 266T, 269T, 290G, 296C, 338C, 380C, 398C, 434G, 495A, 530C, 569T, 581C, 605T, 623G	Absent
H. tumniniana **sp. nov.**	9.5±1.1	*H. kasmiana* **comb. rev.**	89T, 95C, 110G, 182G, 194C, 218G, 227C, 260C, 281G, 288C, 314G, 317G, 320C, 341C, 368C, 387C, 389G, 410G, 476G, 479A, 494C, 512G, 579T, 611C, 617C, 632T, 654C	Absent

Supplementary Table 4. Morphological description of the new leech species in the genera *Batracobdelloides* and *Hemiclepsis* (Glossiphoniidae) based on ethanol-preserved specimens

Species	Coloration (fixed animals)	Papillation	Total number of annuli	Somites	Eyes	Gonopores	Reproductive system	Digestive system
Batracobdelloides conchophylus **sp. nov.**	Dorsum white, with seven rows of unclear light brown spots in the anterior third of the animal. Sometimes a few light brown spots on the posterior sucker. Venter white	Three longitudinal rows of conical tubercles with rounded apex	68	I–II joined to form a preocular lip, III–IV biannulate, V–XXIV triannulate, XXV–XXVII uniannulate	Two pairs: the first pair strongly reduced and joined with the second pair; the second pair large, angulate, merging, located on III somite	XI/XII (male) and in XII a1/a2 (female)	Six pairs of spherical testisacs arranged inter-segmentally in XIII/XVIII. Ejaculatory channels twisted, moderately long, located at much lower lever by the sides of the atrium. Ovisacs large, elongated, convoluted thin-walled structures, arranged as loops. Atrium spherical, slightly elongated, the atrial cornua sharply spun up	Crop caeca with 7 weakly branched pairs, 7th pair has four blind processes. Intestine has 4 pairs of short processes. Salivary glands diffuse
B. hlaingbweensis **sp. nov.**	Dorsum greenish or white, without clear markings. Posterior sucker and venter white	One central row of triangular, flattened tubercles with rounded apex. Similar lateral tubercles broadly scattered	68	I–II joined to form a preocular lip, III–IV biannulate, V–XXIV triannulate, XXV–XXVII uniannulate	Two pairs: the first pair almost completely reduced up to an unclear shading and located between the second pair; the second pair of moderate size, separate, angulate, located on III somite	XI/XII (male) and in XII a1/a2 (female)	Six pairs of spherical testisacs arranged inter-segmentally in XIII/XVIII. Ejaculatory channels twisted, moderately long, located at much lower lever by the sides of the atrium. Ovisacs large, elongated, convoluted thin-walled structures, arranged as loops. Atrium small, narrow, the atrial cornua twisted down	Crop caeca with 7 weakly branched pairs, 7th pair has four blind processes. Intestine has 4 pairs of short processes. Salivary glands diffuse
B. indochinensis **sp. nov.**	Dorsum white or yellowish, sometimes with diffuse brownish dots scattered throughout the anterior third of the animal. Posterior sucker white, sometimes with unclear radial brown bands. Venter white	Three longitudinal rows of conical tubercles with rounded apex	68	I–II joined to form a preocular lip, III–IV biannulate, V–XXIV triannulate, XXV–XXVII uniannulate	Two pairs: the first pair almost completely reduced up to an unclear shading and located between the second pair; the second pair of moderate size, separate, rounded, located on III somite	XI/XII (male) and in XII a1/a2 (female)	Six pairs of spherical testisacs arranged inter-segmentally in XIII/XVIII. Ejaculatory channels twisted, moderately long, located at much lower lever by the sides of the atrium. Ovisacs large, elongated, convoluted thin-walled structures, arranged as loops. Atrium small, spherical, slightly elongated, the atrial cornua twisted anteriorly	Crop caeca with 7 weakly branched pairs, 7th pair has four blind processes. Intestine has 4 pairs of short processes. Salivary glands diffuse
B. yaukthwa **sp. nov.**	Dorsum reddish or	One central	68	I–II joined to form	Two pairs: the first	XI/XII	Six pairs of spherical testisacs	Crop caeca with

Species	Coloration (fixed animals)	Papillation	Total number of annuli	Somites	Eyes	Gonopores	Reproductive system	Digestive system
	brownish. Posterior sucker white, without markings. Venter reddish	row of spike-like tubercles. Separate spike-like tubercles scattered laterally		a preocular lip, III–IV biannulate, V–XXIV triannulate, XXV–XXVII uniannulate	pair completely reduced (not visible); the second pair of moderate size, separate, ovate, located on III somite	(male) and in XII a1/a2 (female)	arranged inter-segmentally in XIII/XVIII. Ejaculatory channels twisted, moderately long, located at much lower lever by the sides of the atrium. Ovisacs large, elongated, convoluted thin-walled structures, arranged as loops. Atrium spherical, the atrial cornua twisted	7 weakly branched pairs, 7th pair has four blind processes. Intestine has 4 pairs of short processes. Salivary glands diffuse
B. koreanus **sp. nov.**	Dorsum light yellow, with seven longitudinal brown stripes. Posterior sucker with radial brownish bands. Venter yellowish	Three rows of weakly developed, almost invisible, rounded tubercles	68	I–II joined to form a preocular lip, III–IV biannulate, V–XXIV triannulate, XXV–XXVII uniannulate	Two pairs: the first pair strongly reduced up to small dots; the second pair large, rounded, located on III somite	XI/XII (male) and in XII a1/a2 (female)	Six pairs of spherical testisacs arranged inter-segmentally in XIII/XVIII. Ejaculatory channels twisted, moderately long, located at much lower lever by the sides of the atrium. Ovisacs large, elongated, convoluted thin-walled structures, arranged as loops. Atrium small, spherical, slightly elongated, the atrial cornua directed laterally	Crop caeca with 7 weakly branched pairs, 7th pair has four blind processes. Intestine has 4 pairs of short processes. Salivary glands diffuse
Hemiclepsis khankiana **sp. nov.**	Dorsum yellowish or whitish, with six longitudinal broad, smooth brown stripes. Posterior sucker without bands, but with dense, diffuse brown dots	Absent	72	I–II uniannulate, III biannulate, IV–XXIV triannulate, XXV biannulate, XXVI–XXVII uniannulate. The head region (I–V) is clearly separated from the body	Two pairs: the first pair strongly reduced up to an unclear shading and located on the border of II and III somites; the second pair large, separate, cup-like, and located in III somite	XI/XII (male) and in XII a1/a2 (female)	Six pairs of spheric testisacs arranged inter-segmentally on XIII/XVIII. Ejaculatory channels twisted down, moderately long. Ovisacs paired, elongated, convoluted, thin-walled, arranged as loops, extend to XVIII somite. Atrium large, slightly elongated	Crop caeca comprising 7 branched pairs, 7th pair has 4-5 blind processes. Intestine has 4 pairs of short processes. Salivary glands diffuse
H. myanmariana **sp. nov.**	Dorsum yellowish, brownish or whitish, sometimes with unclear longitudinal narrow light brown stripes and rows of light brown dashes. Posterior sucker brownish, with large white spots marginally. Venter yellowish,	Absent	72	I–II uniannulate, III biannulate, IV–XXIV triannulate, XXV biannulate, XXVI–XXVII uniannulate. The head region (I–V) is weakly separated from the body	Two pairs: the first pair partly reduced up to two gray rounded spots and located on the border of II and III somites; the second pair large, separate, cup-like, and located in III somite	XI/XII (male) and in XII a1/a2 (female)	Six pairs of spherical testisacs arranged inter-segmentally on XIII/XVIII. Ejaculatory channels twisted down, moderately long. Ovisacs paired, elongated, convoluted, thin-walled, arranged as loops, extend to XVIII somite. Atrium large, slightly elongated	Crop caeca comprising 7 branched pairs, 7th pair has 4-5 blind processes. Intestine has 4 pairs of short processes. Salivary glands diffuse

Species	Coloration (fixed animals)	Papillation	Total number of annuli	Somites	Eyes	Gonopores	Reproductive system	Digestive system
	sometimes with unclear brownish bands in the anterior third of the animal							
H. schrencki **sp. nov.**	Dorsum orange, with seven rows of ovate yellow spots. Spots in the lateral rows are located on the edge of the last annulus of each somite. Posterior sucker with large yellow spots. Venter dark yellow	Absent	72	I–II uniannulate, III biannulate, IV–XXIV triannulate, XXV biannulate, XXVI–XXVII uniannulate. The head region (I–V) is clearly separated from the body	Two pairs: the first pair partly reduced up to two gray rounded spots and located on the border of II and III somites; the second pair rather small, separate, drop-shaped, and located in III somite	XI/XII (male) and in XII a1/a2 (female)	Six pairs of spherical testisacs arranged inter-segmentally on XIII/XVIII. Ejaculatory channels twisted down, moderately long. Ovisacs paired, elongated, convoluted, thin-walled, arranged as loops, extend to XVIII somite. Atrium large, slightly elongated	Crop caeca comprising 7 branched pairs, 7th pair has 4-5 blind processes. Intestine has 4 pairs of short processes. Salivary glands diffuse
H. tumniniana **sp. nov.**	Dorsum brown or orange, with seven rows of white or yellow spots. Spots of the central row are joined to a broad white or yellow stripe, while spots in other rows may disappear in large specimens. Posterior sucker whitish or yellowish, usually with dense, diffuse brown dots. Venter brownish or light orange with short light bands on the edge of the last annulus of each somite	Seven rows of very low, white tubercles	72	I–II uniannulate, III biannulate, IV–XXIV triannulate, XXV biannulate, XXVI–XXVII uniannulate. The head region (I–V) is clearly separated from the body	Two pairs: the first pair strongly reduced up to two unclear gray spots and located on the border of II and III somites; the second pair of moderate size, separate, rounded, and located in III somite	XI/XII (male) and in XII a1/a2 (female)	Six pairs of spherical testisacs arranged inter-segmentally on XIII/XVIII. Ejaculatory channels twisted down, moderately long. Ovisacs paired, elongated, convoluted, thin-walled, arranged as loops, extend to XVIII somite. Atrium spherical, the atrial cornua directed dorsally	Crop caeca comprising 7 branched pairs, 7th pair has 4-5 blind processes. Intestine has 4 pairs of short processes. Salivary glands diffuse

Supplementary Table 5. The most probable ancestral areas of the primary clades within the Glossiphoniidae inferred from three different statistical modeling approaches. High support values (probability ≥70 %) are highlighted in bold.

Clades	Ancestral areas	Type of biogeographic events	Probability of ancestral areas (%)			
			S-DIVA	DEC	S-DEC	Combined results
Glossiphoniidae	East Asia & North America	Dispersal + Vicariance	**100.0**	**100.0**	**99.6**	**99.9**
Haementeriinae	North America & South America	Vicariance	**100.0**	**75.6**	**84.5**	**86.7**
Glossiphoniinae	East Asia	Dispersal	**100.0**	**100.0**	**95.6**	**98.5**
Glossiphonia	East Asia & Europe	Dispersal	51.8	50.3	**75.0**	58.8
Alboglossiphonia	East Asia & Africa	Vicariance	**100.0**	**77.7**	57.8	**78.5**
Batracobdelloides	East Asia & Africa	Dispersal + Vicariance	**93.0**	**100.0**	**96.7**	**96.6**
Asian *Batracobdelloides* mussel leeches	East Asia & Southeast Asia	Vicariance	**100.0**	**100.0**	**99.0**	**99.7**
Hemiclepsis	East Asia	Intra-area radiation	**78.0**	**83.4**	65.6	**75.8**
Asian *Hemiclepsis* mussel leeches	East Asia & Southeast Asia	Vicariance	**100.0**	**100.0**	**93.2**	**97.7**

Supplementary Table 6. Field observations supporting the general scheme of the life cycle of *Hemiclepsis* mussel-associated leech taxa (Fig. 6)

Stages of life cycle	Stage description	Environment	Date	Locality	Species	Field observations
Stage 1	Mature leech leaves the mantle cavity and fixes cocoon to the dorsal margin of the host shell near umbo	Open environment	28.vi.2018	Gladkaya River, Russian Far East	*H. kasmiana* **comb. rev.**	Record of adult leeches (*N* = 3) laying their cocoons on the dorsal margin of shells of *Middendorffinaia mongolica* and *Buldowskia suifunica*
Stage 2	The parent covers the brood by its flat body. Eggs develop	Open environment	28.vi.2018	Gladkaya River, Russian Far East	*H. kasmiana* **comb. rev.**	Record of adult leeches (*N* = 3) covering their brood on the dorsal margin of shells of *Middendorffinaia mongolica* and *Buldowskia suifunica*
		Open environment	01.vii.2018	Melgunovka River, Khanka Lake Basin, Russian Far East	*H. khankiana* **sp. nov.**	Record of adult leeches (*N* = 2) covering their brood on the dorsal margin of shells of *Nodularia douglasiae*
Stages 3-4	The eggs hatch. The larvae attach to the ventral surface of the parent. The parent with the attached larvae enters the mantle cavity of the host mussel	Open environment => host mussel	23.ii.2018	Myanmar, Salween Basin, Nadi Lake	*H. myanmariana* **sp. nov.**	Record of adult leeches (sample RMBH Hir_0048) with larvae attached to their abdomen inside the mantle cavity of *Lamellidens savadiensis*
Stages 5-6	The larvae leave the parent and probably start to feed on the host mussel. Growth of the juvenile leeches into adults. Proposed feeding on the host mussel (still to be confirmed)	Host mussel	24.x.2016	Russia, Razdolnaya Basin, Soldatskoye Lake	*H. kasmiana* **comb. rev.**	Record of differently sized leeches (from young to adults) [sample RMBH Hir_0015_3] inside the mantle cavity of *Buldowskia suifunica*
		Host mussel	25.x.2016	Russia, Gladkaya River	*H. kasmiana* **comb. rev.**	Record of differently sized leeches (from young to adults) [samples RMBH Hir_0015_1, Hir_0015_2, Hir_0015_4] inside the mantle cavity of *Sinanodonta lauta*, *Buldowskia suifunica*, and *Middendorffinaia mongolica*
		Host mussel	28.vi.2018	Russia, Gladkaya River	*H. kasmiana* **comb. rev.**	Record of differently sized leeches (from young to adults) [samples RMBH Hir_0126, Hir_0127, Hir_0128] inside the mantle cavity of *Sinanodonta lauta*, *Buldowskia suifunica*, and *Middendorffinaia mongolica*
		Host mussel	02.vii.2018	Russia, Khanka Lake basin, Komissarovka River mouth	*H. kasmiana* **comb. rev.**	Record of differently sized leeches (from young to adults) [samples RMBH Hir_0105, Hir_0129_1, Hir_129_2] inside the mantle cavity of *Nodularia douglasiae* and *Cristaria plicata*
		Host mussel	08.vii.2018	South Korea, Geum River	*H. kasmiana* **comb. rev.**	Record of differently sized leeches (from young to adults) [samples RMBH Hir_0106, Hir_0107,

Stages of life cycle	Stage description	Environment	Date	Locality	Species	Field observations
						Hir_0124] inside the mantle cavity of *Nodularia* sp., *Lamprotula gottschei*, and *Aculamprotula koreana*
		Host mussel	10.vii.2018	South Korea, Seomjin River	*H. kasmiana* **comb. rev.**	Record of differently sized leeches (from young to adults) [samples RMBH Hir_0116, Hir_0117, Hir_0118, Hir_119, Hir_125] inside the mantle cavity of *Nodularia douglasiae*, *N. sinuata*, and *Sinanodonta lauta*
		Host mussel	01.viii.2002	Japan, Honshu, Hyakuken Basin, irrigation ditch	*H. kasmiana* **comb. rev.**	Record of differently sized leeches (from young to adults) [samples NCSM-NMI 29630-33, NCSM-NMI 29634-38, NCSM-NMI 29639-42, NCSM-NMI 29643-47] inside the mantle cavity of *Nodularia douglasiae*, *Inversunio yanagawensis*, *Pronodularia japanensis*, and *Obovalis omiensis*
		Host mussel	01.vii.2018	Russia, Khanka Lake basin, Melgunovka River	*H. khankiana* **sp. nov.**	Record of differently sized leeches (from young to adults) [sample RMBH Hir_0101] inside the mantle cavity of *Nodularia douglasiae*
		Host mussel	23.ii.2018	Myanmar, Salween Basin, Nadi Lake	*H. myanmariana* **sp. nov.**	Record of differently sized leeches (from young to adults) [sample RMBH Hir_0048] inside the mantle cavity of *Lamellidens savadiensis*
Stage 7	Maturation of adult leeches with feeding on blood of freshwater fishes as second hosts (confirmed)	Open environment	02.vii.2018	Mouth of the Komissarovka River, Khanka Lake, Russian Far East	*H. kasmiana* **comb. rev.**	Record of one leech (specimen RMBH Hir_0111_5) beneath a stone. Molecular data indicates that this leech specimen used *Perccottus glenii* Dybowski, 1877 (Perciformes: Odontobutidae) as the primary host (Supplementary Table 8).
		Host mussel => open environment => host mussel	01.vii.2018	Russia, Khanka Lake basin, Melgunovka River	*H. kasmiana* **comb. rev.**	Molecular data indicates that the leech specimen RMBH Hir_123_1 used *Silurus asotus* (Siluriformes: Siluridae) as the primary host (Supplementary Table 8). The leech specimen was collected from the mantle cavity of *Nodularia douglasiae*
		Host mussel => open environment => host mussel	23.ii.2018	Myanmar, Salween Basin, Nadi Lake	*H. myanmariana* **sp. nov.**	Molecular data indicates that the leech specimen RMBH Hir_0048_1 used *Labeo rohita* (Cypriniformes: Cyprinidae) as the primary host (Supplementary Table 8). The leech specimen was collected from the mantle cavity of

Stages of life cycle	Stage description	Environment	Date	Locality	Species	Field observations
						Lamellidens savadiensis
		Host mussel => open environment => host mussel	01.vii.2018	Melgunovka River, Khanka Lake basin, Russia	*H. khankiana* **sp. nov.**	Molecular data indicates that the leech specimen RMBH Hir_0123_2 used *Rhodeus uyekii* (Mori, 1935) (Cypriniformes: Cyprinidae) as the primary host (Supplementary Table 8). The leech specimen was collected from the mantle cavity of *Nodularia douglasiae*
		Open environment	08.vii.2018	Geum River, South Korea	*H. kasmiana* **comb. rev.**	Record of 11 leeches (samples RMBH Hir_0103 and Hir_0110) beneath stones
		Open environment	06.vii.2018	Jichon Stream, Han River basin, South Korea	*H. kasmiana* **comb. rev.**	Record of 2 leeches (sample RMBH Hir_0112_3) beneath stones
		Open environment	10.vii.2018	Seomjin River, South Korea	*H. kasmiana* **comb. rev.**	Record of 10 leeches (sample RMBH Hir_0113_2) beneath stones
		Host mussel	23.vi.2012	Russia, Razdolnaya Basin, Komarovka River	*H. kasmiana* **comb. rev.**	Record of large adult leeches [sample RMBH IEPN_256] inside the mantle cavity of *Margaritifera dahurica*
		Host mussel	24.x.2016	Russia, Razdolnaya Basin, Soldatskoye Lake	*H. kasmiana* **comb. rev.**	Record of large adult leeches [sample RMBH Hir_0015_3] inside the mantle cavity of *Buldowskia suifunica*
		Host mussel	25.x.2016	Russia, Gladkaya River	*H. kasmiana* **comb. rev.**	Record of large adult leeches [samples RMBH Hir_0015_1, Hir_0015_2, Hir_0015_4] inside the mantle cavity of *Sinanodonta lauta*, *Buldowskia suifunica*, and *Middendorffinaia mongolica*
		Host mussel	28.vi.2018	Russia, Gladkaya River	*H. kasmiana* **comb. rev.**	Record of large adult leeches [samples RMBH Hir_0126, Hir_0127, Hir_0128] inside the mantle cavity of *Sinanodonta lauta*, *Buldowskia suifunica*, and *Middendorffinaia mongolica*
		Host mussel	02.vii.2018	Russia, Khanka Lake basin, Komissarovka River mouth	*H. kasmiana* **comb. rev.**	Record of large adult leeches [samples RMBH Hir_0105, Hir_0129_1, Hir_129_2] inside the mantle cavity of *Nodularia douglasiae* and *Cristaria plicata*
		Host mussel	08.vii.2018	South Korea, Geum River	*H. kasmiana* **comb. rev.**	Record of large adult leeches [samples RMBH Hir_0106, Hir_0107, Hir_0124] inside the mantle cavity of *Nodularia* sp., *Lamprotula gottschei*, and *Aculamprotula koreana*
		Host mussel	10.vii.2018	South Korea, Seomjin River	*H. kasmiana* **comb. rev.**	Record of large adult leeches [samples RMBH Hir_0116, Hir_0117,

Stages of life cycle	Stage description	Environment	Date	Locality	Species	Field observations
						Hir_0118, Hir_119, Hir_125] inside the mantle cavity of *Nodularia douglasiae*, *N. sinuata*, and *Sinanodonta lauta*
		Host mussel	01.vii.2018	Russia, Khanka Lake basin, Melgunovka River	*H. khankiana* **sp. nov.**	Record of large adult leeches [sample RMBH Hir_0101] inside the mantle cavity of *Nodularia douglasiae*
		Host mussel	23.ii.2018	Myanmar, Salween Basin, Nadi Lake	*H. myanmariana* **sp. nov.**	Record of large adult leeches [sample RMBH Hir_0048] inside the mantle cavity of *Lamellidens savadiensis*

n/a – not available.

Supplementary Table 7. Field observations supporting the general scheme of the life cycle of *Batracobdelloides* mussel-associated leech taxa (Fig. 6)

Stages of life cycle	Stage description	Environment	Date	Locality	Species	Field observations
Stages 1-2	Mature leech places eggs into a tubelike, enclosed cavity in the median section of its abdomen. The eggs develop inside the abdominal cavity of the parent	Host mussel	20.ii.2018	Myanmar, Middle Sittaung Basin, Chain Stream	*B. yaukthwa* **sp. nov.**	Record of one leech with eggs attached to its abdomen [sample RMBH Hir_0062] inside the mantle cavity of *Indochinella pugio viridissima*
Stage 3	The eggs hatch. The larvae attach to the ventral surface of the parent	Host mussel	08.vii.2018	South Korea, Geum River	*B. koreanus* **sp. nov.**	Record of one leech with larvae attached to its abdomen [holotype RMBH Hir_0116] inside the mantle cavity of *Nodularia sinuata*
		Host mussel	10.viii.2018	Uganda, Albert Nile near Pakwach town	*B. tricarinatus*	Record of adult leeches [sample RMBH Hir_0144] with larvae inside its abdominal cavity from the mantle cavity of *Aspatharia* sp.1
		Host mussel	04.iii.2018	Myanmar, Ayeyarwady River, main channel near Mandalay	*B. indochinensis* **sp. nov.**	Record of adult leeches [sample RMBH Hir_0059] with larvae attached to their abdomen inside the mantle cavity of *Trapezidens dolichorhynchus* and *Lamellidens savadiensis*
		Host mussel	18.ii.2018	Myanmar, Bago River	*B. indochinensis* **sp. nov.**	Record of adult leeches [sample RMBH Hir_0064] with larvae attached to their abdomen inside the mantle cavity of *Trapezidens angustior*
Stages 4-5	The larvae leave the parent and probably start to feed on the host mussel. Growth of the juvenile leeches into adults. Proposed feeding on the host mussel (still to be confirmed)	Host mussel	20.ii.2018	Myanmar, Middle Sittaung Basin, Chain Stream	*B. yaukthwa* **sp. nov.**	Record of numerous juvenile leeches [samples RMBH Hir_0060 and Hir_0062] inside the mantle cavity of *Trapezidens angustior* and *Indochinella pugio viridissima*
		Host mussel	14.xi.2018	Myanmar, Hlaingbwe Basin, small stream	*B. hlaingbweensis* **sp. nov.**	Record of differently sized leeches (from juvenile to adults) [samples RMBH Hir_0212 - Hir_0215] inside the mantle cavity of *Pseudodon salwenianus*
		Host mussel	04.iii.2018	Myanmar, Ayeyarwady River, main channel	*B. indochinensis* **sp. nov.**	Record of differently sized leeches (from juveniles to adults) [sample RMBH Hir_0059_1] inside the mantle cavity of *Trapezidens dolichorhynchus* and *Lamellidens savadiensis*
		Host mussel	20.ii.2018	Myanmar, Haungthayaw Basin, small stream	*B. conchophylus* **sp. nov.**	Record of juvenile leeches (sample RMBH Hir_0057) inside the mantle cavity of *Lamellidens generosus*
Stage 6	Maturation of adult leeches	Host mussel => open	01.iii.2018	Myanmar, Middle	*B. indochinensis*	Molecular data indicates that the leech specimen

Stages of life cycle	Stage description	Environment	Date	Locality	Species	Field observations
	with feeding on blood of freshwater fishes as second hosts (confirmed)	environment => host mussel		Sittaung Basin, Mone Ding Dam outlet	**sp. nov.**	RMBH Hir_0056_1 used *Clarias* aff. *batrachus* (Siluriformes: Clariidae) as the primary host (Supplementary Table 8). The leech specimen was collected from the mantle cavity of *Lamellidens savadiensis*
		Host mussel => open environment => host mussel	25.ii.2018	Myanmar, Salween Basin, fish pond near Demoso	*B. indochinensis* **sp. nov.**	Molecular data indicates that the leech specimen RMBH Hir_0066 used *Oreochromis aureus* (Cichliformes: Cichlidae) as the primary host (Supplementary Table 8). The leech specimen was collected from the mantle cavity of *Lamellidens ferrugineus*
		Host mussel => open environment => host mussel	14.xi.2018	Myanmar, Hlaingbwe Basin, small stream	*B. hlaingbweensis* **sp. nov.**	Molecular data indicates that the leech specimen RMBH Hir_0215 used *Hemibagrus nemurus* (Siluriformes: Bagridae) as the primary host (Supplementary Table 8). The leech specimen was collected from the mantle cavity of *Pseudodon salwenianus*
		Host mussel => open environment => host mussel	20.ii.2018	Myanmar, Lower Sittaung Basin, ox-bow lake near Taung Gyi village	*B. conchophylus* **sp. nov.**	Molecular data indicates that the leech specimen RMBH Hir_0065_1 used *Wallago attu* (Siluriformes: Siluridae) as the primary host (Supplementary Table 8). The leech specimen was collected from the mantle cavity of *Lamellidens generosus*
		Host mussel => open environment => host mussel	20.ii.2018	Myanmar, Middle Sittaung Basin, Chain Stream	*B. yaukthwa* **sp. nov.**	Molecular data indicates that the leech specimen RMBH Hir_0060_1 used *Macrognathus dorsiocellatus* (Synbranchiformes: Mastacembelidae) as the primary host (Supplementary Table 8). The leech specimen was collected from the mantle cavity of *Trapezidens angustior*
		Host mussel => open environment => host mussel	11.vii.2018	South Korea, Mangyeong River, irrigation channel	*B. koreanus* **sp. nov.**	Molecular data indicates that the leech specimen RMBH Hir_0104 used *Channa argus* (Anabantiformes: Channidae) as the primary host (Supplementary Table 8). The leech specimen was collected from the mantle cavity of *Nodularia sinuata*
		Host mussel => open environment => host	10.viii.2018	Uganda, Albert Nile near Pakwach	*B. tricarinatus*	Molecular data indicates that the leech specimen RMBH Hir_0139 used *Synodontis frontosus*

Stages of life cycle	Stage description	Environment	Date	Locality	Species	Field observations
		mussel		town		(Siluriformes: Mochokidae) as the primary host (Supplementary Table 8). The leech specimen was collected from the mantle cavity of *Aspatharia* sp.
		Host mussel	05.viii.2018	Uganda, Albert Nile Basin, Lake George	*B. tricarinatus*	Record of large adult leeches [samples RMBH Hir_0138] inside the mantle cavity of *Coelatura aegyptiaca*
		Host mussel	10.viii.2018	Uganda, Albert Nile near Pakwach town	*B. tricarinatus*	Record of large adult leeches [samples RMBH Hir_0139 – Hir_143] inside the mantle cavity of *Coelatura aegyptiaca*, *Aspatharia* sp.1, *A.* sp.2, Chambardia sp., and *Mutela* sp.
		Host mussel	20.ii.2018	Myanmar, Middle Sittaung Basin, Chain Stream	*B. yaukthwa* **sp. nov.**	Record of large adult leeches [samples RMBH Hir_0060 and Hir_0062] inside the mantle cavity of *Trapezidens angustior* and *Indochinella pugio viridissima*
		Host mussel	11.vii.2018	South Korea, Mangyeong River, irrigation channel	*B. koreanus* **sp. nov.**	Record of one large leech [holotype RMBH Hir_0104] from the mantle cavity of *Nodularia sinuata*
		Host mussel	04.iii.2018	Myanmar, Ayeyarwady River, main channel near Mandalay	*B. indochinensis* **sp. nov.**	Record of large adult leeches [sample RMBH Hir_0059] inside the mantle cavity of *Trapezidens dolichorhynchus* and *Lamellidens savadiensis*
		Host mussel	14.xi.2018	Myanmar, Hlaingbwe Basin, small stream	*B. hlaingbweensis* **sp. nov.**	Record of large adult leeches [samples RMBH Hir_0212 - Hir_0215] inside the mantle cavity of *Pseudodon salwenianus*
		Host mussel	04.iii.2018	Myanmar, Ayeyarwady River, main channel	*B. indochinensis* **sp. nov.**	Record of large adult leeches [sample RMBH Hir_0059_1] inside the mantle cavity of *Trapezidens dolichorhynchus* and *Lamellidens savadiensis*

n/a – not available.

Supplementary Table 8. Molecular identification of the primary hosts based on molecular analyses of the crop content of mature mussel-associated leech species and two free-living leech taxa new to science

Leech species	Country and leech sample ID	GenBank acc. No. for the *COI* sequences of the crop content	Taxonomic identification of the primary hosts (freshwater fish species) based on the *COI* sequences of the crop content
**Mussel-associated leeches**			
B. conchophylus **sp. nov.**	Myanmar [RMBH Hir_065_1]	MN605506	*Wallago attu* (Bloch & Schneider, 1801) (Siluriformes: Siluridae)
B. hlaingbweensis **sp. nov.**	Myanmar [RMBH Hir_0215]	MN605516	*Hemibagrus nemurus* (Valenciennes, 1840) (Siluriformes: Bagridae)
B. indochinensis **sp. nov.**	Myanmar [RMBH Hir_0056_1]	MN605504	*Clarias* aff. *batrachus* (Linnaeus, 1758) (Siluriformes: Clariidae)
	Myanmar [RMBH Hir_0066]	MN605507	*Oreochromis aureus* (Steindachner, 1864) (Cichliformes: Cichlidae)
B. yaukthwa **sp. nov.**	Myanmar [RMBH Hir_0060_1]	MN605505	*Macrognathus dorsiocellatus* Britz, 2010 (Synbranchiformes: Mastacembelidae)
B. koreanus **sp. nov.**	South Korea [RMBH Hir_0104]	MN605511	*Channa argus* (Cantor, 1842) (Anabantiformes: Channidae)
B. tricarinatus	Uganda [RMBH Hir_0139]	MN605515	*Synodontis frontosus* Vaillant, 1895 (Siluriformes: Mochokidae)
H. kasmiana **comb. rev.**	South Korea [RMBH Hir_0111_5]	MN605512	*Perccottus glenii* Dybowski, 1877 (Perciformes: Odontobutidae)
	Russia [RMBH Hir_0123_1]	MN605513	*Silurus asotus* Linnaeus, 1758 (Siluriformes: Siluridae)
H. khankiana **sp. nov.**	Russia [RMBH Hir_0123_2]	MN605514	*Rhodeus uyekii* (Mori, 1935) (Cypriniformes: Cyprinidae)
H. myanmariana **sp. nov.**	Myanmar [RMBH Hir_0048_1]	MN605503	*Labeo rohita* (Hamilton, 1822) (Cypriniformes: Cyprinidae)
**Free-living leeches**			
H. schrencki **sp. nov.**	Russia [RMBH Hir_0091_1]	MN605509	*Barbatula toni* (Dybowski, 1869) (Cypriniformes: Nemacheilidae)
	Russia [RMBH Hir_0088_1]	MN605508	*Phoxinus* sp. "Amur" (Cypriniformes: Cyprinidae)
H. tumniniana **sp. nov.**	Russia [RMBH Hir_0093]	MN605510	*Pungitius pungitius* (Linnaeus, 1758) (Gasterosteiformes: Gasterosteidae)

Supplementary Table 9. Infestation of freshwater mussels by leeches in East Asia, Southeast Asia, and East Africa

Leech species [leech sample ID]	Host mussel species (family: tribe) [mussel sample ID]	Collecting location, habitat	Date of collecting	Total number of mussels	Total number of adult and juvenile leeches (without larvae)	Leech Infestation Prevalence index (*LIP*, %)	Mean intensity of leech infestation (*ILI* ± s.e.m., l.p.m.)
**East Asia**							
H. kasmiana **comb. rev.** [RMBH Hir_0015_5]	*Margaritifera dahurica* (Margaritiferidae)	Russia, Razdolnaya Basin, Komarovka River, 43.6392°N, 132.1614°E	23.vi.2012	16	8	18.8	0.50±0.33
H. kasmiana **comb. rev.** [RMBH Hir_0015_3]	*Buldowskia suifunica* (Unionidae: Cristariini) [RMBH biv0227]	Russia, Razdolnaya Basin, Soldatskoye Lake, 3.7747°N, 131.9406°E	24.x.2016	11	25	100	2.27*
H. kasmiana **comb. rev.** [RMBH Hir_0015_1, Hir_0015_2]	*Sinanodonta lauta* and *Buldowskia suifunica* (Unionidae: Cristariini) [RMBH biv0225]	Russia, Gladkaya River, 42.7065°N, 130.9084°E	25.x.2016	27	190	100	7.04±2.12
H. kasmiana **comb. rev.** [RMBH Hir_0015_4]	*Middendorffinaia mongolica* (Unionidae: Unionini) [RMBH biv0229]	Ditto	Ditto	15	57	73.3	3.80*
H. kasmiana **comb. rev.** [RMBH Hir_0127]	*Buldowskia suifunica* (Unionidae: Cristariini)	Ditto	28.vi.2018	13	69	92.3	5.31±1.01
H. kasmiana **comb. rev.** [RMBH Hir_0128]	*Middendorffinaia mongolica* (Unionidae: Unionini)	Ditto	Ditto	12	53	66.7	4.42±3.25
H. kasmiana **comb. rev.** [RMBH Hir_0126]	*Sinanodonta lauta* (Unionidae: Cristariini)	Ditto	Ditto	6	11	83.3	1.83±0.60
H. kasmiana **comb. rev.** [RMBH Hir_0096]	*Nodularia douglasiae* (Unionidae: Unionini) [RMBH biv0134_1]	Russia, Amur Basin, Ulbinka River, 49.9535°N, 136.6319°E	21.vii.2014	13	2	15.4	0.15±0.10
H. kasmiana **comb. rev.** [RMBH Hir_0105]	*Nodularia douglasiae* (Unionidae: Unionini) [RMBH biv0503]	Russia, Khanka Lake basin, Komissarovka River mouth, 44.8255°N, 132.0456°E	02.vii.2018	49	30	40.8	0.61±0.13
H. kasmiana **comb. rev.** [RMBH Hir_0129_1,	*Cristaria plicata* (Unionidae: Cristariini)	Ditto	Ditto	3	3	66.7	0.67±0.33

Leech species [leech sample ID]	Host mussel species (family: tribe) [mussel sample ID]	Collecting location, habitat	Date of collecting	Total number of mussels	Total number of adult and juvenile leeches (without larvae)	Leech Infestation Prevalence index (*LIP*, %)	Mean intensity of leech infestation (*ILI* ± s.e.m., l.p.m.)
Hir_129_2]							
H. kasmiana **comb. rev.** [RMBH Hir_0102, Hir_0122]	*Buldowskia shadini* and *Sinanodonta schrenckii* (Unionidae: Cristariini) [RMBH biv0497 and biv0496]	Russia, Khanka Lake basin, Melgunovka River, 44.5939°N, 132.1818°E	01.vii.2018	8	3	25.0	0.38±0.26
H. kasmiana **comb. rev.** [RMBH Hir_0123_1] and *H. khankiana* **sp. nov.** [RMBH Hir_0123_2]	*Nodularia douglasiae* (Unionidae: Unionini) [RMBH biv0498]	Ditto	Ditto	26	5	15.4	0.19±0.10
H. kasmiana **comb. rev.** [RMBH Hir_0106]	*Aculamprotula koreana* (Unionidae: Aculamprotulini) [RMBH biv0514]	South Korea, Geum River, 35.9891°N, 127.5836°E	08.vii.2018	3	3	33.3	1.00±1.00
H. kasmiana **comb. rev.** [RMBH Hir_0107]	*Lamprotula gottschei* (Unionidae: Lamprotulini) [RMBH biv0511]	Ditto	Ditto	14	3	21.4	0.21±0.11
H. kasmiana **comb. rev.** [RMBH Hir_0124]	*Nodularia* sp. (Unionidae: Unionini) [RMBH biv0513]	Ditto	Ditto	7	24	57.1	3.43±2.32
H. kasmiana **comb. rev.** [RMBH Hir_0117]	*Nodularia douglasiae* (Unionidae: Unionini) [RMBH biv0518]	South Korea, Seomjin River, 35.7010°N, 127.2845°E	10.vii.2018	4	7	100	1.75±0.48
H. kasmiana **comb. rev.** [RMBH Hir_0116] and *B. koreanus* **sp. nov.** [RMBH Hir_0116_1-H]	*Nodularia sinuata* (Unionidae: Unionini) [RMBH biv0517]	Ditto	Ditto	23	52	87.0	2.26±0.42
H. kasmiana **comb. rev.** [RMBH Hir_0118]	*Sinanodonta lauta* (Unionidae: Cristariini) [RMBH biv0519]	Ditto	Ditto	2	19	100	9.50±2.50
H. kasmiana **comb. rev.** [RMBH Hir_0119]	*Nodularia douglasiae* (Unionidae: Unionini) [RMBH biv0522]	South Korea, Seomjin River, 35.4217°N, 127.2228°E	10.vii.2018	10	19	90.0	1.90±0.41
H. kasmiana **comb. rev.** [RMBH Hir_0125]	*Nodularia sinuata* (Unionidae: Unionini)	Ditto	Ditto	33	26	48.5	0.79±0.18
H. kasmiana **comb. rev.** [RMBH Hir_0120]	*Sinanodonta lauta* (Unionidae: Cristariini) [RMBH biv0523]	South Korea, Mangyeong River, irrigation	11.vii.2018	8	1	12.5	0.13±0.13

Leech species [leech sample ID]	Host mussel species (family: tribe) [mussel sample ID]	Collecting location, habitat	Date of collecting	Total number of mussels	Total number of adult and juvenile leeches (without larvae)	Leech Infestation Prevalence index (*LIP*, %)	Mean intensity of leech infestation (*ILI* ± s.e.m., l.p.m.)
		channel, 35.9165°N, 127.7135°E					
H. kasmiana **comb. rev.** [RMBH Hir_0121] and *B. koreanus* **sp. nov.** [RMBH Hir_0104]	*Nodularia sinuata* (Unionidae: Unionini) [RMBH biv0524]	Ditto	Ditto	10	3	30.0	0.30±0.15
H. kasmiana **comb. rev.** [sample was lost]	*Nodularia* sp. (Unionidae: Unionini) [RMBH biv0509]	South Korea, Han Basin, Bukhang River, 38.0988°N, 127.6957°E	06.vii.2018	21	1	4.8	0.05±0.05
H. kasmiana **comb. rev.** [NCSM-NMI 29630-33]	*Nodularia douglasiae* (Unionidae: Unionini) [NCSM-Mollusks 27180]	Japan, Honshu Island, Hyakuken Basin, irrigation ditch, 34.6892°N, 133.9644°E	01.viii.2002	5	31	80.0	6.20±1.91
H. kasmiana **comb. rev.** [NCSM-NMI 29634-38]	*Inversunio yanagawensis* (Unionidae: Unionini) [NCSM-Mollusks 27181]	Ditto	01.viii.2002	5	54	100	10.80±3.71
H. kasmiana **comb. rev.** [NCSM-NMI 29639-42]	*Pronodularia japanensis* (Unionidae: Lamprotulini) [NCSM-Mollusks 27183]	Ditto	01.viii.2002	8	88	50.0	11.00±4.88
H. kasmiana **comb. rev.** [NCSM-NMI 29643-47]	*Obovalis omiensis* (Unionidae: Gonideini) [NCSM-Mollusks 27184]	Ditto	01.viii.2002	5	67	100	13.40±5.00
H. khankiana **sp. nov.** [RMBH Hir_0101]	*Nodularia douglasiae* (Unionidae: Unionini)	Russia, Khanka Lake basin, Melgunovka River, 44.5804°N, 132.0803°E	01.vii.2018	59	154	67.8	2.61±0.43
**Mean ± s.e.m. (*N* = 28)**						**60.00±6.24**	**3.30±0.72**
**Southeast Asia**							
H. myanmariana **sp. nov.** [sample was lost]	*Lamellidens savadiensis* (Unionidae: Lamellidentini) [RMBH biv0257]	Myanmar, Ayeyarwady Basin, Nant Phar Lake, 24.2973°N, 97.2610°E	29.xi.2016	12	1	8.3	0.08±0.08
H. myanmariana	*Lamellidens*	Myanmar,	11.xii.2018	26	2	7.7	0.08±0.05

Leech species [leech sample ID]	Host mussel species (family: tribe) [mussel sample ID]	Collecting location, habitat	Date of collecting	Total number of mussels	Total number of adult and juvenile leeches (without larvae)	Leech Infestation Prevalence index (*LIP*, %)	Mean intensity of leech infestation (*ILI* ± s.e.m., l.p.m.)
**sp. nov.** [RMBH Hir_0210, Hir_0211]	*savadiensis* (Unionidae: Lamellidentini) [RMBH biv0672]	Ayeyarwady Basin, Nga Wun River near Pyay town, 18.8624°N, 95.2822°E					
H. myanmariana **sp. nov.** [RMBH Hir_0051]	*Lamellidens savadiensis* (Unionidae: Lamellidentini) [RMBH biv0427A]	Myanmar, Ayeyarwady Basin, ox-bow lake near Ta Naung Taig village, 21.4064°N, 95.3399°E	03.iii.2018	22	1	4.5	0.05±0.05
H. myanmariana **sp. nov.** [RMBH Hir_0052]	*Lamellidens generosus* (Unionidae: Lamellidentini) [RMBH biv0372]	Myanmar, Bilin Basin, Shwe Laung Lake near Pyintha village, 17.4395°N, 97.2457°E	13.ii.2018	12	1	8.3	0.08±0.08
H. myanmariana **sp. nov.** [RMBH Hir_0048_1]	*Lamellidens savadiensis* (Unionidae: Lamellidentini) [RMBH biv0399]	Myanmar, Salween Basin, Nadi Lake, 20.6858°N, 96.9316°E	23.ii.2018	23	117	83.3	4.88±1.57
H. myanmariana **sp. nov.** [RMBH Hir_0054]	*Lamellidens generosus* (Unionidae: Lamellidentini) [RMBH biv0392]	Myanmar, Lower Sittaung Basin, Pangaing Stream, 17.7080°N, 96.7155°E	20.ii.2018	12	1	8.3	0.08±0.08
H. myanmariana **sp. nov.** [RMBH Hir_0056_2] and *B. indochinensis* **sp. nov.** [RMBH Hir_0056_1]	*Lamellidens savadiensis* (Unionidae: Lamellidentini) [RMBH biv0415]	Myanmar, Middle Sittaung Basin, Mone Ding Dam outlet, 20.8099°N, 95.7242°E	01.iii.2018	8	3	25.0	0.38±0.26
H. myanmariana **sp. nov.** [RMBH Hir_0099]	*Indonaia andersoniana* (Unionidae: Indochinellini) [RMBH biv0414]	Ditto	Ditto	6	1	16.7	0.17±0.17
H. myanmariana **sp. nov.** [RMBH Hir_0061]	*Lamellidens savadiensis* (Unionidae: Lamellidentini) [RMBH biv0413]	Myanmar, Middle Sittaung Basin, Sin Thay Dam outlet, 20.1540°N, 96.1149°E	01.iii.2018	10	1	10.0	0.10±0.10

Leech species [leech sample ID]	Host mussel species (family: tribe) [mussel sample ID]	Collecting location, habitat	Date of collecting	Total number of mussels	Total number of adult and juvenile leeches (without larvae)	Leech Infestation Prevalence index (*LIP*, %)	Mean intensity of leech infestation (*ILI* ± s.e.m., l.p.m.)
H. myanmariana **sp. nov.** [sample was lost]	*Lamellidens savadiensis* (Unionidae: Lamellidentini) [RMBH biv0676]	Myanmar, Ayeyarwady Basin, small stream, 25.2758°N, 97.2722°E	23.iii.2018	5	1	20.0	0.20±0.20
B. conchophylus **sp. nov.** [RMBH Hir_0055]	*Radiatula mouhoti* (Unionidae: Indochinellini) [RMBH biv0409]	Myanmar, Middle Sittaung River near Kanna village, 19.4857°N, 96.2750°E	28.ii.2018	18	2	11.1	0.11±0.08
B. conchophylus **sp. nov.** [RMBH Hir_0065_1]	*Lamellidens generosus* (Unionidae: Lamellidentini) [RMBH biv0393]	Myanmar, Lower Sittaung Basin, ox-bow lake near Taung Gyi village, 17.8807°N, 96.8313°E	20.ii.2018	14	4	14.3	0.29±0.22
B. conchophylus **sp. nov.** [RMBH Hir_0057]	*Lamellidens generosus* (Unionidae: Lamellidentini) [RMBH biv0364]	Myanmar, Haungthayaw Basin, small stream, 16.5365°N, 98.2202°E	09.ii.2018	7	4	57.1	0.57±0.20
B. hlaingbweensis **sp. nov.** [RMBH Hir_0209]	*Pseudodon* sp.1 (Unionidae: Pseudodontini) [RMBH biv0638]	Myanmar, Hlaingbwe Basin, small stream, 17.0292°N, 97.8099°E	17.xi.2018	12	10	58.3	0.83±0.24
B. hlaingbweensis **sp. nov.** [RMBH Hir_0207]	*Pseudodon salwenianus* (Unionidae: Pseudodontini) [RMBH biv0639]	Myanmar, Hlaingbwe Basin, small stream, 17.0292°N, 97.8099°E	17.xi.2018	10	10	70.0	1.00±0.30
B. hlaingbweensis **sp. nov.** [RMBH Hir_0212 - Hir_0215]	*Pseudodon salwenianus* (Unionidae: Pseudodontini) [RMBH biv0674]	Myanmar, Hlaingbwe Basin, small stream, 17.0483°N, 97.8194°E	14.xi.2018	8	15	50.0	1.88±0.91
B. indochinensis **sp. nov.** [RMBH Hir_0053_1]	*Lamellidens generosus* (Unionidae: Lamellidentini) [RMBH biv0376]	Myanmar, Bago - Sittaung channel, 17.5818°N, 96.7733°E	16.ii.2018	12	7	41.7	0.58±0.23
B. indochinensis **sp. nov.** [RMBH Hir_0066]	*Lamellidens ferrugineus* (Unionidae: Lamellidentini) [RMBH biv0404]	Myanmar, Salween Basin, fish pond near Demoso,	25.ii.2018	22	4	18.2	0.18±0.08

Leech species [leech sample ID]	Host mussel species (family: tribe) [mussel sample ID]	Collecting location, habitat	Date of collecting	Total number of mussels	Total number of adult and juvenile leeches (without larvae)	Leech Infestation Prevalence index (*LIP*, %)	Mean intensity of leech infestation (*ILI* ± s.e.m., l.p.m.)
		19.7289°N, 97.1167°E					
B. indochinensis **sp. nov.** [RMBH Hir_0059_1] and *H. myanmariana* **sp. nov.** [RMBH Hir_0059]	*Trapezidens dolichorhynchus* and *Lamellidens savadiensis* (Unionidae: Lamellidentini) [RMBH biv0442]	Myanmar, Ayeyarwady River, main channel, 21.9909°N, 96.0610°E	04.iii.2018	23	33	34.8	1.43±0.70
B. indochinensis **sp. nov.** [RMBH Hir_0206]	*Lamellidens savadiensis* (Unionidae: Lamellidentini) [RMBH biv0603]	Myanmar, Ayeyarwady Basin, Indaw Lake, 24.2665°N, 96.1228°E	13.xi.2018	27	1	3.7	0.04±0.04
B. indochinensis **sp. nov.** [RMBH Hir_0050]	*Lamellidens savadiensis* (Unionidae: Lamellidentini) [RMBH biv0439]	Myanmar, Ayeyarwady Basin, Sih Khong Stream, 22.0632°N, 96.0810°E	04.iii.2018	15	2	13.3	0.13±0.09
B. indochinensis **sp. nov.** [RMBH Hir_0064_1]	*Trapezidens angustior* (Unionidae: Lamellidentini) [RMBH biv0382]	Myanmar, Bago River, 17.5334°N, 96.3315°E	18.ii.2018	26	2	7.7	0.08±0.05
B. indochinensis **sp. nov.** [RMBH Hir_239]	*Lamellidens generosus* and *Trapezidens angustior* (Unionidae: Lamellidentini) [RMBH biv0250]	Myanmar, Middle Sittaung Basin, Myit Kyi Pauk Stream, 18.9613°N, 96.4455°E	26.xi.2016	20	6	20.0	0.30±0.16
B. yaukthwa **sp. nov.** [RMBH Hir_0060_1]	*Trapezidens angustior* (Unionidae: Lamellidentini) [RMBH biv0394]	Myanmar, Middle Sittaung Basin, Chain Stream, 17.9769°N, 96.7650°E	20.ii.2018	9	19	66.7	2.11±0.87
B. yaukthwa **sp. nov.** [RMBH Hir_0062]	*Indochinella pugio viridissima* (Unionidae: Indochinellini) [RMBH biv0395]	Ditto	Ditto	18	5	22.2	0.28±0.14
B. yaukthwa **sp. nov.** [RMBH Hir_0062]	*Trapezidens angustior* (Unionidae: Lamellidentini) [RMBH biv0394]	Ditto	21.ii.2018	10	20	40.0	2.00±1.06
B. yaukthwa **sp. nov.** [RMBH Hir_0062]	*Indochinella pugio viridissima* (Unionidae: Indochinellini) [RMBH biv0395]	Ditto	Ditto	60	21	28.3	0.35±0.08

**Leech species [leech sample ID]**	**Host mussel species (family: tribe) [mussel sample ID]**	**Collecting location, habitat**	**Date of collecting**	**Total number of mussels**	**Total number of adult and juvenile leeches (without larvae)**	**Leech Infestation Prevalence index (*LIP*, %)**	**Mean intensity of leech infestation (*ILI* ± s.e.m., l.p.m.)**
**Mean ± s.e.m. (*N* = 27)**						**27.76±4.38**	**0.68±0.20**
**East Africa**							
B. tricarinatus [RMBH Hir_0138]	*Coelatura aegyptiaca* (Unionidae: Coelaturini) [RMBH biv0538, biv0539]	Uganda, Albert Nile Basin, Lake George, 0.0476°S, 30.1642°E	05.viii.2018	138	15	10.9	0.11±0.03
B. tricarinatus [RMBH Hir_0139, Hir_0141, Hir_0143, Hir_0144]	*Aspatharia* sp.1 and *A.* sp.2 (Iridinidae) [RMBH biv0543, biv0547, biv0549]	Uganda, Albert Nile near Pakwach town, 2.4579°N, 31.4964°E	10.viii.2018	165	7	2.4	0.04±0.02
B. tricarinatus [RMBH Hir_0140]	*Mutela* sp. (Iridinidae) [RMBH biv0541]	Ditto	Ditto	55	2	3.6	0.04±0.03
B. tricarinatus [RMBH Hir_0142]	*Chambardia* sp. (Iridinidae) [RMBH biv0546]	Ditto	Ditto	43	8	16.3	0.19±0.07
**Mean ± s.e.m. (*N* = 4)**						**8.30±3.26**	**0.10±0.04**

*The s.e.m. is not available because a general sample of leeches from all sampled mussels was collected.

Supplementary Table 10. Primer sequences and PCR conditions

Gene fragment	Primers`s name (direction)	Primer sequence (5'-3')	PCR conditions	Reference
COI (leeches)	C1-N-2329 (reverse)	actgtaaatatatgatgagctca	95°C (5 min), followed by 33 cycles at 95°C (50 sec), 48°C (50 sec), 72°C (60 sec) and a final extension at 72°C (5 min)	Ref. 1
	LoboF1 (forward)	kbtchacaaaycayaargayathgg	95°C (5 min), followed by 31 cycles at 95°C (50 sec), 48°C (50 sec), 72°C (60 sec) and a final extension at 72°C (5 min)	Ref. 2
	LoboR1 (reverse)	taaacytcwggrtgwccraaraayca		
18S rRNA (leeches)	1F (forward)	tacctggttgatcctgccagtag	95°C (5 min), followed by 31 cycles at 95°C (50 sec), 65°C (50 sec), 72°C (60 sec) and a final extension at 72°C (5 min)	Ref. 3
	4R (reverse)	gaattaccgcggctgctgg		
	3F (forward)	gttcgattccggagaggga	95°C (5 min), followed by 26 cycles at 95°C (50 sec), 59°C (50 sec), 72°C (60 sec) and a final extension at 72°C (5 min)	Ref. 4
	bi (reverse)	gagtctcgttcgttatcgga		
	a.2.0 (forward)	atggttgcaaagctgaaac	95°C (5 min), followed by 31 cycles at 95°C (50 sec), 65°C (50 sec), 72°C (60 sec) and a final extension at 72°C (5 min)	Ref. 3
	9R (reverse)	gatccttccgcaggttcacctac		
COI (fish hosts)	FishF1 (forward)	tcaaccaaccacaaagacattggcac	95°C (5 min), followed by 25 cycles at 95°C (50 sec), 61°C (50 sec), 72°C (60 sec) and a final extension at 72°C (5 min)	Ref. 5
	FishR1 (reverse)	tagacttctgggtggccaaagaatca		

Supplementary Table 11. Models of sequence evolution for each partition used in the phylogenetic reconstructions

Partitions	Models			
	MrBayes v3.2.6*	IQ-TREE v1.6.11 [*COI+18S rRNA*]**	IQ-TREE v1.6.11 [*COI*]**	BEAST v1.10.4***
COI				
1st codon of *COI*	GTR+G+I	GTR+F+G4	TIM3e+I+G4	HKY+G+I
2nd codon of *COI*	GTR+G	TPM2u+F+ASC+G4	TPM3u+F+I+G4	HKY+G
3rd codon of *COI*	GTR+G+I	SYM+I+G4	GTR+F+ASC+G4	HKY+G+I
18S rRNA	GTR+G+I	SYM+I+G4	n/a	HKY+G+I

*Based on AICc of MEGA7[6]. **Based on BIC of Model Finder[7] implemented in the IQ-TREE web server[8]. ***Simplified version of the MrBayes models[9]. n/a – not available.

Supplementary Note 1

Checklist of the genera *Batracobdelloides* Oosthuizen, 1986 and *Hemiclepsis* Vejdovsky, 1884

Suborder Glossiphoniiformes Tessler & de Carle, 2018

Family Glossiphoniidae Vaillant, 1890

Subfamily Glossiphoniinae Vaillant, 1890

Genus *Batracobdelloides* Oosthuizen, 1986

Type species: *Helobdella tricarinata* Blanchard, 1897 (by original designation)

Batracobdelloides amnicolus (Moore, 1958) **stat. rev.**

=*Batracobdella amnicola* Moore (1958): p. 313[10].

=*Batracobdelloides amnicola* (Moore, 1958). – Oosthuizen (1989): p. 154[11], as a synonym of *B. tricarinatus*.

Type locality. SOUTH AFRICA: Engamani River, Hluhluwe, Zululand.

Type. Holotype, in the United States National Museum, Washington DC, USA[11].

Material sequenced. One specimen from South Africa (Supplementary Table 1).

Life style and hosts. Free-living species, which uses freshwater fishes *Clarias gariepinus* (Clariidae), *Labeobarbus kimberleyensis*, *Carassius auratus* (Cyprinidae), *Oreochromis mossambicus* (Cichlidae)[11] and unspecified amphibians[12] as hosts.

Distribution. Republic of South Africa and Namibia[11,12].

Comments. Here, we resurrect this nominal taxon based on our two-locus phylogenetic reconstruction showing the species-level differences between specimens from South Africa and the Nile Basin (Fig. 2). The mean *COI* p-distance between this species and *B. tricarinatus* is 5.8%.

Batracobdelloides conchophylus Bolotov, Klass, Bespalaya, Konopleva, Kondakov & Vikhrev **sp. nov.**

Figs. 3A, 4A, 5A, Table 2, Supplementary Table 4, Supplementary Figs. 7D, 10A

Type locality. MYANMAR: Lower Sittaung Basin, ox-bow lake near Taung Gyi village, 17.8807°N, 96.8313°E, from the mantle cavity of *Lamellidens generosus*.

Type. Holotype RMBH Hir_0065_1-H, in the Russian Museum of Biodiversity Hotspots, Federal Center for Integrated Arctic Research, Russian Academy of Sciences, Arkhangelsk, Russia.

Material examined. MYANMAR: Lower Sittaung Basin, ox-bow lake near Taung Gyi village, 17.8807°N, 96.8313°E, from the mantle cavity of *Lamellidens generosus*, 20.ii.2018, **4 specimens** [RMBH Hir_0065_1 including the holotype and one paratype], Bolotov, Vikhrev, and Nyein Chan leg.; Sittaung River near Kanna village, 19.4857°N, 96.2750°E, from the mantle cavity of *Radiatula mouhoti*, 28.ii.2018, **2 specimens** [RMBH Hir_0055, including one paratype], Bolotov, Vikhrev, and Nyein Chan leg.; Haungthayaw Basin, small stream, 16.5365°N, 98.2202°E, from the mantle cavity of *Lamellidens generosus*, 09.ii.2018, **4 specimens** [RMBH Hir_0057], Nyein Chan and local villagers leg.

Material sequenced. Two specimens from the Sittaung Basin, Myanmar (Supplementary Table 1).

Life style and hosts. This mussel-associated leech species seems to be a host mussel specialist being a possible obligate inhabitant of the mantle cavity of freshwater mussels as a secondary host and shelter in the earlier developmental stages (Supplementary Table 9). It was collected from three freshwater mussel species belonging to two tribes and one subfamily: *Lamellidens generosus, L. savadiensis* (Unionidae: Lamellidentini), and *Radiatula mouhoti* (Unionidae: Indochinellini). Adult leeches use freshwater fishes as the primary host: *Wallago attu* (Siluriformes: Siluridae) (Supplementary Table 8).

Distribution. Sittaung and Haungthayaw river basins, Myanmar.

Comments. Genetically, this species is most closely related to *Batracobdelloides indochinensis* **sp. nov.** from Myanmar (mean *COI* p-distance = 4.1%).

Batracobdelloides hlaingbweensis Bolotov, Klass, Bespalaya, Konopleva, Kondakov & Vikhrev **sp. nov.**

Figs. 3B, 4B, 5B, Table 2, Supplementary Table 4, Supplementary Figs. 7A, 10B

Type locality. MYANMAR: Hlaingbwe Basin, small stream, 17.0292°N, 97.8099°E, from the mantle cavity of *Pseudodon salwenianus*.

Type. Holotype RMBH Hir_0207-H, in the Russian Museum of Biodiversity Hotspots, Federal Center for Integrated Arctic Research, Russian Academy of Sciences, Arkhangelsk, Russia.

Material examined. MYANMAR: Hlaingbwe Basin, small stream, 17.0292°N, 97.8099°E, from the mantle cavity of *Pseudodon salwenianus*, 17.xi.2018, **10 specimens** [RMBH

Hir_0207, including the holotype and one paratype], from the mantle cavity of *Pseudodon* sp.1, **10 specimens** [RMBH Hir_0209 including one paratype], Than Win leg.; Hlaingbwe Basin, small stream, 17.0483°N, 97.8194°E, from the mantle cavity of *Pseudodon salwenianus*, 14.xi.2018, **15 specimens** [RMBH Hir_0212 - Hir_0215 including two paratypes RMBH Hir_0214 and Hir_0215], Than Win leg.

Material sequenced. Four specimens from the Hlaingbwe Basin, Myanmar (Supplementary Table 1).

Life style and hosts. This mussel-associated leech species seems to be a host mussel specialist being a possible obligate inhabitant of the mantle cavity of freshwater mussels as a secondary host and shelter in the earlier developmental stages (Supplementary Table 9). It was collected from two freshwater mussel species belonging to one genus: *Pseudodon salwenianus* and *Pseudodon* sp.1 (Unionidae: Pseudodontini). Adult leeches use freshwater fishes as the primary host: *Hemibagrus nemurus* (Siluriformes: Bagridae) (Supplementary Table 8).

Distribution. Hlaingbwe River basin, Myanmar.

Comments. Genetically, this species is most closely related to *Batracobdelloides yaukthwa* **sp. nov.** from Myanmar (mean *COI* p-distance = 3.8%).

Batracobdelloides indochinensis Bolotov, Klass, Bespalaya, Konopleva, Kondakov & Vikhrev **sp. nov.**

Figs. 3C, 4C, 5C, Table 2, Supplementary Table 4, Supplementary Figs. 7B, 10C

Type locality. MYANMAR: Salween Basin, fish pond near Demoso, 19.7289°N, 97.1167°E, from the mantle cavity of *Lamellidens ferrugineus*.

Type. Holotype RMBH Hir_0066-H, in the Russian Museum of Biodiversity Hotspots, Federal Center for Integrated Arctic Research, Russian Academy of Sciences, Arkhangelsk, Russia.

Material examined. MYANMAR: Salween Basin, fish pond near Demoso, 19.7289°N, 97.1167°E, from the mantle cavity of *Lamellidens ferrugineus*, 25.ii.2018, **4 specimens** [RMBH Hir_0066 including the holotype and one paratype], Bolotov, Vikhrev, and Nyein Chan leg.; Bago-Sittaung Channel, 17.5818°N, 96.7733°E, from the mantle cavity of *Lamellidens generosus*, 16.ii.2018, **7 specimens** [RMBH Hir_0053 including one paratype], Bolotov, Vikhrev, and Nyein Chan leg.; Middle Sittaung Basin, Mone Ding Dam outlet, 20.8099°N, 95.7242°E, from the mantle cavity of *Lamellidens savadiensis*, 01.iii.2018, **2 specimens** [RMBH Hir_0056_1], Bolotov, Vikhrev, and Nyein Chan leg.; Ayeyarwady River, main channel, 21.9909°N, 96.0610°E, from the mantle cavity of *Lamellidens savadiensis*, 04.iii.2018, **33 specimens** [RMBH Hir_0059_1], Bolotov, Vikhrev, and Nyein Chan leg.; Ayeyarwady Basin, Indaw Lake, 24.2665°N, 96.1228°E, from the mantle cavity of *Lamellidens savadiensis*, 13.xi.2018, **one specimen** [RMBH Hir_0206], Bolotov, Vikhrev, Lopes-Lima, Bogan, and Nyein Chan leg.; Bago River, 17.5334°N, 96.3315°E, from the mantle cavity of *Trapezidens angustior*, 18.ii.2018, **2 specimens** [RMBH Hir_0064_1],

Bolotov, Vikhrev, and Nyein Chan leg.; Ayeyarwady Basin, Sih Khong Stream, 22.0632°N, 96.0810°E, from the mantle cavity of *Lamellidens savadiensis*, 04.iii.2018, **2 specimens** [RMBH Hir_0050], Bolotov, Vikhrev, and Nyein Chan leg.; Middle Sittaung Basin, Myit Kyi Pauk Stream, 18.9613°N, 96.4455°E, from the mantle cavity of *Lamellidens generosus* and *Trapezidens angustior*, 26.xi.2016, **6 specimens** [RMBH Hir_0239], Vikhrev leg.

Material sequenced. Three specimens from the Bago - Sittaung channel, the Sittaung and Salween basins, Myanmar (Supplementary Table 1).

Life style and hosts. This mussel-associated leech species seems to be a host mussel specialist being a possible obligate inhabitant of the mantle cavity of freshwater mussels as a secondary host and shelter in the earlier developmental stages (Supplementary Table 9). It was collected from five freshwater mussel species belonging to one tribe: *Lamellidens ferrugineus*, *L. generosus*, *L. savadiensis*, *Trapezidens dolichorhynchus*, and *T. angustior* (Unionidae: Lamellidentini). Adult leeches use freshwater fishes as the primary hosts: *Clarias* aff. *batrachus* (Siluriformes: Clariidae) and *Oreochromis aureus* (Cichliformes: Cichlidae) (Supplementary Table 8).

Distribution. Bago, Sittaung, Ayeyarwady, and Salween river basins, Myanmar.

Comments. Genetically, this species is most closely related to *Batracobdelloides conchophylus* **sp. nov.** from Myanmar (mean *COI* p-distance = 4.1%).

Batracobdelloides koreanus Bolotov, Klass, Bespalaya, Konopleva, Kondakov & Vikhrev **sp. nov.**

Figs. 3E, 4E, 5E, Table 2, Supplementary Table 4

Type locality. SOUTH KOREA: Seomjin River, 35.7010°N, 127.2845°E, from the mantle cavity of *Nodularia sinuata*.

Type. Holotype RMBH Hir_0116_1-H, in the Russian Museum of Biodiversity Hotspots, Federal Center for Integrated Arctic Research, Russian Academy of Sciences, Arkhangelsk, Russia.

Material examined. SOUTH KOREA: Seomjin River, 35.7010°N, 127.2845°E, from the mantle cavity of *Nodularia sinuata* [RMBH biv0517], 10.vii.2018, **one specimen** [holotype RMBH Hir_0116_1-H], Bogan, Bolotov, Kim, Kondakov, Lopes-Lima, Lee, and Vikhrev leg.; Mangyeong River, irrigation channel, 35.9165°N, 127.7135°E, from the mantle cavity of *Nodularia* sp., 11.vii.2018, **one specimen** [paratype RMBH Hir_0104], Bogan, Bolotov, Kim, Kondakov, Lopes-Lima, Lee, and Vikhrev leg.

Material sequenced. One specimen (paratype) from the Mangyeong River, South Korea (Supplementary Table 1).

Life style and hosts. This mussel-associated leech species seems to be a host mussel specialist being a possible obligate inhabitant of the mantle cavity of freshwater mussels as a secondary host and shelter in the earlier developmental stages

(Supplementary Table 9). It was collected from one freshwater mussel species, *Nodularia sinuata* (Unionidae: Unionini) that seems to be an endemic lineage of South Korea[13]. Adult leeches use freshwater fishes as the primary host: *Channa argus* (Anabantiformes: Channidae) (Supplementary Table 8).

Distribution. Seomjin and Mangyeong river basins, South Korea.

Comments. This species appears to be rare, as it is known from the two type specimens. Genetically, this species is most closely related to *Batracobdelloides hlaingbweensis* **sp. nov.** from Myanmar (mean *COI* p-distance = 5.1%).

Batracobdelloides moogi Nesemann & Csänyi, 1995

=*Batracobdelloides moogi* Nesemann & Csänyi (1995): p. 73, figs. 4-8[14].

Type locality. HUNGARY: Kis-Balaton near the Zala River [approx. 46.6652°N, 17.2121°E].

Type. Holotype, body length 16 mm, body wide 9 mm, June 1994, Csanyi & Nesemann leg., in the Hungarian Natural History Museum, Budapest, Hungary[14].

Material sequenced. Not available.

Life style and hosts: Free-living species which uses pulmonate freshwater snails, primarily *Planorbarius corneus* (Planorbidae), as hosts[14,15].

Distribution. Austria, Hungary, Italy, Lithuania, Montenegro, Poland, and Slovakia[14,15,16].

Comments. Morphologically, this species seems to be more closely related to *Batracobdelloides reticulatus* from India[14].

Batracobdelloides reticulatus (Kaburaki, 1921)

=*Glossiphonia reticulata* Kaburaki (1921): p. 700, fig. 2[17].

=*Batracobdelloides reticulatus* (Kaburaki, 1921). – Nesemann & Csänyi (1995): figs. 9-11[14].

Type locality. INDIA: Jullundur [Jalandhar, Punjab][17].

Type. Holotype (by monotypy), probably in the Zoological Survey of India, Kolkata, India.

Material sequenced. Not available.

Distribution. India and Nepal[14,17-19].

Life style and hosts. This mussel-associated leech species seems to be a host mussel specialist being a possible obligate inhabitant of the mantle cavity of freshwater mussels as a secondary host and shelter in the earlier developmental stages. It appears

to be associated with freshwater mussels in the genus *Lamellidens* (Unionidae: Lamellidentini)[17,18]. Adult leeches probably use freshwater fishes as the primary host.

Comments. Based on biogeographic and morphological evidence, we assume that this species may sister to *Batracobdelloides* mussel leech taxa from Myanmar. It was considered a rare species[18], probably due to its hidden life style inside the mantle cavity of freshwater mussels.

Batracobdelloides tricarinatus (Blanchard, 1897)

=*Helobdella tricarinata* Blanchard (1897): p. 4[20].

=*Batracobdelloides tricarinata* (Blanchard, 1897). – Oosthuizen in Sawyer (1986): p. 659[21].

Figs. 3F, 4F, 5F, Supplementary Fig. 10E

Type locality. TANZANIA: Bubu-Bach, Mbani (Ugogo).

Type. Holotype in the Natural History Museum, Berlin, Germany[11].

Material examined. UGANDA: Nile Basin, Lake George, 0.0476°N, 30.1642°E, from the mantle cavity of *Coelatura aegyptiaca* (Unionidae), 05.viii.2018, **15 specimens** [RMBH Hir_138]; Nile Basin, Albert Nile near Pakwach town, 2.4579°N, 31.4964°E, from the mantle cavity of *Aspatharia* sp.1 and *Aspatharia* sp.2 (Iridinidae), 10.viii.2018, **7 specimens** [RMBH Hir_0139, RMBH Hir_141, Hir_143, Hir_144], from the mantle cavity of *Mutela dubia* (Iridinidae), 10.viii.2018, **2 specimens** [RMBH Hir_140], from the mantle cavity of *Chambardia* sp. (Iridinidae), 10.viii.2018, **8 specimens** [RMBH Hir_142], Bolotov, Filippov, Vikhrev, and Gofarov leg.

Material sequenced. Seven specimens from the Albert Nile Basin, Uganda (Supplementary Table 1).

Life style and hosts. This mussel-associated leech species seems to be a host mussel generalist being a possible facultative inhabitant of the mantle cavity of freshwater mussels as a secondary host and shelter in the earlier developmental stages (Supplementary Table 9). It was collected from five freshwater mussel species belonging to two families: *Coelatura aegyptiaca* (Unionidae: Coelaturini), *Aspatharia* sp.1, *Aspatharia* sp.2, *Chambardia* sp., and *Mutela* sp. (Iridinidae). Adult leeches use freshwater fishes as hosts: *Synodontis frontosus* (Siluriformes: Mochokidae) (Supplementary Table 8).

Distribution. Nile Basin and surrounding endorheic freshwater systems in Africa[11]. Records from Israel[11] are rather questionable and may refer to another species.

Comments. Genetically, this species is most closely related to *Batracobdelloides amnicolus* **stat. rev.** from South Africa (mean *COI* p-distance = 5.8%).

Batracobdelloides yaukthwa Bolotov, Klass, Bespalaya, Konopleva, Kondakov & Vikhrev **sp. nov.**

Figs. 3D, 4D, 5D, Table 2, Supplementary Table 4, Supplementary Figs. 7C, 10D

Type locality. MYANMAR: Middle Sittaung Basin, Chain Stream, 17.9769°N, 96.7650°E, from the mantle cavity of *Trapezidens angustior*.

Type. Holotype RMBH Hir_0060_1-H, in the Russian Museum of Biodiversity Hotspots, Federal Center for Integrated Arctic Research, Russian Academy of Sciences, Arkhangelsk, Russia.

Material examined. MYANMAR: Middle Sittaung Basin, Chain Stream, 17.9769°N, 96.7650°E, from the mantle cavity of *Trapezidens angustior*, 20.ii.2018, **19 specimens** [RMBH Hir_0060_1 including the holotype and 3 paratypes], 21.ii.2018, **20 specimens** [RMBH Hir_0062], from the mantle cavity of *Indochinella pugio viridissima*, 20.ii.2018, **5 specimens** [RMBH Hir_0062 including 2 paratypes], 21.ii.2018, **21 specimens** [RMBH Hir_0062], Bolotov, Vikhrev, and Nyein Chan leg.; Ayeyarwady Basin, Pathein River, 17.4567°N, 95.0086°E, from the mantle cavity of *Trapezidens dolichorhynchus*, 01.xii.2018, **2 specimens** [RMBH Hir_0208], Bolotov, Vikhrev, Lopes-Lima, Bogan, and Nyein Chan leg.

Material sequenced. Three specimens from the Sittaung and Ayeyarwady basins, Myanmar (Supplementary Table 1).

Life style and hosts. This mussel-associated leech species seems to be a host mussel specialist being a possible obligate inhabitant of the mantle cavity of freshwater mussels as a secondary host and shelter in the earlier developmental stages (Supplementary Table 9). It was collected from three freshwater mussel species belonging to two tribes: *Trapezidens angustior*, *T. dolichorhynchus* (Unionidae: Lamellidentini), and *Indochinella pugio viridissima* (Unionidae: Indochinellini). Adult leeches use freshwater fishes as the primary host: *Macrognathus dorsiocellatus* (Synbranchiformes: Mastacembelidae) (Supplementary Table 8).

Distribution. Ayeyarwady and Sittaung river basins, Myanmar.

Comments. Genetically, this species is most closely related to *Batracobdelloides hlaingbweensis* **sp. nov.** from Myanmar (mean *COI* p-distance = 3.8%).

Genus *Hemiclepsis* Vejdovsky, 1884

Type species: *Hirudo marginata* O. F. Müller, 1774 (by subsequent designation)

Hemiclepsis asiatica Moore, 1924 **stat. rev.**

=*Hemiclepsis marginata asiatica* Moore (1924): P. 359, Pl. XXI, fig. 24[22].

Type locality. INDIA: Srinagar, Kashmir.

Type. Probably in the Zoological Survey of India, Kolkata, India[18].

Material sequenced. Not available.

Distribution. North India: Himachal Pradesh and Jammu and Kashmir[18].

Life style and hosts. Free-living species which uses freshwater fishes as hosts[23].

Comments. Moore[22] introduced this peculiar taxon as a subspecies based on the known records of *Hemiclepsis marginata* from India, China, and Japan. However, these records refer to other species in this genus. Based on biogeographic and morphological evidence, we consider it as a separate species-level taxon, *H. asiatica* **stat. rev.** This species has two pairs of eyes[22].

Hemiclepsis bhatiai Baugh, 1960

=*Hemiclepsis bhatiai* Baugh (1960): p. 291, figs. 3-4[24].

Type locality. INDIA: a rocky pool "Sitkundi" in Kalipahar, about 7 miles south-west of Monghyr District (Bihar)[24].

Type. Holotype W 3764/1, in the Zoological Survey of India, Kolkata, India[18,24].

Material sequenced. Not available.

Distribution. India: Bihar and Jammu and Kashmir[18,25].

Life style and hosts. Free-living species, hosts of which are unknown[18,24,26]. The type series was collected in a rocky pool, beneath stones[24].

Comments. This species has three pairs of cup-shaped eyes, which are arranged in two longitudinal rows[24].

Hemiclepsis charwardamensis Mandal, 2013

=*Hemiclepsis charwardamensis* Mandal (2013): p. 155, figs. 1-4[27].

=*Hemiclepsis chharwardamensis* Mandal (2013) **syn. nov.**: p. 153[27].

=*Hemiclepsis ischharwardamensis* Mandal (2013) **syn. nov.**: p. 153[27].

Type locality. INDIA: Charwardam [=Garga Dam?], Bokaro, Jharkhand [approx. 23.6345°N, 86.0724°E][27].

Type. Holotype ZSI An3662/1, in the Zoological Survey of India, Kolkata, India[27].

Material sequenced. Not available.

Distribution. Known only from the type locality.

Life style and hosts. Free-living species. The holotype was collected from a reservoir. The author notes that: "It is found in lake, sucks the blood of mollusc and fishes"[27]. However, this statement on its host range seems to a rather speculative assumption, as the author had only the holotype for description[27], and the hosts of this species appear to be unknown.

Comments. In the protologue, the author mentioned his new species under three different names[27]. Our first reviser action on the precedence of simultaneous synonyms is as follows: *Hemiclepsis charwardamensis* Mandal, 2013 over *H. chharwardamensis* Mandal, 2013 **syn. nov.** and *H. ischharwardamensis* Mandal, 2013 **syn. nov.** We chose *Hemiclepsis charwardamensis* Mandal, 2013 as the valid name, because it seems to be linguistically correct. The original description of this species is very laconic, with four photos illustrating a living specimen, i.e. a general view of its body and head (both from dorsal and ventral sides). This species has two pairs of eyes[27].

Hemiclepsis erhaiensis Yang, 1981

=*Hemiclepsis erhaiensis* Yang (1981): p. 27-30, fig. 2[28].

Type locality. CHINA: Erhai Lake, Yunnan[29].

Type. Whereabouts unknown.

Material sequenced. Not available.

Distribution. Erhai, Dianchi and Chenghai lakes, Yunnan, China[29].

Life style and hosts. Free-living species, which uses freshwater fishes as hosts[29].

Comments. This species seems to be a local endemic lineage of the ancient lakes in Yunnan. It has three pairs of eyes[29].

Hemiclepsis guangdongensis Tan & Liu, 2001

=*Hemiclepsis guangdongensis* Tan & Liu (2001): p. 289-291, figs. 1-2[30].

Type locality. CHINA: Guangzhou City (23.02°N, 113.03°E), Guangdong Province[30].

Type. Whereabouts unknown.

Material sequenced. Not available.

Distribution. Lower Pearl River basin, southeastern China[30].

Life style and hosts. Free-living species, which uses the Amboina Box Turtle *Cuora amboinensis* (Geoemydidae) as a host[30].

Comments. This species may represent an endemic lineage of the Lower Pearl Basin in China. It has one pair of eyes[30].

Hemiclepsis hubeiensis Yang, 1981

=*Hemiclepsis hubeiensis* Yang (1981): p. 27-30, fig. 1[28].

Type locality. CHINA: Sanshan Lake in Huangzhou District, Yangtze Basin, Hubei Province[29].

Type. Whereabouts unknown.

Material sequenced. Not available.

Distribution. Sanshan Lake in Huangzhou District and Ya'er Lake in Ezhou District, Yangtze Basin, Hubei Province, China[29].

Life style and hosts. Free-living species, which uses freshwater fishes as hosts[29].

Comments. This species may represent an endemic lineage of the Yangtze Basin in China. It has two pairs of eyes[29].

Hemiclepsis japonica (Oka, 1932)

=*Placobdella japonica* Oka (1932): p. 51, figs. A, B, C[31].

=*Hemiclepsis japonica* (Oka, 1932). – Soos (1967a): p. 237[26].

Type locality. JAPAN: Inokasira Pond near Tokyo, Honshu, and one specimen from Sapporo, Hokkaido[31].

Type. Whereabouts unknown.

Material sequenced. Not available.

Distribution. Honshu and Hokkaido Islands, Japan.

Life style and hosts. Free-living species, hosts of which are unknown[31].

Comments. This species seems to be a rare lineage endemic to Japan[31]. It has two pairs of eyes[31].

Hemiclepsis kasmiana Oka, 1910 **comb. rev.**

=*Hemiclepsis kasmiana* Oka (1910): p. 169[32].

=*Hemiclepsis casmiana* Oka (1917): p. 167[33].

=*Batracobdella kasmiana* (Oka, 1910). – Soos (1967a): p. 233[26], Soos (1967b): p. 255[34].

=*Hemiclepsis marginata casmiana* Oka (1917). – Lukin (1976): p. 223[35].

Figs. 3G-H, 4 G-H, 5G-H, Supplementary Figs. 9A-C, 10F

Type locality. JAPAN: "Hondo (Kasumiga-Ura, Owari, Bizen)" [Honshu: Lake Kasumigaura, Owari Province, and Bizen city][32].

Type. Whereabouts unknown.

Material examined. JAPAN: Honshu Island, Hyakuken Basin, irrigation ditch, 34.6892°N, 133.9644°E, 01.viii.2002, from the mantle cavity of *Nodularia douglasiae*, **31 specimens** [NCSM-NMI 29630-33], from the mantle cavity of *Inversunio yanagawensis*, **54 specimens** [NCSM-NMI 29634-38], from the mantle cavity of *Pronodularia japanensis*, **88 specimens** [NCSM-NMI 29639-42], from the mantle cavity of *Obovalis omiensis*, **67 specimens** [NCSM-NMI 29643-47], F. Fukuda, K. Suzukida, A. Tamaki et al. leg. RUSSIA: Primorye Region, Artemovka River near Shtykovo village, 43.3779°N, 132.3696°E, from the mantle cavity of *Middendorffinaia mongolica*, 27.x.2000, **13 specimens** [RMBH Hir_0016], Shedko leg.; the same locality and mussel host species, 25.iv.2002, **1 specimen** [RMBH Hir_0237], Barabantchikov leg.; the same locality and mussel host species, 02.vi.2003, **6 specimens** [RMBH Hir_0019], Prozorova leg.; Primorye Region, Solenoye Lake in Khasan District, 42.7045°N, 130.7216°E, from the mantle cavity of *Buldowskia suifunica*, 22.vi.2017, **3 specimens** [RMBH Hir_0046], from the mantle cavity of *Sinanodonta lauta*, same date, **1 specimen** [RMBH Hir_0047], Kolpakov leg.; Primorye Region, Razdolnaya River basin, Komarovka River, 43.6392°N, 132.1614°E, 23.vi.2012, from the mantle cavity of *Margaritifera dahurica* (Margaritiferidae), **8 specimens** [RMBH Hir_0015_5], Bespalaya leg.; Primorye Region, Razdolnaya River basin, Soldatskoye Lake, 3.7747°N, 131.9406°E, 24.x.2016, from the mantle cavity of *Buldowskia suifunica* (Unionidae) [RMBH biv0227], **25 specimens** [RMBH Hir_0015_3], Bolotov and Vikhrev leg.; Primorye Region, Gladkaya River, 42.7065°N, 130.9084°E, 25.x.2016, from the mantle cavity of *Sinanodonta lauta* and *Buldowskia suifunica* (Unionidae) [RMBH biv0225], **190 specimens** [RMBH Hir_0015_1, Hir_0015_2], from the mantle cavity of *Middendorffinaia mongolica* (Unionidae) [RMBH biv0229], **57 specimens** [RMBH Hir_0015_4], Bolotov and Vikhrev leg.; Primorye Region, Gladkaya River, 42.7065°N, 130.9084°E, 28.vi.2018, from the mantle cavity of *Buldowskia suifunica* (Unionidae), **69 specimens** [RMBH Hir_0127], from the mantle cavity of *Middendorffinaia mongolica* (Unionidae), **53 specimens** [RMBH Hir_0128], from the mantle cavity of *Sinanodonta lauta* (Unionidae), **11 specimens** [RMBH Hir_0126], Bogan, Lopes-Lima, Vikhrev, and Kondakov leg.; Khabarovsk Region, Amur Basin, Ulbinka River, 49.9535°N, 136.6319°E, 21.vii.2014, from the mantle cavity of *Nodularia douglasiae* (Unionidae) [RMBH biv0134_1], **2 specimens** [RMBH Hir_0096], Bolotov and Vikhrev leg.; Primorye Region, Khanka Lake basin, Komissarovka River mouth, 44.8255°N, 132.0456°E, 02.vii.2018, from the mantle cavity of *Nodularia douglasiae* (Unionidae) [RMBH biv0503], **30 specimens** [RMBH Hir_0105], from the mantle cavity of Cristaria plicata (Unionidae), **3 specimens** [RMBH Hir_0129_1, Hir_129_2], beneath stones, **1 specimen** [Hir_0111_5], Bogan, Bolotov, Lopes-Lima, Vikhrev, and Kondakov leg.; Primorye Region, Khanka Lake basin, Melgunovka River, 44.5939°N, 132.1818°E, 01.vii.2018, from the mantle cavity of *Buldowskia shadini* and *Sinanodonta schrenckii* (Unionidae) [RMBH biv497 and biv0496], **3 specimens** [RMBH Hir_0102, Hir_0122], from the mantle cavity of *Nodularia douglasiae* (Unionidae) [RMBH biv0498], **2 specimens** [RMBH Hir_0123_1], Bogan, Bolotov, Lopes-Lima, Vikhrev, and Kondakov leg. SOUTH KOREA: Geum River, 35.9891°N, 127.5836°E, 08.vii.2018, from the mantle cavity of *Aculamprotula koreana* (Unionidae) [RMBH

biv0514], **3 specimens** [RMBH Hir_0106], from the mantle cavity of *Lamprotula gottschei* (Unionidae) [RMBH biv0511], **3 specimens** [RMBH Hir_0107], from the mantle cavity of *Nodularia* sp. (Unionidae) [RMBH biv0513], **24 specimens** [RMBH Hir_0124], beneath stones, **3 specimens** [Hir_103_4], Bogan, Bolotov, Kim, Kondakov, Lee, Lopes-Lima, and Vikhrev leg.; Geum River, 36.0708°N, 127.5891°E, 08.vii.2018, beneath stones, **8 specimens** [Hir_0110_6], Bolotov and Kondakov leg.; Seomjin River, 35.7010°N, 127.2845°E, 10.vii.2018, from the mantle cavity of *Nodularia douglasiae* (Unionidae) [RMBH biv0518], **7 specimens** [RMBH Hir_0117], from the mantle cavity of *Nodularia sinuata* (Unionidae) [RMBH biv0517], **52 specimens** [RMBH Hir_0116], from the mantle cavity of *Sinanodonta lauta* (Unionidae) [RMBH biv0519], **19 specimens** [RMBH Hir_0118], Bogan, Bolotov, Kim, Kondakov, Lee, Lopes-Lima, and Vikhrev leg.; Seomjin River, 35.4217°N, 127.2228°E, 10.vii.2018, from the mantle cavity of *Nodularia douglasiae* (Unionidae) [RMBH biv0522], **19 specimens** [RMBH Hir_0119], from the mantle cavity of *Nodularia sinuata* (Unionidae), **26 specimens** [RMBH Hir_0125], beneath stones, **10 specimens** [Hir_0113_2], the same collectors; Mangyeong River, irrigation channel, 35.9165°N, 127.7135°E, 11.vii.2018, from the mantle cavity of *Sinanodonta lauta* (Unionidae) [RMBH biv0523], **1 specimen** [RMBH Hir_0120], from the mantle cavity of *Nodularia sinuata* (Unionidae) [RMBH biv0524], **2 specimens** [RMBH Hir_0121], the same collectors; Han River basin, Bukhang River, 38.0988°N, 127.6957°E, 06.vii.2018, from the mantle cavity of *Nodularia* sp. (Unionidae) [RMBH biv0509], **1 specimen** [sample was lost], the same collectors.; Han River basin, Jichon Stream, 38.0510°N, 127.6532°E, 06.vii.2018, beneath stones, **2 specimens** [Hir_0112_3], Bolotov and Kondakov leg.

Material sequenced. 14 specimens from Russia and 13 specimens from South Korea (Supplementary Table 1).

Life style and hosts. This mussel-associated leech species seems to be a host mussel generalist being a possible obligate inhabitant of the mantle cavity of freshwater mussels as a secondary host and shelter in the earlier developmental stages (Supplementary Table 9). It was collected from 14 freshwater mussel species belonging to two families: *Margaritifera dahurica* (Margaritiferidae: Margaritiferinae), *Buldowskia shadini*, *B. suifunica*, *Cristaria plicata*, *Sinanodonta lauta*, *S. schrenckii* (Unionidae: Cristariini), *Aculamprotula koreana*, *Inversunio yanagawensis*, *Middendorffinaia mongolica*, *Nodularia douglasiae*, *N. sinuata* (Unionidae: Unionini), *Lamprotula gottschei*, *Pronodularia japanensis* (Unionidae: Lamprotulini), and *Obovalis omiensis* (Unionidae: Gonideini). Adult leeches use freshwater fishes as the primary host: *Perccottus glenii* (Perciformes: Odontobutidae) and *Silurus asotus* (Siluriformes: Siluridae) (Supplementary Table 8).

Distribution. This species is widespread through Russian Far East, Korea, Japan, and China. We found that it includes several deeply divergent phylogenetic lineages from the Russian Far East and Korea that may represent separate subspecies-level taxa (Fig. 2). Molecular sequences for specimens from Japan, continental China and Taiwan are still lacking, but these lineages may also share a high genetic divergence from each other.

Comments: This species was originally described as a member of the genus *Hemiclepsis*[32]. Moore[22] noted that *Hemiclepsis kasmiana* closely resembles *H. asiatica*

from northern India by markings pattern and indistinct papillae and that these two forms differ by the number of eyes, i.e. one pair in *H. kasmiana* (total reduction of the anterior pair of eyes) and two pairs in *H. asiatica* (anterior pair being strongly reduced). Additionally, it was suggested that *Hemiclepsis kasmiana* is a two-eyed subspecies of *H. marginata* representing an example of extreme reduction of the anterior part of eyes[22]. Later, Oka[36] re-described this species and found that some live specimens of *H. kasmiana* have the reduced anterior pair of eyes, which was completely lost in ethanol-preserved specimens. Based on this data, Oka[36] confirmed close relation between *Hemiclepsis kasmiana* and the subspecies of Moore but mentioned that *H. kasmiana* differs from *H. marginata asiatica* by a specific "endoparasitic" life style. Specimens of *Hemiclepsis asiatica* were collected from various water bodies, often from surface of stones[22], and this taxon seems to be a fish parasite[23]. Later, *Hemiclepsis kasmiana* was transferred to the genus *Batracobdella* Yiguier, 1879 as a *species inquirendum*, but this taxonomic action has been done without any explanation[26,34]. Bolotov et al.[37] assumed that *Batracobdella kasmiana* is a member of its original genus, *Hemiclepsis*, based chiefly on an external resemblance between *B. kasmiana* and *H. marginata*, i.e. the position of eyes, general body morphology, and clearly expressed markings pattern. Based on the novel molecular data, we finally transfer this species back to *Hemiclepsis* and propose *H. kasmiana* **comb. rev.** Genetically, this species is most closely related to *Hemiclepsis khankiana* **sp. nov.** from the Khanka Lake basin, Russian Far East (mean *COI* p-distance = 3.8%).

Hemiclepsis khankiana Bolotov, Klass, Bespalaya, Konopleva, Kondakov & Vikhrev **sp. nov.**

Figs. 3I, 4I, 5I, Table 2, Supplementary Table 4, Supplementary Fig. 8A

Type locality. RUSSIA: Primorye Region, Khanka Lake Basin, Melgunovka River, 44.5804°N, 132.0803°E, from the mantle cavity of *Nodularia douglasiae*.

Type. Holotype RMBH Hir_0101-H, in the Russian Museum of Biodiversity Hotspots, Federal Center for Integrated Arctic Research, Russian Academy of Sciences, Arkhangelsk, Russia.

Material examined. RUSSIA: Primorye Region, Khanka Lake Basin, Melgunovka River, 44.5804°N, 132.0803°E, from the mantle cavity of *Nodularia douglasiae*, 01.vii.2018, **154 specimens** [RMBH Hir_0101 including the holotype and 3 paratypes], Bolotov, Vikhrev, and Kondakov leg.; Melgunovka River, 44.5939°N, 132.1818°E, from the mantle cavity of *Nodularia douglasiae*, 01.vii.2018, **one specimen** [paratype RMBH Hir_0123_2], Bolotov, Vikhrev, and Kondakov leg.; Spasovka River near Gayvoron village, 44.7565°N, 132.7643°E, 13.viii.2016, from the mantle cavity of *Nodularia douglasiae*, **2 specimens** [RMBH Hir_0018 including one paratype], Sayenko leg.

Material sequenced. Three specimens from Russia (Supplementary Table 1).

Life style and hosts. This mussel-associated leech species seems to be a host mussel specialist being a possible obligate inhabitant of the mantle cavity of freshwater

mussels as a secondary host and shelter in the earlier developmental stages (Supplementary Table 9). It was collected from one species, *Nodularia douglasiae* (Unionidae: Unionini) that is widespread throughout Russian Far East, Korea, Japan, eastern China and northern Vietnam[38]. Adult leeches use freshwater fishes as the primary host: *Rhodeus uyekii* (Mori, 1935) (Cypriniformes: Cyprinidae) (Supplementary Table 8).

Distribution. Khanka Lake Basin, Amur River system, Russian Far East and probably China.

Comments. This species has two pairs of eyes. Genetically, it is most closely related to *Hemiclepsis kasmiana* **comb. rev.** from East Asia (mean *COI* p-distance = 3.8%).

Hemiclepsis marginata (O. F. Müller, 1774)

=*Hirudo marginata* O. F. Müller (1774): p. 46[39].

=*Hemiclepsis marginata* (O. F. Müller, 1774). – Soos (1967a): p. 237[26].

Type locality. In rivo rara [somewhere in Europe].

Type. Whereabouts unknown.

Material examined. RUSSIA: Moscow Region, Volga River basin, a reservoir on the Lopastnya River, 55.2555°N, 37.1744°E, 10.x.2017, **1 specimen** [RMBH Hir_24_1], Palatov leg.; Moscow Region, Volga River basin, a reservoir near Pokrovskoye village, 55.3259°N, 37.2066°E, 10.x.2017, **1 specimen** [RMBH Hir_20_1], Palatov leg.; Moscow Region, Volga River basin, a pond near Starosyrovo village, 55.4909°N, 37.5275°E, 13.xi.2017, **1 specimen** [RMBH Hir_25_3], Palatov leg.; Moscow Region, Volga River basin, a stream near Vaulino village, 56.5333°N, 38.2438°E, 12.vi.2018, **1 specimen** [RMBH Hir_160_2], Palatov leg. KAZAKHSTAN: Irtysh - Ob' River basin, Lower Taynty Reservoir, 49.4418°N, 83.0585°E, 29.vi.2018, **10 specimens** [RMBH Hir_151_2], Palatov and Vinarski leg.

Material sequenced. Four specimens from Russia and one specimen from Kazakhstan (Supplementary Table 1).

Distribution. Europe and Siberia up to the Yenisei Basin and Baikal Lake. Records of this species from East, Southeast and South Asia[18,26,29,40] refer to other taxa, e.g. *Hemiclepsis schrencki* **sp. nov.**, *H. tumniniana* **sp. nov.**, and several undescribed species.

Life style and hosts. Free-living species, which uses freshwater fishes, amphibian larvae, and freshwater molluscs as hosts[26].

Comments. This species has two pairs of eyes. It shares low genetic diversity throughout its enormous range from Europe to the Baikal Lake basin in Eastern Siberia. Genetically, this species is most closely related to *Hemiclepsis kasmiana* **comb. rev.** from East Asia (mean *COI* p-distance = 10.0%).

Hemiclepsis myanmariana Bolotov, Klass, Bespalaya, Konopleva, Kondakov & Vikhrev **sp. nov.**

Figs. 3J, 4J, 5J, Table 2, Supplementary Table 4, Supplementary Figs. 8B, 10G

Type locality. MYANMAR: Salween Basin, Nadi Lake, 20.6858°N, 96.9316°E, from the mantle cavity of *Lamellidens savadiensis*.

Type. Holotype RMBH Hir_0048_1-H, in the Russian Museum of Biodiversity Hotspots, Federal Center for Integrated Arctic Research, Russian Academy of Sciences, Arkhangelsk, Russia.

Material examined. MYANMAR: Salween Basin, Nadi Lake, 20.6858°N, 96.9316°E, from the mantle cavity of *Lamellidens savadiensis*, 23.ii.2018, **117 specimens** [RMBH Hir_0048_1 including the holotype and 3 paratypes], Bolotov, Vikhrev, and Nyein Chan leg.; Ayeyarwady Basin, ox-bow lake near Ta Naung Taig village, 21.4064°N, 95.3399°E, from the mantle cavity of *Lamellidens savadiensis*, 03.iii.2018, **one specimen** [RMBH Hir_0051], Bolotov, Vikhrev, and Nyein Chan leg.; Ayeyarwady Basin, Nga Wun River near Pyay town, 18.8624°N, 95.2822°E, from the mantle cavity of *Lamellidens savadiensis*, 11.xii.2018, **2 specimens** [paratypes RMBH Hir_0210, Hir_0211], Bolotov, Vikhrev, Lopes-Lima, Bogan, and Nyein Chan leg.; Bilin Basin: Shwe Laung Lake near Pyintha village, 17.4395°N, 97.2457°E, from the mantle cavity of *Lamellidens generosus*, 13.ii.2018, **one specimen** [RMBH Hir_0052], Bolotov, Vikhrev, and Nyein Chan leg.; Lower Sittaung Basin: Pangaing Stream, 17.7080°N, 96.7155°E, from the mantle cavity of *Lamellidens generosus*, 20.ii.2018, **one specimen** [RMBH Hir_0054], Bolotov, Vikhrev, and Nyein Chan leg.; Middle Sittaung Basin: Mone Ding Dam outlet, 20.8099°N, 95.7242°E, from the mantle cavity of *Lamellidens savadiensis*, 01.iii.2018, **one specimen** [RMBH Hir_0056_2], from the mantle cavity of *Indonaia andersoniana*, 01.iii.2018, **one specimen** [RMBH Hir_0099], Bolotov, Vikhrev, and Nyein Chan leg.; Middle Sittaung Basin: Sin Thay Dam outlet, 20.1540°N, 96.1149°E, from the mantle cavity of *Lamellidens savadiensis*, 01.iii.2018, **one specimen** [RMBH Hir_0061], Bolotov, Vikhrev, and Nyein Chan leg.

Material sequenced. Six specimens from the Ayeyarwady, Sittaung, Bilin, and Salween basins, Myanmar (Supplementary Table 1).

Life style and hosts. This mussel-associated leech species seems to be a host mussel specialist being a possible obligate inhabitant of the mantle cavity of freshwater mussels as a secondary host and shelter in the earlier developmental stages (Supplementary Table 9). It was collected from three freshwater mussel species belonging to two tribes: *Lamellidens savadiensis*, *L. generosus* (Unionidae: Lamellidentini) and *Indonaia andersoniana* (Unionidae: Indochinellini). Most our samples come from *Lamellidens savadiensis*. Adult leeches use freshwater fishes as the primary host: *Labeo rohita* (Cypriniformes: Cyprinidae) (Supplementary Table 8).

Distribution. Ayeyarwady, Sittaung, Bilin, and Salween river basins, Myanmar.

Comments. This species has two pairs of eyes. Genetically, it is most closely related to *Hemiclepsis khankiana* **sp. nov.** from the Khanka Lake basin, Russian Far East (mean *COI* p-distance = 7.1%).

Hemiclepsis schrencki Bolotov, Klass, Bespalaya, Konopleva, Kondakov & Vikhrev **sp. nov.**

Figs. 3K, 4K, 5K, Table 2, Supplementary Table 4, Supplementary Fig. 10H

Type locality. RUSSIA: Primorye Region, Partizanskaya River, 43.0585°N, 133.1540°E.

Type. Holotype RMBH Hir_0091_1-H, in the Russian Museum of Biodiversity Hotspots, Federal Center for Integrated Arctic Research, Russian Academy of Sciences, Arkhangelsk, Russia.

Material examined. RUSSIA: Primorye Region, Partizanskaya River, 43.0585°N, 133.1540°E, 27.v.2017, **one specimen** [holotype RMBH Hir_0091_1-H], Bolotov leg.; Primorye Region, Ussuri Basin, Muravievka River, 43.7703°N, 133.2611°E, on a stone, 19.v.2017, **one specimen** [paratype RMBH Hir_0088_1], Bolotov leg.

Material sequenced. Two specimens from Russia (Supplementary Table 1).

Life style and hosts. Free-living species, which uses freshwater fishes as hosts: *Barbatula toni* (Dybowski, 1869) (Cypriniformes: Nemacheilidae) and *Phoxinus* sp. “Amur” (Cypriniformes: Cyprinidae) (Supplementary Table 8).

Distribution. Partizanskaya and Ussuri river basins, Russian Far East.

Comments. This species has two pairs of eyes. Genetically, it is most closely related to *Hemiclepsis khankiana* **sp. nov.** from Khanka Lake Basin, Russian Far East (mean *COI* p-distance = 9.1%).

Hemiclepsis tumniniana Bolotov, Klass, Bespalaya, Konopleva, Kondakov & Vikhrev **sp. nov.**

Figs. 3L, 4L, 5L, Table 2, Supplementary Table 4, Supplementary Figs. 8C, 10I

Type locality. RUSSIA: Khabarovsk Region, Tumnin River, 50.0001°N, 139.9175°E.

Type. Holotype RMBH Hir_0093-H, in the Russian Museum of Biodiversity Hotspots, Federal Center for Integrated Arctic Research, Russian Academy of Sciences, Arkhangelsk, Russia.

Material examined. RUSSIA: Khabarovsk Region, Tumnin River, 50.0001°N, 139.9175°E, silty-gravel bottom with macrophytes and algae, on stones, 17.vii.2014, **4 specimens** [holotype RMBH Hir_0093-H, paratypes RMBH Hir_0014, Hir_0235], Bolotov and Vikhrev leg.; Tumnin River, 49.9451°N, 139.9181°E, 14.vii.2014, **6 specimens** [paratypes RMBH Hir_0001], Bolotov and Vikhrev leg.

Material sequenced. Five specimens from Russia (Supplementary Table 1).

Life style and hosts. Free-living species, which uses freshwater fishes as hosts: *Pungitius pungitius* (Gasterosteiformes: Gasterosteidae) (Supplementary Table 8).

Distribution. Tumnin River, Khabarovsk Region, Russia.

Comments. This species has two pairs of eyes. Genetically, it is most closely related to *Hemiclepsis kasmiana* **comb. rev.** from East Asia (mean *COI* p-distance = 9.5%).

Hemiclepsis viridis Chelladurai, 1934

=*Hemiclepsis viridis* Chelladurai (1934): p. 345, figs. 1-4[41].

Type locality. INDIA: Trivandrum [Kerala] and Ootacamund [Udagamandalam, Tamil Nadu][41].

Type. Probably in the Zoological Survey of India, Kolkata, India[41].

Material sequenced. Not available.

Distribution. Kerala and Tamil Nadu states in India[18,25,41].

Life style and hosts. Free-living species, which uses frogs as hosts[26,41].

Comments. This species has three pairs of eyes[41].

Key to mussel-associated leech species (Glossiphoniidae: *Batracobdelloides* and *Hemiclepsis*) of the Old World

1	First pair of eyes is small but visible, and is located ahead of the second pair of eyes………………………	2
–	First pair of eyes is completely or almost completely reduced or joined with the second pair of eyes………	5
2	Dorsum with six longitudinal rows of small tubercles<br>Mussel hosts: *Lamellidens*. Range: North India and Nepal	*Batracobdelloides reticulatus* (Kaburaki, 1921)
–	Dorsum smooth………………………………………………	3
3	Dorsum yellowish or whitish (dark yellow in living animals), with clear longitudinal brown stripes, or dorsum completely brown with longitudinal rows of white spots……………………………………………………	4
–	Dorsum yellowish, brownish or whitish (dark yellow in living animals), sometimes with unclear longitudinal narrow light brown stripes and rows of light brown dashes<br>Mussel hosts: *Lamellidens* and *Indonaia* (Unionidae: Parreysiinae). Range: Myanmar (Ayeyarwady, Sittaung, Bilin, and Salween basins)	*Hemiclepsis myanmariana* **sp. nov.**
4	Dorsum yellowish or whitish (dark yellow in living animals), with six longitudinal broad, smooth brown stripes<br>Mussel hosts: *Nodularia douglasiae* (Unionidae: Unioninae). Range: Khanka Lake basin in eastern Russia and probably China	*Hemiclepsis khankiana* **sp. nov.**
–	Dorsum yellowish or whitish (dark yellow in living animals), with six narrower, broken longitudinal stripes formed by differently sized brown spots or brown dorsum with rows of small white spots<br>Mussel hosts: *Aculamprotula*, *Buldowskia*, *Cristaria*, *Inversunio*, *Middendorfinaia*, *Nodularia*, *Sinadonta* (Unionidae: Unioninae), *Lamprotula*, *Pronodularia*, *Obovalis* (Unionidae: Gonideinae), *Margaritifera* (Margaritiferidae). Range: widespread throughout Russian Far East, Japan, Korea, and eastern China	*Hemiclepsis kasmiana* **comb. rev.**
5	Eyes joined into one large angulate-ovate spot<br>Mussel hosts: *Radiatula* and *Lamellidens* (Unionidae:	*Batracobdelloides conchophylus* **sp.**

	Parreysiinae). Range: Myanmar (Sittaung and Haungthayaw basins)	**nov.**
–	Eyes smaller, separate or connected via a grey shading……………………………………………………..	6
6	Dorsum with seven longitudinal brown stripes, and weakly developed, almost invisible tubercles<br>Mussel hosts: *Nodularia sinuata* (Unionidae: Unioninae). Range: South Korea	*Batracobdelloides koreanus* **sp. nov.**
–	Dorsum without clear marking pattern, but with well-developed tubercles………………………………………	7
7	Dorsum with one central row of tubercles, and lateral tubercles being broadly scattered………………………	8
–	Dorsum with three regular rows of tubercles…………..	9
8	Tubercles conical, spinous<br>Mussel hosts: *Trapezidens* and *Indochinella* (Unionidae: Parreysiinae). Range: Myanmar (Ayeyarwady and Sittaung basins)	*Batracobdelloides yaukthwa* **sp. nov.**
–	Tubercles rounded, flattened<br>Mussel hosts: *Pseudodon* (Unionidae: Gonideinae). Range: Myanmarv (endemic to Hlaingbwe Basin)	*Batracobdelloides hlaingbweensis* **sp. nov.**
9	Posterior sucker without brown marking pattern or with a few unclear light brown spots<br>Mussel hosts: *Lamellidens* and *Trapezidens* (Unionidae: Parreysiinae). Range: Myanmar (Bago, Sittaung, Ayeyarwady, and Salween basins)	*Batracobdelloides indochinensis* **sp. nov.**
–	Posterior sucker with dense radial brown bands<br>Mussel hosts: *Coelatura* (Unionidae: Parreysiinae), *Aspatharia*, *Chambardia*, and *Mutela* (Iridinidae). Range: Africa (Nile Basin and surrounding endorheic freshwater systems)	*Batracobdelloides tricarinatus* (Blanchard, 1897)

Supplementary References

1. Simon, C. *et al.* Evolution, weighting, and phylogenetic utility of mitochondrial gene sequences and a compilation of conserved polymerase chain reaction primers. *Annals of the Entomological Society of America* **87**, 651–701; DOI:10.1093/aesa/87.6.651 (1994).

2. Lobo, J. *et al.* Enhanced primers for amplification of DNA barcodes from a broad range of marine metazoans. *BMC Ecology* **13**, 1–8; DOI:10.1186/1472-6785-13-34 (2013).

3. Giribet, G., Carranza, S., Baguna, J., Riutort, M. & Ribera, C. First molecular evidence for the existence of a Tardigrada + Arthropoda clade. *Molecular Biology and Evolution* **13**, 76–84; DOI:10.1093/oxfordjournals.molbev.a025573 (1996).

4. Whiting, M.F., Carpenter, J.C., Wheeler, Q.D. & Wheeler, W.C. The *Strepsiptera* problem: phylogeny of the holometabolous insect orders inferred from *18S* and *28S* ribosomal DNA sequences and morphology. *Systematic Biology* **46**, 1–68; DOI:10.1093/sysbio/46.1.1 (1997).

5. Ward, R. D., Zemlak, T. S., Innes, B. H., Last, P. R. & Hebert, P. D. DNA barcoding Australia's fish species. *Philosophical Transactions of the Royal Society of London B: Biological Sciences* **360**, 1847–1857; DOI:10.1098/rstb.2005.1716 (2005).

6. Kumar, S., Stecher, G. & Tamura, K. MEGA7: molecular evolutionary genetics analysis version 7.0 for bigger datasets. *Molecular Biology and Evolution* **33**, 1870–1874; DOI:10.1093/molbev/msw054 (2016).

7. Kalyaanamoorthy, S., Minh, B. Q., Wong, T. K., von Haeseler, A. & Jermiin, L. S. ModelFinder: fast model selection for accurate phylogenetic estimates. *Nature Methods* **14**, 587–589; DOI:10.1038/nmeth.4285 (2017).

8. Nguyen, L. T., Schmidt, H. A., von Haeseler, A. & Minh, B. Q. IQ-TREE: a fast and effective stochastic algorithm for estimating maximum-likelihood phylogenies. *Molecular Biology and Evolution* **32**, 268–274; DOI:10.1093/molbev/msu300 (2014).

9. Bolotov, I. N. *et al.* Ancient river inference explains exceptional Oriental freshwater mussel radiations. *Scientific Reports* **7**, 1–14; DOI:10.1038/s41598-017-02312-z (2017).

10. Moore, J. P. The leeches (Hirudinea) in the collection of the Natal Museum. *Annals of the Natal Museum* **14**, 303–340 (1958).

11. Oosthuizen, J. H. Redescription of the African fish leech *Batracobdelloides tricarinata* (Blanchard, 1897) (Hirudinea: Glossiphoniidae). *Hydrobiologia* **184**, 153–164; DOI:10.1007/BF00027023 (1989).

12. Oosthuizen, J. H. An annotated check list of the leeches (Annelida: Hirudinea) of the Kruger National Park with a key to the species. *Koedoe* **34**, 25–38; DOI:10.4102/koedoe.v34i2.421 (1991).

13. Lee J. H. *Systematic study of Korean unionids (Bivalvia: Unionidae) based on morphological and molecular data. PhD dissertation* (Kyungpook National University, Korea, 2017).

14. Nesemann, H. & Csänyi, B. Description of *Batracobdelloides moogi* n. sp., a leech genus and species new to the European fauna with notes on the identity of *Hirudo paludosa* Carena, 1824 (Hirudinea: Glossiphoniidae). *Lauterbornia* **21**, 69–78 (1995).

15. Bielecki, A. *et al. Batracobdelloides moogi* Nesemann et Csányi, 1995 (Hirudinida: Glossiphoniidae): Morphometry and structure of the alimentary tract and reproductive system. *Biologia* **66**, 848–855; DOI:10.2478/s11756-011-0100-8 (2011).

16. Grosser, C. & Pešić, V. First record of *Batracobdelloides moogi* (Hirudinea: Glossiphoniidae) in the Balkans. *Natura Montenegrina* **4**, 29–32 (2005).

17. Kaburaki, T. Notes on some leeches in the collection of the Indian Museum. *Records of the Indian Museum* **22**, 689–719 (1921).

18. Chandra, M. *The Leeches of India – A Handbook* (Zoological Survey of India, Calcutta, 1991).

19. Nesemann, H. et al. *Aquatic invertebrates of the Ganga river system: Volume 1: Mollusca, Annelida, Crustacea (in part)* (Hasko Nesemann & Chandi Press, Kathmandu, 2007).

20. Blanchard, R. Hirudineen Ost-Afrikas und der Nachbargebiete. *Mobius' Tierwelt Deutsch-Ost-Afrika* **4**, 3–9 (1897).

21. Sawyer, R. T. *Leech biology and behaviour. Vol. 2. Feeding biology, ecology, and systematics* (Clarendon Press, Oxford, 1986).

22. Moore, J. P. Notes on some Asiatic leeches (Hirudinea) principally from China, Kashmir, and British India. *Proceedings of the Academy of Natural Sciences of Philadelphia* **76**, 343–388 (1924).

23. Shrivastav, H. O. P. & Shah, H. L. Occurrence of the leech *Hemiclepsis marginata asiatica* in the bile duct of a pig (*Sus scrofa domestica*). *Indian Veterinary Journal* **48**, 203–204 (1971).

24. Baugh, S. C. Studies on Indian Rhynchobdellid leeches. *Parasitology* **50**, 287–301; DOI:10.1017/S0031182000025403 (1960).

25. Chandra, M. A check-list of leeches of India. *Records of the Zoological Survey of India* **80**, 265–290 (1983).

26. Soos, A. On the genus *Hemiclepsis* Vejdovsky, 1884, with a key and catalogue of the species (Hirudinoidea: Glossiphoniidae). *Opuscula Zoologica (Budapest)* **7**, 233–240 (1967a).

27. Mandal, C. K. *Hemiclepsis chharwardamensis* sp. nov. (Hirudinea: Glossiphonidae) a new species of leech from Jharkhand, India. *Records of the Zoological Survey of India* **113**, 153–155 (2013).

28. Yang, T. Two new species of parasitic leeches from freshwater fishes in China. *Acta Zootaxonomica Sinica* **6**, 27–30 (1981).

29. Yang, T. *Annelida Hirudinea (Fauna Sinica)* (Science Press, Beijing, 1996).

30. Tan, E.-G. & Liu, X.-Q. One new species of the genus *Hemiclepsis* (Rhynchobdellida: Glossiphoniidae). *Acta Zootaxonomica Sinica* **3**, 289–291 (2001).

31. Oka, A. Sur une nouvelle espèce de *Placobdella*, *Pl. japonica* n. sp. *Proceedings of the Imperial Academy* **8**, 51–53 (1932).

32. Oka, A. Synopsis der Japanischen Hirudineen, mit Diagnosen der Neuen Species. *Annotationes Zoologicae Japonenses* **7**, 165–183 (1910).

33. Oka, A. Zoological result of a tour in the Far East: Hirudinea. *Memoirs of the Asiatic Society of Bengal* **6**, 157–176 (1917).

34. Soos, A. On the genus *Batracobdella* Viguier, 1879, with a key and catalogue to the species (Hirudinoidea: Glossiphoniidae). *Annales Historico-Naturales Musei Nationales Hungarici (Pars Zoologica)* **59**, 243–257 (1967b).

35. Lukin, E. I. Leeches of fresh and brackish water bodies. *Fauna of the USSR* **109**, 1–484 (1976).

36. Oka, A. Description de l'*Hemiclepsis kasmiana*. *Ibid* **4**, 64–66 (1928).

37. Bolotov, I. N. et al. Discovery and natural history of the mussel leech *Batracobdella kasmiana* (Oka, 1910) (Hirudinida: Glossiphoniidae) in Russia. *Zootaxa* **4319**, 386–390; DOI:10.11646/Zootaxa.4319.2.11 (2017).

38. Klishko, O. K., Lopes-Lima, M., Froufe, E., Bogan, A. E. & Abakumova, V. Y. Unravelling the systematics of *Nodularia* (Bivalvia, Unionidae) species from eastern Russia. *Systematics and Biodiversity* **16**, 287–301; DOI:10.1080/14772000.2017.1383527 (2018).

39. Müller, O. F. Vermivm terrestrium et fluviatilium, seu, Animalium infusoriorum, helminthicorum, et testaceorum, non marinorum succincta historia. *Havniae et Lipsiae* **1**, 37–51 (1774).

40. Nagasawa, K. & Miyakawa, M. Infection of Japanese eel *Anguilla japonica* Elvers by *Hemiclepsis marginata* (Hirudinida: Glossiphoniidae). *Journal of the Graduate School of Biosphere Science, Hiroshima University* **45**, 15–19 (2006).

41. Chelladurai, J. E. On a new Indian leech *Hemiclepsis viridis* sp. nov. *Records of the Indian Museum* **36**, 345–352 (1934).
